# Structure, Function, Regulation and Phylogenetic Relationship of ZIP Family Transporters of Plants

**DOI:** 10.3389/fpls.2020.00662

**Published:** 2020-05-27

**Authors:** T. P. Ajeesh Krishna, T. Maharajan, G. Victor Roch, Savarimuthu Ignacimuthu, Stanislaus Antony Ceasar

**Affiliations:** ^1^Division of Plant Biotechnology, Entomology Research Institute, Loyola College, University of Madras, Chennai, India; ^2^Xavier Research Foundation, St. Xavier’s College, Palayamkottai, India

**Keywords:** ZIP transporters, homology modeling, transcription factor, functional characterization, genetic modification

## Abstract

Zinc (Zn) is an essential micronutrient for plants and humans. Nearly 50% of the agriculture soils of world are Zn-deficient. The low availability of Zn reduces the yield and quality of the crops. The zinc-regulated, iron-regulated transporter-like proteins (ZIP) family and iron-regulated transporters (IRTs) are involved in cellular uptake of Zn, its intracellular trafficking and detoxification in plants. In addition to Zn, ZIP family transporters also transport other divalent metal cations (such as Cd^2+^, Fe^2+^, and Cu^2+^). ZIP transporters play a crucial role in biofortification of grains with Zn. Only a very limited information is available on structural features and mechanism of Zn transport of plant ZIP family transporters. In this article, we present a detailed account on structure, function, regulations and phylogenetic relationships of plant ZIP transporters. We give an insight to structure of plant ZIPs through homology modeling and multiple sequence alignment with *Bordetella bronchiseptica* ZIP (BbZIP) protein whose crystal structure has been solved recently. We also provide details on ZIP transporter genes identified and characterized in rice and other plants till date. Functional characterization of plant ZIP transporters will help for the better crop yield and human health in future.

## Introduction

In agriculture, the low availability of nutrients has reduced the crop production. The optimal supply of micro-nutrients in soil solutions is vital for normal agriculture production. Zinc (Zn) is one of the most essential micronutrients; it is irreplaceable for plant growth and metabolism (Marschner, 1995, 2011). Both deficient and excess Zn has impaired the physiological and biochemical process of the plant (Cakmak, 2000; Marichali et al., 2014). Zn deficiency is one of the most serious problems worldwide reducing quality of crops (Ruel and Bouis, 1998; Sadeghzadeh, 2013), apart from yield loss and the reduced Zn content in grains (Krithika and Balachandar, 2016). Soil Zn deficiency and human Zn deficiency are often closely associated (Cakmak, 2008). Zn deficiency has been a major cause of death for children in many countries (Black et al., 2008). Regmi et al. (2010) reported that the lack of Zn was a major nutritional problem in humans; more than 3 billion world population were affected by various health problems due to low supply of Zn in their food. For example, approximately 50% of the paddy field is Zn deficient and rice grown on these soils usually produces very less yield with poor nutritional quality (Krithika and Balachandar, 2016). It was estimated that the Zn sufficient rice plant had 40 mg/kg of Zn in its grains, whereas under Zn deficient condition it showed only 10 mg/kg of Zn (Wissuwa et al., 2008). All over the world, 50% of agriculture soils are Zn deficient (FAO, 2000). Zn deficiency was observed in a wide range of soil types such as high pH calcareous soils, sandy soils, and high phosphorus (P) fertilized soils (Marschner, 1995). In 1972, the United States considered Zn deficiency as the most common micronutrient deficiency in crops (Lindsay, 1972). Zn deficiency is one of the major limiting factors affecting crop production badly. So, improving crop varieties with Zn efficiency is helpful to overcome the Zn deficiency problem.

To overcome low Zn availability, plants have evolved a complex array of tightly controlled adaptive mechanisms. The Zinc-regulated, Iron-regulated transporter-like Protein (ZIP) family has been identified and characterized in prokaryotes, eukaryotes, and archaeotes and has been validated to be involved in metal uptake and transport including Zn^2+^ (Grotz and Guerinot, 2006; Kavitha et al., 2015). The presence of ZIP transporters in such a diverse organism indicates their pivotal role in Zn homeostasis. The ZIP family has a major role in Zn transport and metal homeostasis in plants. Besides Zn^2+^, ZIP transporters are reported to be involved in the transport of other transition metal cations such as manganese (Mn^2+^), iron (Fe^2+^), cadmium (Cd^2+^), cobalt (Co^2+^), copper (Cu^2+^), and nickel (Ni^2+^) (Pedas and Husted, 2009). The iron-regulated transporters (IRTs) are the major Fe transporters; they are members of the ZIP family transporters (Eide et al., 1996; Conte and Walker, 2011). The AtIRT1 is identified as the key Fe^2+^ transporter in *Arabidopsis.* The AtIRT1 (Eide et al., 1996), AtIRT2 (Vert et al., 2001), and AtIRT3 (Lin et al., 2009) transporters are seem to be functioning for Fe^2+^ uptake and transport in *Arabidopsis*. IRTs of *Arabidopsis* has the ability to transport several divalent metal ions such as Zn^2+^, Mn^2+^, and Cd^2+^ (Eide et al., 1996; Vert et al., 2001; Chiang et al., 2006; Lee and An, 2009; Lin et al., 2009). The IRTs genes are found to be expressing under different metal stress conditions with increased expression levels under Fe deficiency. ZIP transporters balance the uptake, utilization, and storage of Zn^2+^ under Zn stress condition (Ramesh et al., 2003; Palmgren et al., 2008). The ZIP transporters are also located in various cell organelles and they are actively involved in Zn homeostasis and plant’s adaptation to low and high Zn soils (Tiong et al., 2015). In crops, most of the ZIP transporters are poorly understood with only ZIPs of a few plants have been functionally characterized (Kavitha et al., 2015). The detailed analysis of the structure of the plant ZIP transporter is still lacking. Recently, the crystal structure of the ZIP protein was deduced from a bacterium *Bordetella bronchiseptica* and its metal transport mechanism was also predicted (Zhang et al., 2017). So, in this article, we present the details on the structure, mechanism and phylogenetic relationships of plant ZIP transporters. We provided insight into the structure of plant ZIPs through homology modeling and multiple sequence alignment using *B. bronchiseptica* ZIP (BbZIP) protein as a template. These findings would be a valuable theoretical knowledge for future studies on Zn transporters in crops. We also present details on ZIP transporter genes identified and characterized till date in various plants.

## Role of Zn in Crops

Zinc is one of the eight essential micronutrients in plants (Hänsch and Mendel, 2009). In crops, Zn deficiency was first identified in rice on calcareous soils in India (Nene, 1966; Yoshida and Tanaka, 1969). Plant cells require optimum levels of Zn for normal physiological functions. Zn is involved in the maintenance of the structural and functional integrity of biological membrane and facilitation of protein synthesis, gene expression and regulation and defense against disease (Cakmak, 2000; Andreini et al., 2006; Sadeghzadeh, 2013). Also, it is involved as a structural, catalytic, and intracellular and intercellular signaling component and Zn is the only metal required for the activity of all six classes of the enzymes (Sadeghzadeh, 2013). Especially Zn is essential for the activity of metallo-enzymes that are involved in protein and nucleic acid metabolism (Eide, 2006).

Zinc is needed by a small quantity (0.5–2 μM) from the soil for the normal plant function (Krishna et al., 2017), but yet crucial for many physiological and metabolic pathways (Chen et al., 2008). In rice, 1.5 μM Zn is an optimum level for growth on agar nutrient solution (Impa et al., 2013). Zn content less than 15–20 μg per gram of dry leaf tissues of the plant is a sign of Zn deficiency (Mitra, 2015). Zn is actively involved in specific reactions of metabolic pathways, such as tryptophan biosynthesis, which in turn is the precursor of indole-3-acetic acid (IAA) and other phytohormones. In plants, Zn^2+^ ions are directly involved in the synthesis of tryptophan and auxin (Horak and Trčka, 1976; Marschner, 2011). Zn deficiency decreases the level of phytohormones such as auxin, abscisic acid, gibberellins, and cytokinin’s (Kumar et al., 2016). It significantly affects the plant cell division, cell enlargement, and differentiation (Skoog, 1940; Tsui, 1948). Cakmak et al. (1989) observed that under Zn deficient condition, the level of IAA in the shoot tips and young leaves of bean was reduced to 50% of that in Zn sufficient condition. Zn deficiency reduces the plant growth, yield, and quality in crops such as rice (Chen et al., 2008), mungbean (Samreen et al., 2017), and maize (Wang and Jin, 2005). Zn deficiency decreases the activity of key photosynthetic enzymes, namely carbonic anhydrase, (Brown et al., 1993) which is crucial for crop production (Ali et al., 2008; Mousavi, 2011; Xi-wen et al., 2011). The Zn deficiency induces male sterility in maize (Sharma et al., 1987) and wheat (Sharma et al., 1979). Ekiz et al. (1998) noticed that the crops such as wheat, oat, barley, and triticale show significant decrease in growth and grain yield under Zn-deficient conditions. Zn is a not replaceable micro-nutrient for crops. Zn deficiency is a common problem in all parts of the globe. Therefore, much more research is necessary for improving Zn use efficiency in crops for growth and higher yields under Zn deficient soils.

## Role of ZIP Transporters in Zn Transport

In plants, Zn is acquired and transported predominantly as Zn^2+^ (divalent). Zn ions can also be bound with root exudates like malate, citrate, oxalate and other low molecular weight organic acids which aided to move toward the root surface area (Suzuki et al., 2006). The charged Zn^2+^ ion do not freely diffuse across the lipid bilayer membranes (Eide, 2005). Zn first enters the root cell wall’s free space by a diffusion process from the soil solutions (Hacisalihoglu and Kochian, 2003). Transport of Zn^2+^ into the cortex takes place via symplastic or apoplastic pathway (Kumar et al., 2016). The Zn transporter proteins are required to carry Zn^2+^ into the cells and transport out of intracellular compartments. They include a low-affinity (*K*_m_ = 2–5 μM) and high-affinity (*K*_m_ = 0.6–2 nM) membrane transporter systems (Hacisalihoglu et al., 2001; Kumar et al., 2016). The high-affinity transporter system is dominantly active under low Zn soil (Hacisalihoglu et al., 2001). The ZIP and IRT transporters help to carry Zn^2+^ ions across cellular membranes into the cytoplasm (Eide et al., 1996; Eide, 2005; Krishna et al., 2017). After that Zn^2+^ ions pass the casparian band, endodermis and xylem parenchyma cells which subsequently loaded into the xylem. The heavy metal ATPase 2 (HMA2) and HMA4 transporters of P-type ATPase family are involved in xylem loading of Zn from the xylem parenchymatous cells (Hussain et al., 2004; Hanikenne et al., 2008). The ZIP family transporters are mainly involved in up take, transport and distribution of Zn in the whole plant. The presence of ZIP transporters in a diverse organism indicates their importance in Zn transport and homeostasis. Therefore, understanding of their expression levels, localization, and function in crops is essential. The functional genomics and biotechnological approaches can be used to develop Zn deficiency tolerant crops in the future. It assists in improving the quality and quantity of crops especially enrichment of Zn content in grains and to overcome Zn deficiency problems worldwide. The ZIP transporter families can be used for genetic modification in crops to improve the fortification of Zn.

## Structure of the ZIP Transporter

Only a very limited information is available on structural features and mechanism of Zn transport of plant ZIP family transporters. Plant ZIP family transporters are predicted to have 6–9 transmembrane (TM) domains (α-helices) with 8 being the most prevalent form (Guerinot, 2000). The molecular weight of the Zn transporters ranged from 33.1 to 51.4 kDa and protein sequence ranged from 322 to 478 amino acids (Vatansever et al., 2016). Understanding the mechanism of ZIP transporters requires their high-resolution crystal structures. The crystal structure of the plant ZIP protein is not yet available. However, recently, a high-resolution crystal structure of prokaryotic ZIP protein was deduced from a bacterium *B. bronchiseptica* and its metal (Cd^2+^ and Zn^2+^) transport mechanism was also predicted (Zhang et al., 2017). The crystal structure of BbZIP transporter protein showed eight TM domains (TM1–TM8). The structure of BbZIP was deduced with an inward-open confirmation and occluded at the extracellular side with a binuclear metal center located in the center. The eight TMs formed a closely associated α-helix bundle. The first three TMs (TM1–TM3) can be superimposed on the last three TMs (TM6–TM8) by rotating 180°, and TM4 and TM5 are symmetrically related and sandwiched by the two 3 TM repeats (Zhang et al., 2017). The binuclear metal center is formed by TM4 and TM5 with several conserved amino acid residues (His177, Asn178, Pro180, Glu181, and Gly182) and metal-binding motifs (Gln207, Asp/Asn208, Pro210, Glu211, and Gly212). The structure of the BbZIP showed four Cd^2+^ and seven Zn^2+^ metal-binding sites (Zhang et al., 2017). The topology of BbZIP shows that both the N and the C termini are exposed to the extracellular space and the putative metal transport pathway found at the extracellular side is blocked by hydrophobic residues of the TM2 (Met99 and Ala102), TM5 (Leu200 and Iso204), and TM7 (Met269) (Zhang et al., 2017). The invariant Ser106 on TM2 is situated on the bottom of the shallow and negatively charged entrance cavity, it is important for guiding metals into the transport pathway. Two invariant metal-chelating residues Asp113 and Asp305 present in the entrance cavity have been predicted to be crucial for recruiting metal substrates. The BbZIP structure shows multiple conserved metal-binding sites near the metal exit cavity, indicating that these constitute a route of metal release to the cytoplasm. The bound metal released into the cytoplasm through a chain of metal-chelating residues such as His177, Glu276, His275, Pro180, Pro210, and Asp144 (Zhang et al., 2017). The Zn^2+^ binding is penta coordinated by Glu181, Gln207, and Glu211 and two molecules of water (Zhang et al., 2017). This structure provides a model to analyze the plant ZIP transporters. So we have used this as a template to model and analyze the residues in plant ZIP transporters.

### Comparison of Metal Binding Residues in BbZIP and Plant ZIPs

**FIGURE 1:**
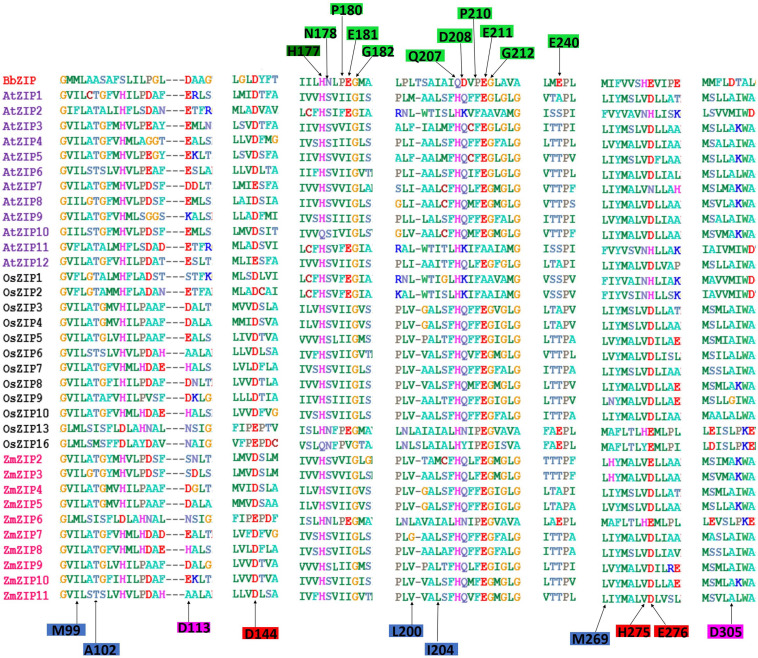
Multiple sequence alignment of *Bordetella bronchiseptica* ZIP (BbZIP), *Arabidopsis* (AtZIP), rice (OsZIP), and maize (ZmZIP) transporters. The protein sequences were aligned by ClustalW alignment using molecular evolutionary genetics analysis, V 6.0 (MEGA6) tool (Tamura et al., 2013). The residues (BbZIP) involved in Cd/Zn binding and transport are highlighted in green and dark green. The His177 highlighted in dark green is involved in both metal recruiting and cytoplasmic transport of metal. The hydrophobic residues involved in blocking of metals at extracellular surface are highlighted in light blue. Metal recruiting residues are highlighted in pink. The residues involved in metal release into the cytoplasm are highlighted in red.

We have analyzed the BbZIP protein sequence with plant ZIP transporters such as *Arabidopsis* (12 ZIPs), rice (12 ZIPs), and maize (10 ZIPs) through a ClustalW alignment (Tamura et al., 2013). The functional residues of the BbZIP and their corresponding plant ZIP residues are presented in Figure 1. We have also included the complete alignment file (Supplementary Figure S1). Except a few, most of the functional residues of *Arabidopsis*, rice and maize ZIP protein sequences are not conserved/homologous with BbZIP. But it is interesting to see that all the plant ZIP protein sequences (expect AtZIP10 and OsZIP16) have conserved His117 residue found in BbZIP (Figure 1). In AtZIP10 (Gln222) and OsZIP13 (Gln140) BbZIP’s His177 is replaced by Gln. Gly182 of BbZIP is conserved in all plant ZIPs (Figure 1). The His177 and Gly182 are involved in the metal release from the metal-binding site of the BbZIP. Similarly, Glu211 and Gly212 are metal-binding residues in BbZIP. Glu211 of BbZIP is conserved in all plant ZIP proteins expect for AtZIP2 (Ala243), AtZIP11 (Ala215), OsZIP1 (Ala242), and OsZIP2 (Ala247) where Glu is replaced by Ala. Similarly, expect AtZIP2 (Ala224), AtZIP11 (Ala216), OsZIP1 (Ala243), and OsZIP2 (Ala248), other plants ZIP transporter proteins possess conserved Gly212 of BbZIP. Another interesting observation is that metal-binding site residues such as Asn178, Pro180, and Pro210 are conserved only with OsZIP13, OsZIP16, and ZmZIP6 of plants (Figure 1). Metal binding site residues Gln207 and Asp208 and metal-chelating residue Asp305 are not conserved with any other plant ZIPs. The Gln207 is replaced by His residue in all plant ZIPs (Figure 1). An invariable Ser106 is an important residue for guiding metals into the transport pathway of the BbZIP. In plants, Ser is completely replaced by His or Asp residue with His being a dominant residue. Overall, the metal binding and transport residues of OsZIP13, OsZIP16, and ZmZIP6 are more conserved with BbZIP compared to other plant ZIPs. These variations indicated that plant ZIP transporters may have partially overlapping but distinct metal transport mechanism compared to BbZIP as some of the residues involved in metal binding and transport are not conserved in plant ZIPs. We have also done homology modeling of plant ZIPs whose details are discussed below.

### Homology Modeling of Plant ZIP Transporters

**FIGURE 2:**
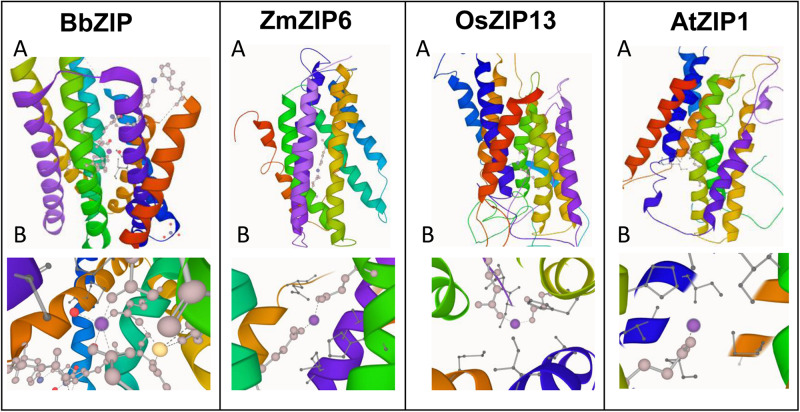
Homology modeling of plant ZIP transporters using *Bordetella bronchiseptica* ZIP (BbZIP) (PDB ID: 5TSA) as a template. The BbZIP structure was visualized using LiteMol viewer (https://www.litemol.org/Viewer/). Both whole model **(A)** and its Zn^2+^ binding site **(B)** is shown for each ZIP. The protein sequences of plant ZIPs were obtained from phytozome website (https://phytozome.jgi.doe.gov/pz/portal.html). The homology models of the plant ZIP proteins were generated using Modeler v9.22 (Eswar et al., 2007), which created 100 models for each target protein. The models were then ranked based on their modeler objective function value. The five best models were analyzed using MolProbity (http://molprobity.biochem.duke.edu/), an online tool. Ramachandran plots were generated for these five models and finally the best model was chosen based on the% residues in the favored and allowed regions and the number of outliers. The models were then visualized by LiteMol viewer.

In order to gain more insights to the structure of plant ZIP transporters, we have modeled some of the plant ZIP transporters by homology modeling using BbZIP (PDB Id: 5TSA) as a template with Zn^2+^ as a ligand. The models were then compared with the structure of BbZIP. In BbZIP, Zn^2+^ is coordinated by Glu181, Gln207, and Glu211 and water molecular at metal binding site as per our modeling (Figure 2; Zhang et al., 2017). The Zn^2+^ binding site is also covered by Glu276, His177, Met269, and Met99 which are involved in Zn transport (Figure 2). However, the plant ZIP transporters showed greater variation at Zn^2+^ binding site except for Glu181/211 (equivalent to BbZIP) based on the homology models (Figure 2 and Table 1). Zn^2+^ binding is coordinated by Glu141 and Glu170 in ZmZIP6, Glu252 and Asp190 in ZmZIP11, Glu173 and Glu240 in OsZIP16, Glu253 and Asn30 in OsZIP13. Even Zn^2+^ is co-ordinated only by a single Glu residue in AtZIP1 and AtZIP2 and not co-ordinated by any residues in AtZIP8 (Figure 2 and Table 1). The residues surrounding the Zn^2+^ binding site include Glu276, Met269, His177, and Met99 in BbZIP. These residues are partially conserved in some plant ZIPs and not at all conserved in other ZIPs modeled (Table 1). For e.g., His177 is conserved in OsZIP6 (His169) and ZmZIP6 (His166); shifting in the positioning of His is seen in AtZIP1 (His238), AtZIP2 (His239), OsZIP13 (His249), and ZmZIP11 (His278) as indicated in the alignment above (Figures 1, 2 and Table 1). Similar variation was seen for residues involved in Zn transport in the plant ZIPs analyzed (Table 1). It might be due to partially overlapping but distinct mechanism for binding and transport of Zn^2+^ by plant ZIPs which is also reflected in alignment (Supplementary Figure S1). This requires further studies to confirm the roles of functional residues especially by site directed mutagenesis with yeast mutants to gain more knowledge on residues involved in Zn^2+^ binding and transport in plant ZIPs. Also, the high-resolution crystal structure of plant ZIP is needed for understanding any specific Zn^2+^ transport mechanism in plants.

**TABLE 1:** Details on residues identified to be involved in Zn^2+^ transport through homology modeling using BbZIP as a template.

Name of the protein	UniProt Accession/Phytozome ID	Length (total amino acids)	Zn^2+^ coordinating residues	Zn^2+^ transporting residues
BbZIP	BB2405	309	Glu181; Glu211 Gln207	Glu276; Met 269 His177; Met 99
AtZIP1	O81123	355	Glu242	His238; Lys 89 Ile206; Met 312
AtZIP2	Q9LTH9	353	Glu216	His239; Leu77 Phe73
AtZIP8	Q8S3W4	347	–	Glu248; Asn251 Ile209; Ala247 Phe71
OsZIP13	LOC_Os07g12890 (Chen et al., 2008)	276	Asn30; Glu253	Ile59; Met321 His249; Ala85 Cys28
OsZIP16	LOC_Os08g01030 (Chen et al., 2008)	282	Glu173; Glu240	His169; Met48 Val144; Ala49
ZmZIP6	GRMZM2G050484_T01 (Mondal et al., 2013)	297	Glu141; Glu170	Glu237; Met51 His166; Ala47
ZmZIP11	GRMZM2G034551 (Mondal et al., 2013)	396	Glu282 Asp190	His278; Ile74 Lys198; Met159 Ala70

*Name of the protein, its UniProt Id/Phytozome Id, length of the protein, residues involved in Zn^2+^ binding are listed for each protein.*

### Phylogenetic Analysis of Plant ZIP Proteins

**FIGURE 3:**
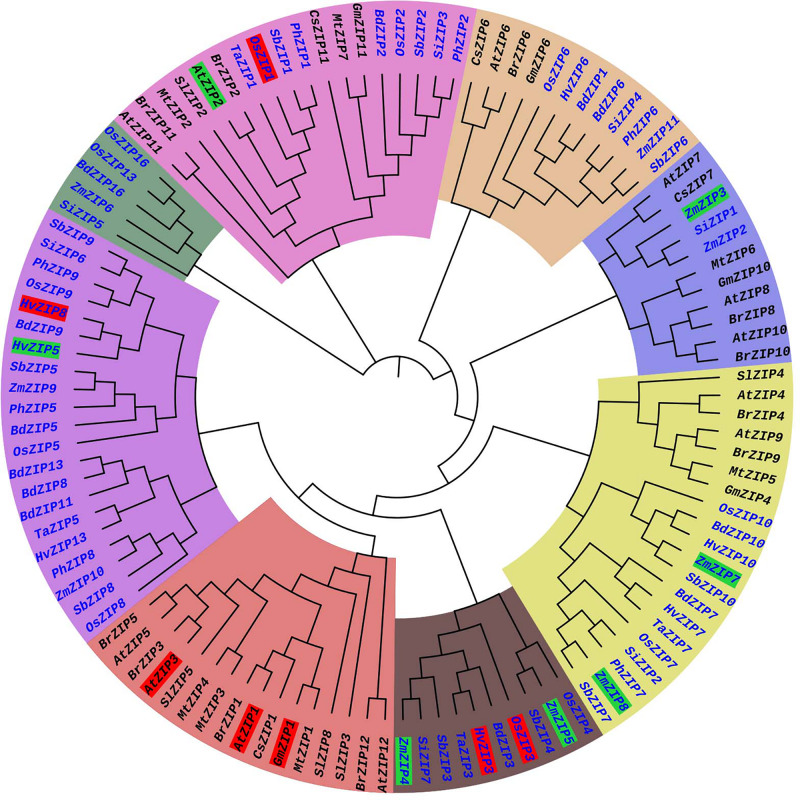
Phylogenetic tree of ZIP transporter family proteins of plants. The ZIP phylogenetic tree was constructed from 113 ZIP transporter protein sequences collected from 14 plant species. These included 8 monocot (Blue) and 6 dicot plants (Black). Each major cluster (MC1-MC8) is highlighted with different color. The low-affinity and high-affinity ZIP transporters are highlighted in red and green, respectively. The protein sequences of ZIP transporter family members were collected from Phytozome (www.phytozome.net) website. The phylogenetic tree was constructed by MEGA version 6 software with the maximum likelihood method based on the Jones-Taylor-Thornton matrix-based model. The bootstrap values are from 1000 replicates. The phylogeny tree was visualized by iTOL (https://itol.embl.de/).

The phylogenetic tree was constructed from 113 ZIP protein sequences collected from 14 plant species. These include eight monocot plants and six dicot plants (Supplementary File S1). The phylogenetic tree consisted of eight major clusters (MCs) (MC1–MC8) and sub-divided into 1–2 sub-clusters (SCs) and each SC was again divided into small clusters (Figure 3). More ZIP transporters are clustered in MC-8. The MC-8 had 21 ZIP transporters and it is followed by MC-5 (20 ZIPs) and MC-2 (18 ZIPs). The ZIP proteins of *Brassica rapa* (BrZIP1), *Cucumis sativus* (CsZIP1), *Medicago truncatula* (MtZIP1), and *Glycine max* (GmZIP1) were clustered with AtZIP1; BrZIP3 is closely clustered with AtZIP3 (Figure 3). The AtZIP1 and AtZIP3 proteins are clustered closely which are characterized as low-affinity transporters (Grotz et al., 1998). In *Arabidopsis*, *AtZIP1*, and *AtZIP3* are highly expressed in roots in response to Zn deficiency condition suggesting their role in Zn uptake from soil (Grotz et al., 1998). OsZIP1 and OsZIP3 are characterized as low-affinity Zn transporters in rice plants (Ramesh et al., 2003) and these are placed distinctly in the phylogenetic tree. The OsZIP1 is clustered with monocot ZIP transporters such as TaZIP1, *Panicum hallii* ZIP1 (PhZIP1) and SbZIP1. The expression analysis showed the *OsZIP1* gene is induced in roots under Zn starvation (Ramesh et al., 2003). Similarly, the OsZIP3 is closely clustered with HvZIP3, TaZIP3, BdZIP3, and SbZIP3. HvZIP3 was reported as a low-affinity transporter of barely (Pedas et al., 2009). Both *OsZIP3* and *HvZIP3* are highly expressed in both shoot and root under Zn deficient condition (Chen et al., 2008). AtZIP2 is a high-affinity transporter in *Arabidopsis* (Grotz et al., 1998), it is closely clustered with BrZIP2, SlZIP2 MtZIP2, TaZIP1, OsZIP1, PhZIP1, and SbZIP1 proteins. It is interesting to see that both low-affinity (OsZIP1) and high-affinity (AtZIP2) ZIPs are closely clustered. The monocot and dicot plant ZIPs are clustered in separate clusters. ZIP family member are mostly clustered together as per their numbers (Figure 3), for e.g., ZIP1s of *Arabidopsis*, mustard, barrel clover, soybean and sweet orange are closely clustered. The identification and characterization of the ZIP gene family are still lacking for many crops. More molecular and functional genomic studies will aid for the characterization of ZIP transporter in other plants in future.

## Regulation of ZIP Transporters in Plants

All living organisms need to maintain optimum concentrations of nutrients including Zn to sustain cellular functions (Shaul et al., 1999). Plants possess the homeostatic networks to maintain Zn levels in a narrow concentration to avoid either deficiency or toxicity. Zn homeostasis needs a complex network of cellular functions such as Zn uptake, accumulation, trafficking, sequestration, remobilization, and detoxification (Clemens, 2001; Krishna et al., 2017). ZIP transporters are regulated to provide a suitable quantity of Zn into all cell types and at all stages of development (Clemens, 2001). The Zn homeostasis equilibrium was considered for external and internal requirements of Zn (Claus and Chavarría-Krauser, 2012). Unfortunately, very little information is available on the regulation of plant ZIP transporters and mechanism of Zn homeostasis. Transport of divalent metal ions like Zn^2+^ by different transporters may pose some difficulty for high resolution studies to understand the role of plant ZIPs in Zn homeostasis. Expression levels of genes are increased for some ZIP transporters under Zn deficiency to facilitate the higher uptake of Zn from the soil. For e.g., in barley, *HvZIP3*, *HvZIP5*, and *HvZIP8* genes are highly induced in roots and are involved in Zn uptake under Zn deficient condition (Pedas et al., 2009).

Till date plant’s sensing and transferring the signal of Zn deficiency remains poorly understood. However, based on the available data, some transcription factors (TFs) are found to be essential for regulation of target genes and maintaining Zn homeostasis. Two TFs identified in model plant *Arabidopsis* are shown to be crucial in the adaptation response to Zn deficiency. The basic-region leucine zipper (bZIP) is a TF involved in the regulation of many physiological processes including abiotic and biotic stress responses (Corrêa et al., 2008). These TFs belong to the F group of bZIP and showed histidine-rich motifs at the basic N-terminal region (Nijhawan et al., 2008; Assunção et al., 2010). bZIP TFs are also involved in the up-regulation of ZIP transporters in *Arabidopsis* during Zn deficiency (Assunção et al., 2013). The TFs bZIP19 and bZIP23 are considered to be the essential regulators of candidate genes including ZIPs under Zn deficiency. In plants, bZIP19 and bZIP23 proteins exist as monomers under Zn sufficient condition. The Zn deficiency leads to the activation (binding) of bZIP9 and bZIP23 (dimerization) which induces the expression of ZIP genes. The bZIP19 and bZIP23 dimer bind to 10bp Zn-deficiency responsive elements (RTGTCGACAY) present in the promoter region of target genes (Assunção et al., 2010; Lilay et al., 2018). The histidine-rich motif is conserved among group F bZIP TFs (Jakoby et al., 2002; Castro et al., 2017) and suggested to play key role as a Zn-sensor (Assunção et al., 2013; Henriques et al., 2017). Totally, 33 *bZIP19* genes are identified and characterized in cereal crops using *in silico* approaches and histidine-rich motifs are found in most of these bZIP19 TFs (Henriques et al., 2017).

In *Arabidopsis*, the TFs AtbZIP19 and AtbZIP23 induce the expression of *AtZIP4* gene under Zn deficiency (Assunção et al., 2010). These TFs are also involved in the activation (up-regulation) of a specific subset of genes such as *AtZIP1, AtZIP2, AtZIP4, AtZIP5, AtZIP9, AtZIP10*, and *AtZIP12* (Assunção et al., 2010; Inaba et al., 2015; Lilay et al., 2018). Similarly, three bZIP TFs such as bZIP1, bZIP2, and bZIP3 were identified in common bean which are similar to *Arabidopsis* TFs bZIP19, bZIP23, and bZIP24 (Astudillo et al., 2013). In whole-genome transcriptome analysis, 3 bZIP TFs (*PvbZIP1*, *PvbZIP2*, and *PvbZIP3*) are found to be expressed under Zn deficiency condition in common bean (Astudillo-Reyes et al., 2015). But no information is available on target genes of these TFs. Similarly, seven bZIP TFs were identified in the wheat genome. Seven out of four TabZIP TFs such as TabZIPF1-7DL, TabZIPF3b-7BL, TabZIPF4-7AL, and TabZIPF4-7DL of wheat were used for functional complementation assay (Evens et al., 2017). The role of these four TabZIP TFs in the Zn homeostatic mechanism was determined by expression in the *Arabidopsis* double mutant line *bzip19-4 bzip23-2* under Zn deficient condition. The TabZIPF1-7DL and TabZIPF4-7AL could partially complement the double mutant line (*bzip19-4 bzip23-2*) under Zn-deficient condition. TabZIPF3b-7BL have slightly increased the growth of the double mutant line under Zn-deficient condition whereas TabZIPF4-7DL did not complement the double mutant lines (Evens et al., 2017). Similarly, seven bZIP TFs HvbZIP1, HvbZIP10, HvbZIP55, HvbZIP56, HvbZIP57, HvbZIP58, and HvbZIP62 were identified in barely (Nazri et al., 2017). Among these, the TFs HvbZIP56 and HvbZIP62 could restore the growth of *bzip19* and *bzip23* double mutant of *Arabidopsis* under Zn deficiency condition (Nazri et al., 2017). Recently, Lilay et al. (2020) identified and characterized three bZIP TFs in rice such as OsbZIP48, OsbZIP49, and OsbZIP50. The OsbZIP48 and OsbZIP50 complemented the *Arabidopsis* double mutant (*bzip19/bzip23*) under Zn deficient condition but the OsbZIP49 does not compliment the *Arabidopsis* double mutant (*bzip19/bzip23*) (Lilay et al., 2020).

In plants, concentrations of both the macro- and micro-nutrients influence each other through molecular cross-talks. Signals of P nutrition interact with those of the micronutrients like Zn and Fe. The TFs such as phosphate starvation response 1 (PHR1) (Rubio et al., 2001), ZAT6 (Devaiah et al., 2007b), WRKY75 (Devaiah et al., 2007a), and MYB62 (Devaiah et al., 2009) are involved in P deficiency response. The PHR1 acts as a positive regulator of inorganic phosphate (Pi) starvation responsive genes and PHR1 along with *Phosphate 1* (*PHO1*) are involved in Zn and Fe homeostasis in *Arabidopsis* (Khan et al., 2014; Rai et al., 2015). Zn deficiency also induced the expression of P stress-responsive genes through *PHR1* and increased the uptake of Pi (Bouain et al., 2014). PHR1 seems to be a positive regulator of the ZIP transporters (AtZIP2 and AtZIP4) under Pi deficiency in *Arabidopsis* (Briat et al., 2015). The cross-talk of signals between P, Zn, and Fe were highlighted by many articles (Briat et al., 2015; Xie et al., 2019). Only a very little research is done on the characterization Zn responsive and ZIP related TFs in crops and most of the reports are also confined only to plants like *Arabidopsis*. Therefore, more studies are needed on the identification and characterization of Zn responsive TFs in crops. This could help to understand the molecular mechanism of Zn deficiency tolerance and may aid in developing Zn deficiency tolerant crops.

## ZIP Family Transporters Identified in Various Crops

**FIGURE 4:**
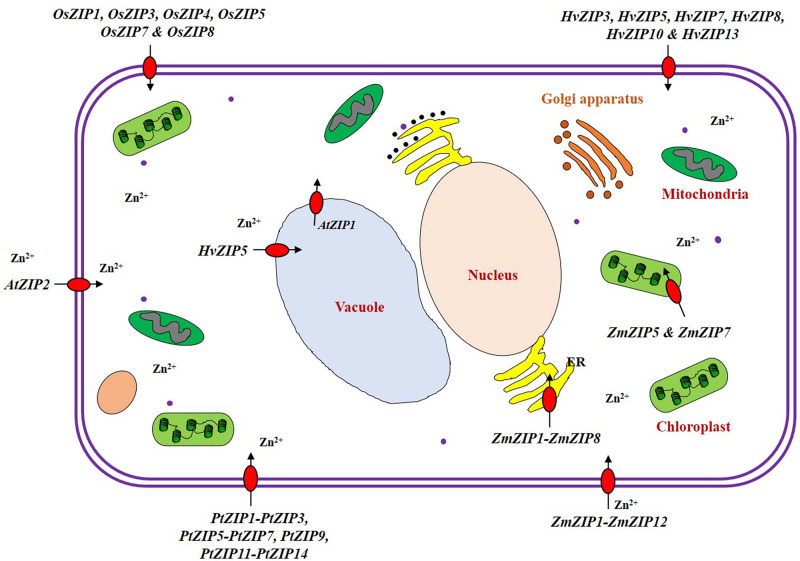
Localization of ZIP transporter family proteins in the plant cell. The ZIP transporter family proteins are actively involved in uptake, transport, detoxification and homeostasis of Zn within plant cells (Li et al., 2013). Under low Zn condition, many ZIP transporters (ZIP1-ZIP12) are expressed and are localized in plasma membrane of the cell in rice, (Ramesh et al., 2003; Ishimaru et al., 2005; Yang et al., 2009; Lee et al., 2010a, b) hardy orange (Fu et al., 2017), maize (Li et al., 2013; Mondal et al., 2013), *Arabidopsis* (Milner et al., 2013), and barley (Pedas et al., 2009; Tiong et al., 2015). Some of the ZIP transporters are also localized to the membranes of intracellular organelles such as chloroplast [ZmZIP5 and ZmZIP7 (Li et al., 2013)], vacuole [HvZIP5 (Tiong et al., 2015) and AtZIP1 (Milner et al., 2013)], and endoplasmic reticulum [ZmZIP1-ZmZIP8 (Li et al., 2013)].

We have enlisted the ZIP family members identified so far in crops (Supplementary Table S1), and the details are discussed below. ZIP family transporter genes have been identified and functions characterized majorly in model plants like *Arabidopsis* and rice. Identification and characterization of the ZIP family genes are still lacking for many crops. Expression levels of ZIP family genes showed dynamic pattern in crops. For e.g., a few ZIP transporters are expressed only under deplete Zn conditions, so their expression is declined within 2 h when Zn is added to the medium (Van de Mortel et al., 2006). The ZIP transporters are localized to plasma membrane and membranes of many intra-cellular organelles (Figure 4). It is proved that the plant ZIP transporters are involved in Zn uptake at the cellular level when tested in yeast complementation test (Fu et al., 2017). In model plant *Arabidopsis*, the ZIP transporters such as AtZIP1, AtZIP2, AtZIP3, and AtZIP4 have been functionally characterized to be the transporters with different affinities (Grotz et al., 1998; Mäser et al., 2001; Assunção et al., 2010). Evidence also shows the direct involvement of ZIP transporter in enhancing Zn accumulation at edible parts of the plants (Ramegowda et al., 2013; Gaitán-Solís et al., 2015). Rice has been the only cereal model crop whose ZIP family transporters have been studied to a reasonable extend when compared to other crops. So, the details on rice ZIP and IRT transporters are discussed in detail in the following section which could serve as a model to study the ZIP and IRT transporters of other crops especially cereals. We also discussed the details on ZIP family transporters identified in other crops.

### Rice

**FIGURE 5:**
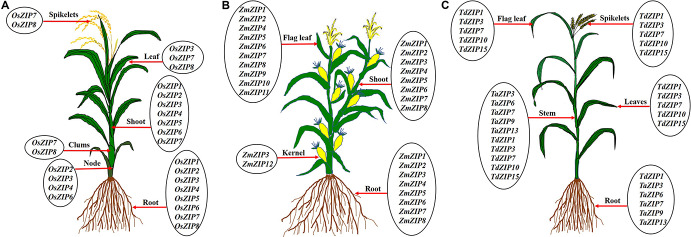
Expression of ZIP transporter genes in different parts of rice **(A)**, maize **(B)**, and wheat **(C)**. The ZIP transporters are expressed in root, nodes, clumps, shoot/stem, leaf, flag leaf, kernel, and spikelets under Zn deficient levels. Under low Zn condition, root tissues show the expression of ZIP genes in rice (Ramesh et al., 2003; Ishimaru et al., 2005; Chen et al., 2008; Yang et al., 2009; Suzuki et al., 2012), maize (Li et al., 2013; Mondal et al., 2013) and wheat (Durmaz et al., 2011; Evens et al., 2017; Deshpande et al., 2018), clumps in rice (Yang et al., 2009), shoot/stem in rice (Ramesh et al., 2003; Ishimaru et al., 2005; Chen et al., 2008; Yang et al., 2009; Suzuki et al., 2012), maize (Li et al., 2013; Mondal et al., 2013), and wheat (Durmaz et al., 2011; Evens et al., 2017). Leaf tissue also showed the over expression of ZIP genes under low Zn soil in rice (Ramesh et al., 2003; Ishimaru et al., 2005; Chen et al., 2008) and wheat (Evens et al., 2017; Deshpande et al., 2018), flag leaf tissues in maize (Mondal et al., 2013) and wheat (Deshpande et al., 2018), kernel tissue in maize (Li et al., 2013) and spikelets tissue in rice (Yang et al., 2009) and wheat (Deshpande et al., 2018).

Rice is a staple food for more than 560 million people in the world; it is one of the highly sensitive crops to Zn deficiency (Krithika and Balachandar, 2016). Many works were conducted on rice to understand the Zn transport mechanism and rice seems to be a good model for other crops to study the Zn transport. Totally 16 ZIP transporter members were identified in rice (Ramesh et al., 2003; Ishimaru et al., 2005; Chen et al., 2008). Some *OsZIP* genes are expressed in both roots and shoots and others are expressed in whole parts of the plant such as root, culms, leaves, and spikelets under Zn deficiency as reported by different authors (Figure 5A). In rice, several ZIPs such as OsZIP1, OsZIP3, OsZIP4, OsZIP5, OsZIP7, and OsZIP8 were reported to be responsible for Zn uptake from soil, translocation from root to shoot as well as for grain filling (Ramesh et al., 2003; Ishimaru et al., 2005, 2007; Lee et al., 2010b; Meng et al., 2018). In rice, understanding the molecular mechanisms of Zn transport is important for improving the Zn content in the edible part. Ishimaru et al. (2011) highlighted the mechanism of Zn uptake and translocation in rice. The OsZIP1 and OsZIP3 seem to be important for Zn uptake from the soil, OsZIP4, OsZIP5, and OsZIP8 for root to shoot translocation, while OsZIP4 and OsZIP8 played a role in grain filling (Bashir et al., 2012). The expression levels of *OsZIP3* and *OsZIP4* are higher in the roots of Zn-efficient genotype (IR8192) than those of in-efficient genotype (Erjiufeng) suggesting that these genes could contribute to high Zn efficiency (Chen et al., 2008).

Under Zn deficient condition, *OsZIP1*, *OsZIP3*, and *OsZIP4* are up-regulated in the roots and *OsZIP4* over-expressed in the shoot of both genotypes (Chen et al., 2008). These transporters are localized to the vascular bundles in shoots and in the vascular bundles and epidermal cells in roots of rice (Ramesh et al., 2003; Ishimaru et al., 2006). The expression analysis shows that *OsZIP4* is highly expressed under Zn deficient conditions in roots and shoots in rice (Ishimaru et al., 2005). It is expressed in the meristem of the roots and shoots, and also in vascular bundles of the roots and shoots under Zn deficient condition (Ishimaru et al., 2005; Ishimaru et al., 2011). The expression of *OsZIP1* gene is seen only in root tissues under Zn starvation, but *OsZIP3* is expressed in both roots and shoots under both Zn sufficient and deficient conditions (Ramesh et al., 2003). *OsZIP7* and *OsZIP8* are expressed in roots and shoots under Zn deficient condition (Yang et al., 2009; Tan et al., 2019). The *OsZIP3* and *OsZIP4* are highly expressed in the nodal regions under Zn deficiency condition (Sasaki et al., 2015). *OsZIP1* is expressed in the epidermis and vascular tissues of roots and leaves of rice (Ramesh et al., 2003; Bashir et al., 2012). The OsZIP4, OsZIP5, and OsZIP8 transporters are localized to plasma membrane and are involved in Zn influx (Ishimaru et al., 2005; Lee et al., 2010a, b). The *OsZIP3* gene was expressed in the nodal region which is responsible for unloading Zn from the xylem (Sasaki et al., 2015). OsZIP7 is located in the parenchyma cells of vascular bundles in nodal region, and in the stele in the roots of rice (Tan et al., 2019). OsZIPs might be actively involved in the uptake and transport of Zn in rice. Similarly, Ishimaru et al. (2011) suggest that OsZIP4 may be responsible for Zn translocation to aerial parts.

The OsIRTs are similar to OsZIP metal transporters; these are the members of ZIP family transporters (Guerinot, 2000). Especially OsZIP4, OsZIP5, OsZIP6, and OsZIP7 showed sequence similarity to OsIRT1 (Ishimaru et al., 2005). The expression analysis revealed that *OsIRT1* and *OsIRT2* are mainly expressed in roots of rice under low Fe conditions (Bughio et al., 2002; Ishimaru et al., 2006; Lee and An, 2009). The expression level *OsIRT1* was much higher than *OsIRT2* in the Fe deficient root of rice (Ishimaru et al., 2006). *OsIRT1* and *OsIRT2* have been cloned and characterized in yeast (Bughio et al., 2002; Ishimaru et al., 2006). The OsIRT1 and OsIRT2 proteins localize to the plasma membrane and have been shown to complement the growth defect of a yeast Fe uptake mutant, confirming that they are functional Fe^2+^ transporters under low Fe condition (Bughio et al., 2002; Ishimaru et al., 2006). The OsIRT transporters also capable of transporting other divalent metal cations such as Cd^2+^, Zn^2+^, Cu^2+^, and Mn^2+^ (Bughio et al., 2002; Nakanishi et al., 2006). Cellular and sub-cellular metal homeostasis is very crucial for maintaining the optimal metabolic process and cellular functioning (Bashir et al., 2016). Therefore, identification and characterization of IRT transporters are essential for understanding the mechanism of Fe homeostasis in rice and other plants.

The information available on Zn transporters of rice plants are high when compared to other crops such as maize, wheat, barley, foxtail millet, orange, and common bean, etc. This large information is helpful for understanding the expression pattern of Zn transporters, their localization and metal homeostasis in other crops. Apart from these reports we need further studies on the biofortification of rice with Zn in order to increase the yield and quality of rice as it helps to improve the nutritional status of humans.

### Maize

Maize is one of the most widely cultivated and important cereal crops for human and animal food (Shiferaw et al., 2011). Zn is the most common limiting micronutrient in maize yield worldwide (Alloway, 2009). The maize genome has been sequenced and assembled (Palmer et al., 2003). Li et al. (2013) identified eight ZIP transporters (*ZmZIP1*–*ZmZIP8*) in maize genome. All eight ZmZIP proteins are localized to the plasma membrane (Li et al., 2013). The expression analysis indicated that *ZmZIP3*, *ZmZIP4*, *ZmZIP5*, *ZmZIP7*, and *ZmZIP*8 are sensitive to Zn status during the seedlings stage of maize (Li et al., 2013). Under Zn deficient condition, *ZmZIP5* and *ZmZIP8* are induced in shoot and *ZmZIP3* is up-regulated in both root and shoot (Li et al., 2013). The expression of *ZmZIP4, ZmZIP5, ZmZIP7*, and *ZmZIP8* decreased in shoots and *ZmZIP3* is down-regulated in roots of maize under excess Zn supply (Li et al., 2013). Similarly, 12 *ZmZIP* genes (*ZmZIP1*–*ZmZIP12*) were identified by Mondal et al. (2014) in maize. Ten *ZmZIP* genes (*ZmZIP1, ZmZIP2*, and *ZmZIP4–ZmZIP11*) are tissue-specific and are highly expressed in flag leaf except *ZmZIP3* and *ZmZIP12*, under Zn deficient condition (Mondal et al., 2013). The *ZmZIP2*, *ZmZIP5*, *ZmZIP6*, *ZmZIP8*, and *ZmZIP11* are expressed in the kernel under Zn deficient condition (Mondal et al., 2013). ZmZIP4, ZmZIP5, ZmZIP7, and ZmZIP9 proteins are located in cytoplasm and chloroplast (Mondal et al., 2013; Figure 4). It is well known that some ZIP transporters are involved in Zn biofortification in other crops. For example, the OsZIP4 and OsZIP8 are actively involved in the grain filling of Zn in rice (Ishimaru et al., 2007; Lee et al., 2010b). The *ZmZIP5* and *ZmZIP11* are highly expressed in the flag leaf, which might play a vital role in the mobilization of Zn from flag leaf to developing kernel for the accumulation of the large amount of Zn in the kernel (Mondal et al., 2013; Figure 5B). These ZmZIPs (ZmZIP5 and ZmZIP11) might contribute to the biofortification of maize with Zn to improve the quality (Mondal et al., 2013). Since maize is highly sensitive to Zn deficiency, more high resolution studies are needed to improve the quality of maize grown under low Zn soils.

### Wheat

Wheat is a major cereal crop for whole world and it provides about one-half of humans’ food calories and a large part of their nutrient requirements (Shiferaw et al., 2013). Complete assembly of the hexaploid bread wheat genome is available now (Zimin et al., 2017). Previously, very little was known about ZIP family transporters except for TdZIP1 from wild emmer wheat (*Triticum turgidum* ssp. *dicoccoides*), a Zn transporter with higher expression under Zn deficiency (Durmaz et al., 2011). Recently, 14 *TaZIP* genes were identified in bread wheat (*Triticum aestivum*) (Evens et al., 2017). Out of 14 ZIPs, five (*TaZIP3, TaZIP5, TaZIP6, TaZIP7*, and *TaZIP13*) were analyzed for the expression level in shoot and root under Zn starvation. In shoot, expression of all five *TaZIPs* increased under low Zn conditions but the timing varied between individual genes. In roots, *TaZIP3*, *TaZIP5*, *TaZIP7*, and *TaZIP13* showed increased expression under Zn starvation with expression of only *TaZIP6* gene remains fairly stable (Evens et al., 2017). Similarly, Deshpande et al. (2018) analyzed the relative expression of five selective ZIP genes in durum wheat (*Triticum durum*) genotypes UC 1114 and MACS 3125 under three different foliar application of Zn (Deshpande et al., 2018). In flag leaves, expression level of three *TdZIP* genes (*TdZIP1, TdZIP3*, and *TdZIP7*) decreased and two genes (*TdZIP10* and *TdZIP15*) increased during grain development (Deshpande et al., 2018). Time-dependent expression patterns of these *ZIP* transporters revealed that the expression pattern varies with tissues viz., flag leaves, non-flag leaves, stem, and spike (Deshpande et al., 2018). These studies revealed that most of the *TaZIP* transporter genes are expressed in all parts and are involved in Zn uptake and translocation under Zn starvation (Figure 5C). Till now, totally 16 *ZIP* genes are identified in the wheat genome and only a few works have been carried out on the expression analysis with these when compared to rice. No reports are available on localization and functional characterization of TaZIP transporters which may help to understand further on the Zn transport mechanism.

### Barley

Barley is a nutritionally and economically important cereal crop. Till date, only a few *ZIP* family genes have been identified and characterized in barely. Pedas et al. (2009) reported that *HvZIP3*, *HvZIP5*, and *HvZIP8* are induced in root tissues under Zn deficient condition. These three HvZIP transporters might be involved in Zn uptake under low Zn conditions. *HvZIP7* is highly induced in the vascular tissues of roots and leaves under Zn deficiency condition and its protein is localized to plasma membrane (Tiong et al., 2014). In another report, 13 *HvZIP* genes were identified and their tissue-specific expression was also determined under Zn deficiency condition. Out of 13 *HvZIP* genes, six (*HvZIP3*, *HvZIP5*, *HvZIP7*, *HvZIP8*, *HvZIP10*, and *HvZIP13*) were highly induced in Zn deficient barely (Tiong et al., 2015). The expression levels of these six *HvZIP* genes are significantly increased (3-fold) in roots under Zn deficient condition (0.005 μM Zn) compared to Zn sufficient condition (0.5 μM Zn) (Tiong et al., 2015). All the six HvZIP proteins are localized to plasma membrane (Tiong et al., 2015). The *HvZIP2*, *HvZIP3*, *HvZIP5*, *HvZIP7, HvZIP8*, *HvZIP10*, and *HvZIP13* are also induced in shoot of barley under Zn deficient condition. Recently, five *HvZIP* family genes such as *HvZIP3*, *HvZIP7*, *HvZIP8*, *HvZIP9*, and *HvZIP13* were screened for their expression under the arbuscular mycorrhizal fungi (AMF) medium with low and high Zn treatments (Watts-Williams and Cavagnaro, 2018). Under low Zn condition, the *HvZIP* genes were up-regulated in the root of barley in the non-AMF condition. But, the activity of AMF has altered the expression of *HvZIP* family genes. The *HvZIP13* gene was significantly overexpressed in the mycorrhizal plant than non-mycorrhizal plants under low Zn condition. Two *HvZIP* genes (*HvZIP3* and *HvZIP8*) are down-regulated in mycorrhizal roots at low Zn soil (Watts-Williams and Cavagnaro, 2018). Therefore, ZIP genes may be directly or indirectly involved in the transport of Zn between the AMF and plant under low Zn conditions. Apart from this, no other information is available on the effect of AMF on the ZIP gene expression in other crops. AMF has the ability to improve the nutrition of the host plant through increased uptake of soil nutrients, especially immobile nutrients such as P, K, Zn, Fe, and Cu (Pellegrino and Bedini, 2014; Pellegrino et al., 2015; Watts-Williams and Cavagnaro, 2018). Kaiser et al. (2015) reported that the AMF (*Glomus contrictus* or *Glomus fasciculatus*) enhances the Zn up-take in barely. Further studies are needed to understand the molecular mechanism of AMF-mediated Zn transport and signals and TFs involved in this symbiotic Zn uptake.

### Foxtail Millet

Foxtail millet is one of the food security minor cereal crops in low input regions. It is a domesticated diploid C4 crop having a small genome (∼515 Mb) with short life cycle (Doust et al., 2009). So, it is considered as a model crop for genetic and genomic studies of monocots (Lata et al., 2013; Ceasar et al., 2014). The whole-genome sequence is available for two different genotypes of foxtail millet (Bennetzen et al., 2012; Zhang et al., 2012). But, the information about ZIP genes is limited. Alagarasan et al. (2017) identified seven *SiZIP* genes (*SiZIP1*–*SiZIP7*) in foxtail millet. The expression pattern of seven *SiZIP* genes was analyzed in root, leaf, stem and spica tissues of foxtail millet grown under drought stress without any additional nutrient supplementation. All seven *SiZIP* genes were induced in all four tissues (root, leaf, stem, and spica) with various levels of expression. The *SiZIP2*, *SiZIP3*, *SiZIP4*, and *SiZIP5* showed relatively higher expression and low level of expression was observed with *SiZIP6*, in all tissues. *SiZIP1* gene is moderately expressed in root, shoot and spica and very least expression was seen in leaf (Alagarasan et al., 2017). The highly induced *SiZIP* genes (*SiZIP2*, *SiZIP3*, *SiZIP4*, and *SiZIP5*) could be used for the bio-fortification process for the enrichment of Zn into the seeds of foxtail millet. The functional characterization of ZIP genes in foxtail millet could aid to improve the Zn uptake in foxtail millet and other minor millets.

### Orange

Orange is an important fruit crop with 60 million metric tonnes of annual production worldwide and has high nutritional values, vitamins and other nutrients (Etebu and Nwauzoma, 2014). Till date, orange is the only fruit crop in which ZIP transporters are identified. In navel orange (*Citrus sinensis*), four *ZIP* genes such as *CsZIP1*, *CsZIP2, CsZIP3*, and *CsZIP4* were identified and expression analysis show that *CsZIP3* and *CsZIP4* are highly expressed in mild, moderate and severely affected Zn deficient leaf. *CsZIP1* gene is down-regulated under mild Zn deficient leaf and up-regulated in moderate and severely Zn depleted leaf. There is no change in the expression of *CsZIP2* in all tissues when compared with control (Fei et al., 2016). Recently, 13 *ZIP* (*PtZIP1–PtZIP3* and *PtZIP5–PtZIP14*) genes were identified in trifoliate orange (*Poncirus trifoliata*), another variety of orange fruit crop. *PtZIP1*, *PtZIP2*, *PtZIP3*, *PtZIP5*, *PtZIP6*, and *PtZIP9* are highly induced in roots, whereas *PtZIP1*, *PtZIP2*, *PtZIP5*, *PtZIP6*, and *PtZIP7* are highly expressed in leaves, under Zn-deficient condition (Fu et al., 2017). In future, functional characterization of ZIP genes in orange is very important to improve the Zn content in the edible parts of orange. Therefore, it will help to reduce the Zn deficiency problems in humans. So, more studies are needed to improve the quality of orange in terms of biofortification.

### Common Bean

Common bean is an important food crop for human as it contains a rich source of nutrients (Gepts et al., 2008), especially a source of dietary Zn (Astudillo-Reyes et al., 2015). Totally 19 *PvZIP* genes (*PvZIP1*–*PvZIP19*) were identified in common bean. But, only seven *PvZIP* genes such as *PvZIP2*, *PvZIP6*, *PvZIP7*, *PvZIP12*, *PvZIP13*, *PvZIP16*, and *PvZIP18* were analyzed for their expression dynamics. The selection of *PvZIP* genes is based on their location in the genome relative to the presence of QTLs for seed Zn content (Astudillo et al., 2013). *PvZIP12*, *PvZIP13*, and *PvZIP16* genes are expressed in root, leaf and pod of the common bean under Zn deficient condition (Astudillo et al., 2013). Other *PvZIP* genes are not expressed in any tissue types in Zn deficient condition. The *PvZIP12* is highly expressed in leaves at vegetative stage and *PvZIP13* is highly expressed in leaves at flowering stage. Hence, the *PvZIP12* gene could be involved in mobilizing the Zn to seeds of bean. The identification and characterization of the candidate genes related to *PvZIP* transporters may help to improve Zn uptake and nutrient content of seeds.

## Functional Characterization of ZIP Transporters in Crops

Till date, only a little information is available on functional characterization of ZIP transporters in crops (Milner et al., 2013). Mostly, the investigations on the functions of plant ZIP transporters have been conducted by yeast complementation assays which revealed that ZIP transporters are capable of transporting various divalent cations (Guerinot, 2000). For functional characterization, Zn sensitive yeast mutant (*zrt1/zrt2*) which is defective in both the ZRT1 high−affinity and the ZRT2 low−affinity uptake transporters and is susceptible to Zn deficient conditions, was employed (Zhao and Eide, 1996a, b). In most of these studies, genes of model plant *A. thaliana* are used. The *AtZIP* genes such as *AtZIP1*, *AtZIP2*, and *AtZIP3* enabled Zn transport based on the yeast complementation assay (Eide, 1998; Grotz et al., 1998; Guerinot, 2000). In another study, six ZIP genes such as *AtZIP1*, *AtZIP2*, *AtZIP3*, *AtZIP7*, *AtZIP11*, and *AtZIP12*, are able to complement the *zrt1/zrt*2Δ yeast mutant fully or partially under Zn deficient conditions (Milner et al., 2013). Recently, Fu et al. (2017) characterized the Zn transport specificities of the *PtZIP* genes and *PtZIP1*, *PtZIP2*, *PtZIP3*, and *PtZIP12* are also able to complement *zrt1zrt2* mutant (Fu et al., 2017).

In plants, the high-affinity ZIP transporter system was highly active under low Zn conditions. Based on a yeast complementation assay, the affinity values of AtZIP1 (*K*_m_ = 13 μM) and AtZIP3 (*K*_m_ = 14 μM) confirmed to be low-affinity transporters and AtZIP2 (*K*_m_ = 2 μM) as high-affinity transporter (Grotz et al., 1998). In rice, OsZIP1 (*K*_m_ = 16.3 μM) and OsZIP3 (*K*_m_ = 18.5 μM) are characterized as low-affinity Zn transporters (Ramesh et al., 2003). The GmZIP1 of soybean was identified to be a low-affinity transporter (*K*_m_ = 13.8 μM) (Moreau et al., 2002). The high affinity transporters ZmZIP3, ZmZIP4, ZmZIP5, ZmZIP7, and ZmZIP8 were identified in maize (Li et al., 2013; Mondal et al., 2013). Similarly, Pedas et al. (2009) found that HvZIP3 and HvZIP8 are low-affinity transporters and HvZIP5 is a high-affinity Zn transporter in barely based on yeast complementation assay. However, additional kinetic assay is essential for confirming this hypothesis. Till now, very little information is available on the affinities of ZIP transporters in crops (Table 2). Further, heterologous expression of plant ZIP transporters in yeast may not replicate the same function and may yield false results due to being a completely different system and lack of key signals involved in the regulation of Zn concentration. Testing the function of plant ZIPs in the same plant by knock-out studies may yield confident results. To this end, newly adopted genome editing tool CRISPR/Cas9 may help for the efficient generation of single, double and multiple mutants for plant ZIPs to test their function in the same system. Characterization of other ZIP transporters could help to understand the crucial roles of ZIP transporter family genes in crops.

**TABLE 2:** Details on the ZIP family transporter genes identified in plants.

Name of the plant	Common name	Name of ZIP genes	Method of characterization	Affinity		References
				Low-affinity	High-affinity	
*Arabidopsis thaliana*	*Arabidopsis*	*AtZIP1*-*AtZIP4*	Yeast complementation assay	*AtZIP1* (*K*m = 13 μM) and *AtZIP3* (*K*m = 14 μM)	*AtZIP2* (*K*m = 2 μM)	Grotz et al., 1998
*Poncirus trifoliate*	Trifoliate orange	*PtZIP1*-*PtZIP3*, *PtZIP5*-*PtZIP7*, *PtZIP9* and *PtZIP10*- *PtZIP14*	qRT-PCR and yeast complementation assay	–	–	Fu et al., 2017
*Setaria italica*	Foxtail millet	*SiZIP1*-*SiZIP7*	RT-PCR	–	–	Alagarasan et al., 2017
*Glycine max*	Soybean	*GmZIP1*, *GmZIP4, GmZIP4, GmZIP6, GmZIP10* and *GmZIP11*	Yeast complementation assay	*GmZIP1* (*K*m = 13.8 μM)	–	Moreau et al., 2002
*Oryza sativa*	Rice	*OsZIP1- OsZIP16*	qRT-PCR complementation assay	*OsZIP1* (*K*m = 16.3 μM) *and OsZIP3* (*K*m = 18.5 μM)	–	Ramesh et al., 2003; Ishimaru et al., 2005; Chen et al., 2008; Yang et al., 2009
*Phaseolus vulgaris*	Common bean	*PvZIP1- PvZIP18*	qRT-PCR	–	–	Astudillo et al., 2013
*Zea mays*	Maize	*ZmZIP1- ZmZIP12*	qRT-PCR and yeast complementation assay	–	*ZmZIP3*, *ZmZIP4*, *ZmZIP5*, *ZmZIP7* and *ZmZIP8*	Li et al., 2013; Mondal et al., 2013
*Triticum aestivum*	Wheat	*TaZIP1-TaZIP3, TaZIP5-TaZIP7, TaZIP9-TaZIP11, TaZIP13, TaZIP14* and *TaZIP16*	qRT-PCR and yeast complementation assay	–	–	Evens et al., 2017
*Citrus sinensis*	Navel orange	*CsZIP1-CsZIP4*	qRT-PCR	–	–	Fei et al., 2016
*Hordeum vulgare*	Barley	*HvZIP1-HvZIP3, HvZIP5- HvZIP8, HvZIP10, HvZIP11, HvZIP13, HvZIP14* and *HvZIP16*	qRT-PCR and yeast complementation assay	*HvZIP3* and *HvZIP8*	*HvZIP5*	Pedas et al., 2009; Tiong et al., 2015
*Triticum durum*	Durum wheat	*TdZIP1, TdZIP3, TdZIP7 TdZIP10* and *TdZIP15*	qRT-PCR	–	–	Deshpande et al., 2018
*Triticum dicoccoides*	Emmer wheat	*TdZIP1*	qRT-PCR and yeast complementation assay	–	–	Durmaz et al., 2011

*The details on name of the plant, name of ZIP genes, affinity of ZIP transporter and mode of characterization are provided.*

## Manipulation of Expression Levels of ZIP Genes Through Transgenic Modification

The plant ZIP genes were over expressed through transgenic modification in some studies. Till now, only a few genes such as *AtZIP1*, *OsZIP1*, *ZmZIP3*, *OsZIP4, OsZIP5*, and *HvZIP7* were used for over-expression analysis in crops (Table 3). For example, *OsZIP1* gene was transferred into finger millet using *pGreen0179* vector under the control of *Bx17* promoter through *Agrobacterium-*mediated transformation; the transgenic plants showed significantly improved accumulation of Zn in seeds (Ramegowda et al., 2013). Similarly, over-expression of *AtZIP1* gene in cassava showed an increase of 25% Zn content in the edible part of the plant (Gaitán-Solís et al., 2015). Over expression of *OsZIP4, OsZIP5*, and *OsZIP8* in transgenic rice decreased the Zn content in shoot and seed; but significantly increased the root Zn content (Ishimaru et al., 2007; Lee et al., 2010a, b). It clearly revealed that these OsZIP transporters play a crucial role in Zn uptake from the soil. Over-expression of *OsZIP4* in rice plants showed the reduction of plant growth under Zn deficient conditions (Ishimaru et al., 2007). Approximately, 50% reduction observed in plant height and root length in *OsZIP4* over-expressing transgenic rice plants (Ishimaru et al., 2007). At the flowering stage, *OsZIP5* and *OsZIP8* over-expressing transgenic rice plants are shorter and had fewer tillers (Lee et al., 2010a, b). Also, these transgenic plants produce very fewer grains (Lee et al., 2010a, b). Similarly, over-expression of *OsIRT1* in rice altered the plant architecture and increased the Fe and Zn contents in mature seeds and vegetative parts of the plant under Zn deficient condition (Lee and An, 2009). These studies indicate that the ZIP transporters are responsible for improving the nutritional quality in crops. So, more effort is needed to identify the specific function of all ZIP genes in crops and it could help to develop transgenic plants related to biofortification of edible parts with Zn/Fe in crops.

**TABLE 3:** Details on genetic manipulation of ZIP transporter genes reported in various plants.

Name of the ZIP gene	Source	Host	Name of vector used	Mode of transformation	Observation	References
*ZmZIP3*	*Zea mays*	*Arabidopsis thaliana*	pBI121	*Agrobacterium tumefaciens*	Improved Zn accumulation in the roots	Li et al., 2015
*AtZIP1*	*Arabidopsis thaliana*	*Manihot esculenta*	pCAMBIA2301	*Agrobacterium tumefaciens*	Higher Zn concentrations in the edible portion	Gaitán-Solís et al., 2015
*HvZIP7*	*Hordeum vulgare*	*Hordeum vulgare*	pMDC32	*Agrobacterium tumefaciens*	Increases root to shoot translocation of Zn	Tiong et al., 2014
*OsZIP1*	*Oryza sativa*	*Eleusine coracana* and *Nicotiana tabacum*	pGreen0179	*Agrobacterium tumefaciens*	Enhanced Zn concentration in seed and plant tissue	Ramegowda et al., 2013
*OsZIP5*	*Oryza sativa*	*Oryza sativa*	pCAMBIA1302	*Agrobacterium tumefaciens*	Decreased Zn concentration in shoot and increased in root	Lee et al., 2010a
*OsZIP8*	*Oryza sativa*	*Oryza sativa*	pCAMBIA1302	*Agrobacterium tumefaciens*	Decreased Zn concentration in shoot and seed; increased in root	Lee et al., 2010b
*OsZIP4*	*Oryza sativa*	Oryza sativa	pIG121Hm	*Agrobacterium tumefaciens*	Zn distribution	Ishimaru et al., 2007
*AtZIP1*	*Arabidopsis thaliana*	*Hordeum vulgare*	pWVec8	*Agrobacterium tumefaciens*	Increased Zn content in seed	Ramesh et al., 2004

*The name of the plant, vectors and the role of ZIP genes are mentioned.*

## Conclusion and Future Prospects

Zinc is not only essential for plant growth but also crucial for human health. It is estimated that nearly 50% of the world’s population is at the risk of Zn deficiency problems (Pedas and Husted, 2009). In humans, Zn deficiency impairs growth and development, affects the nervous system, reduces immunity and can cause death (Ekweagwu et al., 2008; Menguer et al., 2018). Researchers need to pay more attention to the biofortification of food crops with priority to Zn. The ZIP transporters play a crucial role in biofortification with Zn. In soil, Zn deficiency problem is usually tackled by adding Zn containing fertilizers. But, it is only a temporary solution. Also, the subsistence farmers cannot afford to buy Zn fertilizers all the time due to high market price. Identification of candidate genes related to ZIP transporters, their characterization and tapping this information to develop crops via transgenic and/or the marker-assisted selection may help for developing Zn efficient crops. Genetic modification of crops with ZIP genes is also helpful in improving the crops related to Zn use efficiency. Therefore, the structural and functional insight of plant ZIP transporter are essential. The bioinformatics study showed that the plant ZIP transporters may have partially overlapping but distinct metal transport mechanism compared to BbZIP as some of the residues involved in metal binding and transport are not conserved in plant ZIPs. The plant ZIP transporters showed greater variation at the Zn^2+^ binding site when compared to BbZIP. The phylogenetic analysis of 113 plant ZIP proteins from 14 plant species revealed that the ZIP family members are mainly clustered together as per their numbers. Monocot and dicot plant ZIPs are clustered in separate clusters and both low-affinity and high-affinity ZIPs are closely clustered. These findings would be a valuable theoretical knowledge for future studies in terms of understanding the gene, protein, functional residues of Zn transporters in crops and might be helpful to overcome the problems associated with Zn deficiency. Many ZIP family genes involved in Zn transport have been characterized in model plants. Identification and functional characterization of certain ZIP genes, their relative expression and localization have been done only in a few crops such as rice and maize. More information on Zn transporters are available for rice plants when compared to other crops so this can serve as a model to study Zn transport in other cereal crops. ZIP family members involved in Zn transport and sequestration represent some of the clearest candidate genes for increased Zn content in crops. But very little information is available on how Zn is transported from leaf xylem to phloem of developing seeds and ultimately unloaded into seeds with the help of ZIP genes. This needs further research in the coming years. Identification and characterization of ZIP proteins and related TFs in many crops would help for the better understanding of Zn homeostasis. A more holistic and high-resolution studies on ZIP transporters in crops will help to overcome the problems associated with low Zn soils and to improve the human health.

## Data Availability Statement

All datasets generated for this study are included in the article/Supplementary Material.

## Author Contributions

TA, TM, and SA conceptualized and wrote the manuscript. TA and SA analyzed the protein sequences with alignment, modeling and phylogeny. GV and SI assisted, and TA, TM, GV, and SA edited, and updated the manuscript. SA and SI contributed critically in revising and improving the manuscript for publication.

## Conflict of Interest

The authors declare that the research was conducted in the absence of any commercial or financial relationships that could be construed as a potential conflict of interest.

## References

[B1] Alagarasan G. Dubey M. Aswathy K. S. Chandel G. (2017). Genome wide identification of orthologous ZIP genes associated with zinc and iron translocation in *Setaria italica*. *Front. Plant Sci.* 8:775. 10.3389/fpls.2017.00775 28555148 PMC5430159

[B2] Ali S. Khan A. R. Mairaj G. Arif M. Fida M. Bibi S. (2008). Assessment of different crop nutrient management practices for yield improvement. *Aust. J. Crop Sci.* 2 150–157.

[B3] Alloway B. (2009). Soil factors associated with zinc deficiency in crops and humans. *Environ. Geochem. Health* 31 537–548. 10.1007/s10653-009-9255-4 19291414

[B4] Andreini C. Banci L. Bertini I. Rosato A. (2006). Zinc through the three domains of life. *J. Proteome Res.* 5 3173–3178. 10.1021/pr0603699 17081069

[B5] Assunção A. Persson D. Husted S. Schjørring J. Alexander R. Aarts M. (2013). Model of how plants sense zinc deficiency. *Metallomics* 5 1110–1116. 10.1039/c3mt00070b 23851954

[B6] Assunção A. G. Herrero E. Lin Y. F. Huettel B. Talukdar S. Smaczniak C. (2010). *Arabidopsis thaliana* transcription factors bZIP19 and bZIP23 regulate the adaptation to zinc deficiency. *Proc. Natl. Acad. Sci. U.S.A.* 107 10296–10301. 10.1073/pnas.1004788107 20479230 PMC2890486

[B7] Astudillo C. Fernandez A. Blair M. W. Cichy K. A. (2013). The *Phaseolus vulgaris* ZIP gene family: identification, characterization, mapping, and gene expression. *Front. Plant Sci.* 4:286. 10.3389/fpls.2013.00286 23908661 PMC3726863

[B8] Astudillo-Reyes C. Fernandez A. C. Cichy K. A. (2015). Transcriptome characterization of developing bean (*Phaseolus vulgaris* L.) pods from two genotypes with contrasting seed zinc concentrations. *PLoS One* 10:e0137157. 10.1371/journal.pone.0137157 26367119 PMC4569411

[B9] Bashir K. Ishimaru Y. Nishizawa N. K. (2012). Molecular mechanisms of zinc uptake and translocation in rice. *Plant Soil* 36 189–201. 10.1007/s11104-012-1240-5

[B10] Bashir K. Rasheed S. Kobayashi T. Seki M. Nishizawa N. K. (2016). Regulating subcellular metal homeostasis: the key to crop improvement. *Front. Plant Sci.* 7:1192. 10.3389/fpls.2016.01192 27547212 PMC4974246

[B11] Bennetzen J. L. Schmutz J. Wang H. Percifield R. Hawkins J. Pontaroli A. C. (2012). Reference genome sequence of the model plant *Setaria*. *Nat. Biotechnol.* 30 555–561. 10.1038/nbt.2196 22580951

[B12] Black R. E. Allen L. H. Bhutta Z. A. Caulfield L. E. De Onis M. Ezzati M. (2008). Maternal and child undernutrition: global and regional exposures and health consequences. *Lancet* 371 243–260. 10.1016/S0140-6736(07)61690-0 18207566

[B13] Bouain N. Shahzad Z. Rouached A. Khan G. A. Berthomieu P. Abdelly C. (2014). Phosphate and zinc transport and signalling in plants: toward a better understanding of their homeostasis interaction. *J. Exp. Bot.* 30 5725–5741. 10.1093/jxb/eru314 25080087

[B14] Briat J. F. Rouached H. Tissot N. Gaymard F. Dubos C. (2015). Integration of P, S, Fe, and Zn nutrition signals in *Arabidopsis thaliana*: potential involvement of phosphate starvation response 1 (PHR1). *Front. Plant Sci.* 28:290. 10.3389/fpls.2015.00290 25972885 PMC4411997

[B15] Brown P. H. Cakmak I. Zhang Q. (1993). “Form and function of zinc plants,” in *Zinc in Soils and Plants. Developments in Plant and Soil Sciences*, Vol. 55 ed. Robson A. D. (Dordrecht: Springer), 93–106. 10.1007/978-94-011-0878-2_7

[B16] Bughio N. Yamaguchi H. Nishizawa N. K. Nakanishi H. Mori S. (2002). Cloning an iron−regulated metal transporter from rice. *J. Exp. Bot*. 53 1677–1682. 10.1093/jxb/erf004 12096107

[B17] Cakmak I. (2000). Tansley review no. 111 Possible roles of zinc in protecting plant cells from damage by reactive oxygen species. *New Phytol.* 146 185–205. 10.1046/j.1469-8137.2000.00630.x 33862977

[B18] Cakmak I. (2008). Enrichment of cereal grains with zinc: agronomic or genetic biofortification? *Plant Soil* 302 1–17. 10.1007/s11104-007-9466-3

[B19] Cakmak I. Marschner H. Bangerth F. (1989). Effect of zinc nutritional status on growth, protein metabolism and levels of indole-3-acetic acid and other phytohormones in bean (*Phaseolus vulgaris* L.). *J. Exp. Bot.* 40 405–412. 10.1093/jxb/40.3.405

[B20] Castro P. H. Lilay G. H. Muñoz-Mérida A. Schjoerring J. K. Azevedo H. Assunção A. G. (2017). Phylogenetic analysis of F-bZIP transcription factors indicates conservation of the zinc deficiency response across land plants. *Sci. Rep.* 7 1–14. 10.1038/s41598-017-03903-6 28630437 PMC5476651

[B21] Ceasar S. A. Hodge A. Baker A. Baldwin S. A. (2014). Phosphate concentration and arbuscular mycorrhizal colonisation influence the growth, yield and expression of twelve PHT1 family phosphate transporters in foxtail millet (*Setaria italica*). *PLoS One* 9:e108459. 10.1371/journal.pone.0108459 25251671 PMC4177549

[B22] Chen W. Feng Y. Chao Y. (2008). Genomic analysis and expression pattern of OsZIP1, OsZIP3, and OsZIP4 in two rice (*Oryza sativa* L.) genotypes with different zinc efficiency. *Russ. J. Plant Physiol.* 55 400–409. 10.1134/S1021443708030175

[B23] Chiang H. C. Lo J. C. Yeh K. C. (2006). Genes associated with heavy metal tolerance and accumulation in Zn/Cd hyperaccumulator *Arabidopsis halleri*: a genomic survey with cDNA microarray. *Environ. Sci. Technol.* 40 6792–6798. 10.1021/es061432y 17144312

[B24] Claus J. Chavarría-Krauser A. (2012). Modeling regulation of zinc uptake via ZIP transporters in yeast and plant roots. *PLoS One* 8:e37193. 10.1371/journal.pone.0037193 22715365 PMC3371047

[B25] Clemens S. (2001). Molecular mechanisms of plant metal tolerance and homeostasis. *Planta* 212 475–486. 10.1007/s004250000458 11525504

[B26] Conte S. S. Walker E. L. (2011). Transporters contributing to iron trafficking in plants. *Mol. Plant* 4 464–476. 10.1093/mp/ssr015 21447758

[B27] Corrêa L. G. G. Riaño-Pachón D. M. Schrago C. G. dos Santos R. V. Mueller-Roeber B. Vincentz M. (2008). The role of bZIP transcription factors in green plant evolution: adaptive features emerging from four founder genes. *PLoS One* 3:e2944. 10.1371/journal.pone.0002944 18698409 PMC2492810

[B28] Deshpande P. Dapkekar A. Oak M. Paknikar K. Rajwade J. (2018). Nanocarrier-mediated foliar zinc fertilization influences expression of metal homeostasis related genes in flag leaves and enhances gluten content in durum wheat. *PLoS One* 13:e0191035. 10.1371/journal.pone.0191035 29342185 PMC5771588

[B29] Devaiah B. N. Karthikeyan A. S. Raghothama K. G. (2007a). WRKY75 transcription factor is a modulator of phosphate acquisition and root development in *Arabidopsis*. *Plant Physiol.* 143 1789–1801. 10.1104/pp.106.093971 17322336 PMC1851818

[B30] Devaiah B. N. Madhuvanthi R. Karthikeyan A. S. Raghothama K. G. (2009). Phosphate starvation responses and gibberellic acid biosynthesis are regulated by the MYB62 transcription factor in *Arabidopsis*. *Mol. Plant* 2 43–58. 10.1093/mp/ssn081 19529828 PMC2639739

[B31] Devaiah B. N. Nagarajan V. K. Raghothama K. G. (2007b). Phosphate homeostasis and root development in *Arabidopsis* are synchronized by the zinc finger transcription factor ZAT6. *Plant Physiol.* 145 147–159. 10.1104/pp.107.101691 17631527 PMC1976576

[B32] Doust A. N. Kellogg E. A. Devos K. M. Bennetzen J. L. (2009). Foxtail millet: a sequence-driven grass model system. *Plant Physiol.* 149 137–141. 10.1104/pp.108.129627 19126705 PMC2613750

[B33] Durmaz E. Coruh C. Dinler G. Grusak M. A. Peleg Z. Saranga Y. (2011). Expression and cellular localization of ZIP1 transporter under zinc deficiency in wild emmer wheat. *Plant Mol. Biol. Rep.* 29 582–596. 10.1007/s11105-010-0264-3

[B34] Eide D. Broderius M. Fett J. Guerinot M. L. (1996). A novel iron-regulated metal transporter from plants identified by functional expression in yeast. *Proc. Natl. Acad. Sci. U.S.A.* 93 5624–5628. 10.1073/pnas.93.11.5624 8643627 PMC39298

[B35] Eide D. J. (1998). The molecular biology of metal ion transport in *Saccharomyces cerevisiae*. *Annu. Rev. Nutr.* 18 441–469. 10.1146/annurev.nutr.18.1.441 9706232

[B36] Eide D. J. (2005). “The zip family of zinc transporters,” in *Zinc Finger Proteins. Molecular Biology Intelligence Unit*, eds Iuchi S. Kuldell N. (Boston, MA: Springer), 261–264. 10.1007/0-387-27421-9_35

[B37] Eide D. J. (2006). Zinc transporters and the cellular trafficking of zinc. *BBA Mol. Cell Res.* 1763 711–722. 10.1016/j.bbamcr.2006.03.005 16675045

[B38] Ekiz H. Bagci S. Kiral A. Eker S. Gültekin I. Alkan A. (1998). Effects of zinc fertilization and irrigation on grain yield and zinc concentration of various cereals grown in zinc−deficient calcareous soils. *J. Plant Nutr.* 21 2245–2256. 10.1080/01904169809365558

[B39] Ekweagwu E. Agwu A. Madukwe E. (2008). The role of micronutrients in child health: a review of the literature. *Afr. J. Biotechnol.* 7 3805–3810.

[B40] Eswar N. Webb B. Marti−Renom M. A. Madhusudhan M. S. Eramian D. Shen M. Y. (2007). Comparative protein structure modeling using MODELLER. *Curr. Protoc. Protein Sci.* 50 2–9. 10.1002/0471140864.ps0209s50 18429317

[B41] Etebu E. Nwauzoma A. (2014). A review on sweet orange (*Citrus sinensis* L Osbeck): health, diseases and management. *Am. J. Res. Commun.* 2 33–70.

[B42] Evens N. P. Buchner P. Williams L. E. Hawkesford M. J. (2017). The role of ZIP transporters and group F bZIP transcription factors in the Zn−deficiency response of wheat (*Triticum aestivum*). *Plant J.* 92 291–304. 10.1111/tpj.13655 28771859 PMC5656842

[B43] FAO (2000). *Calcareous Soils. FAO AGL Land and Plant Nutrition Management Services.* Available online at: www.fao.org/ag/agl/agll/prosoil/calc.htm (accessed February 24, 2009).

[B44] Fei X. Fu X. Z. Wang N. Q. Xi J. L. Huang Y. Wei Z. (2016). Physiological changes and expression characteristics of ZIP family genes under zinc deficiency in navel orange (*Citrus sinensis*). *J. Integr. Agric.* 15 803–811. 10.1016/S2095-3119(15)61276-X

[B45] Fu X. Z. Zhou X. Xing F. Ling L. L. Chun C. P. Cao L. (2017). Genome-wide identification, cloning and functional analysis of the zinc/iron-regulated transporter-like protein (ZIP) gene family in trifoliate orange (*Poncirus trifoliata* L. Raf.). *Front. Plant Sci.* 8:588. 10.3389/fpls.2017.00588 28469631 PMC5395618

[B46] Gaitán-Solís E. Taylor N. J. Siritunga D. Stevens W. Schachtman D. P. (2015). Overexpression of the transporters AtZIP1 and AtMTP1 in cassava changes zinc accumulation and partitioning. *Front. Plant Sci.* 6:492. 10.3389/fpls.2015.00492 26217349 PMC4496839

[B47] Gepts P. Aragão F. J. De Barros E. Blair M. W. Brondani R. Broughton W. (2008). “Genomics of phaseolus beans, a major source of dietary protein and micronutrients in the tropics,” in *Genomics of Tropical Crop Plants. Plant Genetics and Genomics: Crops and Models*, Vol. 1 eds Moore P. H. Ming R. (New York, NY: Springer), 113–143. 10.1007/978-0-387-71219-2_5

[B48] Grotz N. Fox T. Connolly E. Park W. Guerinot M. L. Eide D. (1998). Identification of a family of zinc transporter genes from *Arabidopsis* that respond to zinc deficiency. *Proc. Natl. Acad. Sci.U.S.A.* 95 7220–7224. 10.1073/pnas.95.12.7220 9618566 PMC22785

[B49] Grotz N. Guerinot M. L. (2006). Molecular aspects of Cu, Fe and Zn homeostasis in plants. *BBA Mol. Cell. Res.* 1763 595–608. 10.1016/j.bbamcr.2006.05.014 16857279

[B50] Guerinot M. L. (2000). The ZIP family of metal transporters. *BBA Biomembranes* 1465 190–198. 10.1016/s0005-2736(00)00138-3 10748254

[B51] Hacisalihoglu G. Hart J. J. Kochian L. V. (2001). High-and low-affinity zinc transport systems and their possible role in zinc efficiency in bread wheat. *Plant Physiol.* 125 456–463. 10.1104/pp.125.1.456 11154353 PMC61026

[B52] Hacisalihoglu G. Kochian L. V. (2003). How do some plants tolerate low levels of soil zinc? Mechanisms of zinc efficiency in crop plants. *New Phytol.* 159 341–350. 10.1046/j.1469-8137.2003.00826.x 33873363

[B53] Hanikenne M. Talke I. N. Haydon M. J. Lanz C. Nolte A. Motte P. (2008). Evolution of metal hyperaccumulation required cis-regulatory changes and triplication of HMA4. *Nature* 453:391. 10.1038/nature06877 18425111

[B54] Hänsch R. Mendel R. R. (2009). Physiological functions of mineral micronutrients (Cu, Zn, Mn, Fe, Ni, Mo, B, Cl). *Curr. Opin. Plant Biol.* 12 259–266. 10.1016/j.pbi.2009.05.006 19524482

[B55] Henriques A. Farias D. de Oliveira Costa A. (2017). Identification and characterization of the bZIP transcription factor involved in zinc homeostasis in cereals. *Genet. Mol. Res.* 16 1–10. 10.4238/gmr16029558 28671251

[B56] Horak V. Trčka I. (1976). The influence of Zn^2+^ ions on the tryptophan biosynthesis in plants. *Biol. Plant.* 18 393–396. 10.1007/BF02922471

[B57] Hussain D. Haydon M. J. Wang Y. Wong E. Sherson S. M. Young J. (2004). P-type ATPase heavy metal transporters with roles in essential zinc homeostasis in *Arabidopsis*. *Plant Cell* 16 1327–1339. 10.1105/tpc.020487 15100400 PMC423219

[B58] Impa S. M. Morete M. J. Ismail A. M. Schulin R. Johnson-Beebout S. E. (2013). Zn uptake, translocation and grain Zn loading in rice (*Oryza sativa* L.) genotypes selected for Zn deficiency tolerance and high grain Zn. *J. Exp. Bot.* 22 2739–2751. 10.1093/jxb/ert118 23698631 PMC3697949

[B59] Inaba S. Kurata R. Kobayashi M. Yamagishi Y. Mori I. Ogata Y. (2015). Identification of putative target genes of bZIP19, a transcription factor essential for *Arabidopsis* adaptation to Zn deficiency in roots. *Plant J.* 84 323–334. 10.1111/tpj.12996 26306426

[B60] Ishimaru Y. Masuda H. Suzuki M. Bashir K. Takahashi M. Nakanishi H. (2007). Overexpression of the OsZIP4 zinc transporter confers disarrangement of zinc distribution in rice plants. *J. Exp. Bot.* 58 2909–2915. 10.1093/jxb/erm147 17630290

[B61] Ishimaru Y. Suzuki M. Kobayashi T. Takahashi M. Nakanishi H. Mori S. (2005). OsZIP4, a novel zinc-regulated zinc transporter in rice. *J. Exp. Bot.* 56 3207–3214. 10.1093/jxb/eri317 16263903

[B62] Ishimaru Y. Suzuki M. Tsukamoto T. Suzuki K. Nakazono M. Kobayashi T. (2006). Rice plants take up iron as an Fe^3+^−phytosiderophore and as Fe^2+^. *Plant J*. 45 335–346. 10.1111/j.1365-313X.2005.02624.x 16412081

[B63] Ishimaru Y. Bashir K. Nishizawa N. K. (2011). Zn uptake and translocation in rice plants. *Rice* 4 21–27. 10.1007/s12284-011-9061-3

[B64] Jakoby M. Weisshaar B. Dröge-Laser W. Vicente-Carbajosa J. Tiedemann J. Kroj T. (2002). bZIP transcription factors in *Arabidopsis*. *Trends Plant Sci.* 7 106–111. 10.1016/s1360-1385(01)02223-3 11906833

[B65] Kaiser C. Kilburn M. R. Clode P. L. Fuchslueger L. Koranda M. Cliff J. B. (2015). Exploring the transfer of recent plant photosynthates to soil microbes: mycorrhizal pathway vs direct root exudation. *New Phytol.* 205 1537–1551. 10.1111/nph.13138 25382456 PMC4357392

[B66] Kavitha P. Kuruvilla S. Mathew M. (2015). Functional characterization of a transition metal ion transporter, OsZIP6 from rice (*Oryza sativa* L.). *Plant Physiol. Biochem.* 97 165–174. 10.1016/j.plaphy.2015.10.005 26476396

[B67] Khan G. A. Bouraine S. Wege S. Li Y. de Carbonnel M. Berthomieu P. (2014). Coordination between zinc and phosphate homeostasis involves the transcription factor PHR1, the phosphate exporter PHO1, and its homologue PHO1; H3 in *Arabidopsis*. *J. Exp. Bot.* 13 871–884. 10.1093/jxb/ert444 24420568 PMC3924728

[B68] Krishna T. P. A. Ceasar S. A. Maharajan T. Ramakrishnan M. Duraipandiyan V. Al-Dhabi N. (2017). Improving the zinc-use efficiency in plants: a review. *SABRAO J. Breed. Genet.* 49 221–230.

[B69] Krithika S. Balachandar D. (2016). Expression of zinc transporter genes in rice as influenced by zinc-solubilizing *Enterobacter cloacae* strain ZSB14. *Front. Plant Sci.* 7:446. 10.3389/fpls.2016.00446 27092162 PMC4822286

[B70] Kumar L. Meena N. L. Singh U. Singh U. Praharaj C. Singh S., et al. (eds) (2016). “Zinc transporter: mechanism for improving Zn availability,” in *Biofortification of Food Crops* (New Delhi: Springer), 129–146. 10.1007/978-81-322-2716-8_11

[B71] Lata C. Gupta S. Prasad M. (2013). Foxtail millet: a model crop for genetic and genomic studies in bioenergy grasses. *Crit. Rev. Biotechnol.* 33 328–343. 10.3109/07388551.2012.716809 22985089

[B72] Lee S. An G. (2009). Over−expression of OsIRT1 leads to increased iron and zinc accumulations in rice. *Plant Cell Environ.* 32 408–416. 10.1111/j.1365-3040.2009.01935.x 19183299

[B73] Lee S. Jeong H. J. Kim S. A. Lee J. Guerinot M. L. An G. (2010a). OsZIP5 is a plasma membrane zinc transporter in rice. *Plant Mol. Biol.* 73 507–517. 10.1007/s11103-010-9637-0 20419467

[B74] Lee S. Kim S. A. Lee J. Guerinot M. L. An G. (2010b). Zinc deficiency-inducible OsZIP8 encodes a plasma membrane-localized zinc transporter in rice. *Mol. Cells* 29 551–558. 10.1007/s10059-010-0069-0 20496122

[B75] Li S. Zhou X. Huang Y. Zhu L. Zhang S. Zhao Y. (2013). Identification and characterization of the zinc-regulated transporters, iron-regulated transporter-like protein (ZIP) gene family in maize. *BMC Plant Biol.* 13:114. 10.1186/1471-2229-13-114 23924433 PMC3751942

[B76] Li S. Zhou X. Li H. Liu Y. Zhu L. Guo J. (2015). Overexpression of ZmIRT1 and ZmZIP3 enhances iron and zinc accumulation in transgenic *Arabidopsis*. *PLoS One* 10:e0136647. 10.1371/journal.pone.0136647 26317616 PMC4552944

[B77] Lilay G. H. Castro P. H. Campilho A. Assunção A. G. (2018). The *Arabidopsis* bZIP19 and bZIP23 activity requires zinc deficiency–insight on regulation from complementation lines. *Front. Plant Sci.* 9:1955. 10.3389/fpls.2018.01955 30723487 PMC6349776

[B78] Lilay G. H. Castro P. H. Guedes J. G. Almeida D. M. Campilho A. Azevedo H. (2020). Rice F-bZIP transcription factors regulate the zinc deficiency response. *J. Exp. Bot.* 71 1–14. 10.1093/jxb/eraa115 32133499 PMC7307843

[B79] Lin Y. F. Liang H. M. Yang S. Y. Boch A. Clemens S. Chen C. C. (2009). *Arabidopsis* IRT3 is a zinc−regulated and plasma membrane localized zinc/iron transporter. *New Phytol.* 182 392–404. 10.1111/j.1469-8137.2009.02766.x 19210716

[B80] Lindsay W. (1972). Zinc in soils and plant nutrition. *Adv. Agron.* 24 147–186. 10.1016/S0065-2113(08)60635-5

[B81] Marichali A. Dallali S. Ouerghemmi S. Sebei H. Hosni K. (2014). Germination, morpho-physiological and biochemical responses of coriander (*Coriandrum sativum* L.) to zinc excess. *Ind. Crops Prod.* 55 248–257. 10.1016/j.indcrop.2014.02.033

[B82] Marschner H. (1995). *Marschner’s Mineral Nutrition of Higher Plants.* Cambridge, MA: Academic press.

[B83] Marschner H. (2011). *Marschner’s Mineral Nutrition of Higher Plants.* Cambridge, MA: Academic press.

[B84] Mäser P. Thomine S. Schroeder J. I. Ward J. M. Hirschi K. Sze H. (2001). Phylogenetic relationships within cation transporter families of *Arabidopsis*. *Plant Physiol.* 126 1646–1667. 10.1104/pp.126.4.1646 11500563 PMC117164

[B85] Meng L. Sun L. Tan L. (2018). Progress in ZIP transporter gene family in rice. *Yi Chuan* 40 33–43. 10.16288/j.yczz.17-238 29367191

[B86] Menguer P. K. Vincent T. Miller A. J. Brown J. K. Vincze E. Borg S. (2018). Improving zinc accumulation in cereal endosperm using HvMTP1, a transition metal transporter. *Plant Biotechnol. J.* 16 63–71. 10.1111/pbi.12749 28436146 PMC5785336

[B87] Milner M. J. Seamon J. Craft E. Kochian L. V. (2013). Transport properties of members of the ZIP family in plants and their role in Zn and Mn homeostasis. *J. Exp. Bot.* 64 369–381. 10.1093/jxb/ers315 23264639 PMC3528025

[B88] Mitra G. N. (2015). “Zinc (Zn) uptake,” in *Regulation of Nutrient Uptake by Plants – A Biochemical and Molecular Approach* (New Delhi: Springer), 127–133.

[B89] Mondal T. K. Ganie S. A. Rana M. K. Sharma T. R. (2013). Genome-wide analysis of zinc transporter genes of maize (*Zea mays*). *Plant Mol. Biol. Rep.* 32 605–616. 10.1007/s11105-013-0664-2

[B90] Moreau S. Thomson R. M. Kaiser B. N. Trevaskis B. Guerinot M. L. Udvardi M. K. (2002). GmZIP1 encodes a symbiosis-specific zinc transporter in soybean. *J. Biol. Chem.* 277 4738–4746. 10.1074/jbc.M106754200 11706025

[B91] Mousavi S. R. (2011). Zinc in crop production and interaction with phosphorus. *Aust. J. Basic Appl. Sci.* 5 1503–1509.

[B92] Nakanishi H. Ogawa I. Ishimaru Y. Mori S. Nishizawa N. K. (2006). Iron deficiency enhances cadmium uptake and translocation mediated by the Fe^2+^ transporters OsIRT1 and OsIRT2 in rice. *Soil Sci. Plant Nutr.* 52 464–469. 10.1111/j.1747-0765.2006.00055.x

[B93] Nazri A. Z. Griffin J. H. Peaston K. A. Alexander−Webber D. G. Williams L. E. (2017). F−group bZIPs in barley–a role in Zn deficiency. *Plant Cell Environ.* 40 2754–2770. 10.1111/pce.13045 28763829 PMC5656896

[B94] Nene Y. (1966). Symptoms, cause and control of Khaira disease of paddy. *Bull. Indian Phytopathol. Soc.* 3 97–191.

[B95] Nijhawan A. Jain M. Tyagi A. K. Khurana J. P. (2008). Genomic survey and gene expression analysis of the basic leucine zipper transcription factor family in rice. *Plant Physiol.* 146 333–350. 10.1104/pp.107.112821 18065552 PMC2245831

[B96] Palmer L. E. Rabinowicz P. D. Shaughnessy A. L. Balija V. S. Nascimento L. U. Dike S. (2003). Maize genome sequencing by methylation filtration. *Science* 302 2115–2117. 14684820 10.1126/science.1091265

[B97] Palmgren M. G. Clemens S. Williams L. E. Krämer U. Borg S. Schjørring J. K. (2008). Zinc biofortification of cereals: problems and solutions. *Trends Plant Sci.* 13 464–473. 10.1016/j.tplants.2008.06.005 18701340

[B98] Pedas P. Husted S. (2009). Zinc transport mediated by barley ZIP proteins are induced by low pH. *Plant Signal. Behav.* 4 842–845. 10.4161/psb.4.9.9375 19847115 PMC2802790

[B99] Pedas P. Schjoerring J. K. Husted S. (2009). Identification and characterization of zinc-starvation-induced ZIP transporters from barley roots. *Plant Physiol. Biochem.* 47 377–383. 10.1016/j.plaphy.2009.01.006 19249224

[B100] Pellegrino E. Bedini S. (2014). Enhancing ecosystem services in sustainable agriculture: biofertilization and biofortification of chickpea (*Cicer arietinum* L.) by arbuscular mycorrhizal fungi. *Soil Biol. Biochem.* 68 429–439. 10.1016/j.soilbio.2013.09.030

[B101] Pellegrino E. Bosco S. Ciccolini V. Pistocchi C. Sabbatini T. Silvestri N. (2015). Agricultural abandonment in Mediterranean reclaimed peaty soils: long-term effects on soil chemical properties, arbuscular mycorrhizas and CO2 flux. *Agric. Ecosyst. Environ.* 199 164–175. 10.1016/j.agee.2014.09.004

[B102] Rai V. Sanagala R. Sinilal B. Yadav S. Sarkar A. K. Dantu P. K. (2015). Iron availability affects phosphate deficiency-mediated responses, and evidence of cross-talk with auxin and zinc in *Arabidopsis*. *Plant Cell Physiol.* 56 1107–1123. 10.1093/pcp/pcv035 25759329

[B103] Ramegowda Y. Venkategowda R. Jagadish P. Govind G. Hanumanthareddy R. R. Makarla U. (2013). Expression of a rice Zn transporter, OsZIP1, increases Zn concentration in tobacco and finger millet transgenic plants. *Plant Biotechnol. Rep.* 7 309–319. 10.1007/s11816-012-0264-x

[B104] Ramesh S. A. Choimes S. Schachtman D. P. (2004). Over-expression of an *Arabidopsis* zinc transporter in *Hordeum vulgare* increases short-term zinc uptake after zinc deprivation and seed zinc content. *Plant Mol. Biol.* 54 373–385. 10.1023/B:PLAN.0000036370.70912.34 15284493

[B105] Ramesh S. A. Shin R. Eide D. J. Schachtman D. P. (2003). Differential metal selectivity and gene expression of two zinc transporters from rice. *Plant Physiol.* 133 126–134. 10.1104/pp.103.026815 12970480 PMC196588

[B106] Regmi B. D. Rengel Z. Khabaz-Saberi H. O. (2010). Zinc deficiency in agricultural systems and its implication to human health. *Int. J. Environ. Rural Dev.* 1 98–103.

[B107] Rubio V. Linhares F. Solano R. Martín A. C. Iglesias J. Leyva A. (2001). A conserved MYB transcription factor involved in phosphate starvation signaling both in vascular plants and in unicellular algae. *Genes Dev.* 15 2122–2133. 10.1101/gad.204401 11511543 PMC312755

[B108] Ruel M. T. Bouis H. E. (1998). Plant breeding: a long-term strategy for the control of zinc deficiency in vulnerable populations. *Am. J. Clin. Nutr.* 68 488–494. 10.1093/ajcn/68.2.488S 9701166

[B109] Sadeghzadeh B. (2013). A review of zinc nutrition and plant breeding. *J. Soil Sci. Plant Nutr.* 13 905–927. 10.4067/S0718-95162013005000072

[B110] Samreen T. Shah H. U. Ullah S. Javid M. (2017). Zinc effect on growth rate, chlorophyll, protein and mineral contents of hydroponically grown mungbeans plant (*Vigna radiata*). *Arab. J. Chem.* 10 S1802–S1807. 10.1016/j.arabjc.2013.07.005

[B111] Sasaki A. Yamaji N. Mitani−Ueno N. Kashino M. Ma J. F. (2015). A node−localized transporter OsZIP3 is responsible for the preferential distribution of Zn to developing tissues in rice. *Plant J* 84 374–384. 10.1111/tpj.13005 26332571

[B112] Sharma P. Chatterjee C. Sharma C. Nautiyal N. Agarwala S. (1979). Effect of zinc deficiency on the development and physiology of wheat pollen. *J. Indian Bot. Soc.* 58 330–334.

[B113] Sharma P. N. Chatterjee C. Sharma C. P. Agarwala S. C. (1987). Zinc deficiency and anther development in maize. *Plant Cell Physiol.* 28 11–18. 10.1093/oxfordjournals.pcp.a077265 16815956

[B114] Shaul O. Hilgemann D. W. Almeida−Engler J. Van Montagu M. Inzé D. Galili G. (1999). Cloning and characterization of a novel Mg^2+/^H^+^ exchanger. *EMBO J.* 15 3973–3980. 10.1093/emboj/18.14.3973 10406802 PMC1171473

[B115] Shiferaw B. Prasanna B. M. Hellin J. Bänziger M. (2011). Crops that feed the world 6. Past successes and future challenges to the role played by maize in global food security. *Food Secur.* 3 307–327. 10.1007/s12571-011-0140-5

[B116] Shiferaw B. Smale M. Braun H. J. Duveiller E. Reynolds M. Muricho G. (2013). Crops that feed the world 10. Past successes and future challenges to the role played by wheat in global food security. *Food Secur.* 5 291–317. 10.1007/s12571-013-0263-y

[B117] Skoog F. (1940). Relationships between zinc and auxin in the growth of higher plants. *Am. J. Bot.* 27 939–951. 10.1002/j.1537-2197.1940.tb13958.x

[B118] Suzuki M. Bashir K. Inoue H. Takahashi M. Nakanishi H. Nishizawa N. K. (2012). Accumulation of starch in Zn-deficient rice. *Rice* 5 1–8. 10.1186/1939-8433-5-9 27234235 PMC5520845

[B119] Suzuki M. Takahashi M. Tsukamoto T. Watanabe S. Matsuhashi S. Yazaki J. (2006). Biosynthesis and secretion of mugineic acid family phytosiderophores in zinc−deficient barley. *Plant J.* 48 85–97. 10.1111/j.1365-313X.2006.02853.x 16972867

[B120] Tamura K. Stecher G. Peterson D. Filipski A. Kumar S. (2013). MEGA6: molecular evolutionary genetics analysis version 6.0. *Mol. Biol. Evol.* 16 2725–2729. 10.1093/molbev/mst197 PMC3840312 24132122

[B121] Tan L. Zhu Y. Fan T. Peng C. Wang J. Sun L. (2019). OsZIP7 functions in xylem loading in roots and inter-vascular transfer in nodes to deliver Zn/Cd to grain in rice. *Biochem. Biophys. Res. Commun.* 512 112–118. 10.1016/j.bbrc.2019.03.024 30871778

[B122] Tiong J. McDonald G. Genc Y. Shirley N. Langridge P. Huang C. Y. (2015). Increased expression of six ZIP family genes by zinc (Zn) deficiency is associated with enhanced uptake and root−to−shoot translocation of Zn in barley (*Hordeum vulgare*). *New Phytol.* 207 1097–1109. 10.1111/nph.13413 25904503

[B123] Tiong J. McDonald G. K. Genc Y. Pedas P. Hayes J. E. Toubia J. (2014). HvZIP7 mediates zinc accumulation in barley (*Hordeum vulgare*) at moderately high zinc supply. *New Phytol.* 201 131–143. 10.1111/nph.12468 24033183

[B124] Tsui C. (1948). The role of zinc in auxin synthesis in the tomato plant. *Am. J. Bot.* 35 172–179. 18909962

[B125] Van de Mortel J. E. Villanueva L. A. Schat H. Kwekkeboom J. Coughlan S. Moerland P. D. (2006). Large expression differences in genes for iron and zinc homeostasis, stress response, and lignin biosynthesis distinguish roots of *Arabidopsis thaliana* and the related metal hyperaccumulator *Thlaspi caerulescens*. *Plant Physiol.* 142 1127–1147. 10.1104/pp.106.082073 16998091 PMC1630723

[B126] Vatansever R. Özyigit I. I. Filiz E. (2016). Comparative and phylogenetic analysis of zinc transporter genes/proteins in plants. *Turk. J. Biol.* 40 600–611. 10.3906/biy-1501-91 16179994

[B127] Vert G. Briat J. F. Curie C. (2001). *Arabidopsis* IRT2 gene encodes a root−periphery iron transporter. *Plant J.* 26 181–189. 10.1046/j.1365-313x.2001.01018.x 11389759

[B128] Wang H. Jin J. (2005). Photosynthetic rate, chlorophyll fluorescence parameters, and lipid peroxidation of maize leaves as affected by zinc deficiency. *Photosynthetica* 43 591–596. 10.1007/s11099-005-0092-0

[B129] Watts-Williams S. J. Cavagnaro T. R. (2018). Arbuscular mycorrhizal fungi increase grain zinc concentration and modify the expression of root ZIP transporter genes in a modern barley (*Hordeum vulgare*) cultivar. *Plant Sci.* 274 163–170. 10.1016/j.plantsci.2018.05.015 30080600

[B130] Wissuwa M. Ismail A. M. Graham R. D. (2008). Rice grain zinc concentrations as affected by genotype, native soil-zinc availability, and zinc fertilization. *Plant Soil* 306 37–48. 10.1007/s11104-007-9368-4

[B131] Xie X. Hu W. Fan X. Chen H. Tang M. (2019). Interactions between phosphorus, zinc, and iron homeostasis in non mycorrhizal and mycorrhizal plants. *Front. Plant Sci.* 10:1172 10.3389/fpls.2019.01172 PMC6775243 31616454

[B132] Xi-wen Y. Xiao-hong T. Xin-chun L. William G. Yu-xian C. (2011). Foliar zinc fertilization improves the zinc nutritional value of wheat (*Triticum aestivum* L.) grain. *Afri. J. Biotechnol.* 10 14778–14785. 10.5897/AJB11.780

[B133] Yang X. Huang J. Jiang Y. Zhang H. S. (2009). Cloning and functional identification of two members of the ZIP (Zrt, Irt-like protein) gene family in rice (*Oryza sativa* L.). *Mol. Bio. Rep.* 36 281–287. 10.1007/s11033-007-9177-0 18038191

[B134] Yoshida S. Tanaka A. (1969). Zinc deficiency of the rice plant in calcareous soils. *Soil Sci. Plant Nutr.* 15 75–80. 10.1080/00380768.1969.10432783

[B135] Zhang G. Liu X. Quan Z. Cheng S. Xu X. Pan S. (2012). Genome sequence of foxtail millet (*Setaria italica*) provides insights into grass evolution and biofuel potential. *Nat. Biotechnol.* 30 549–553. 10.1038/nbt.2195 22580950

[B136] Zhang T. Liu J. Fellner M. Zhang C. Sui D. Hu J. (2017). Crystal structures of a ZIP zinc transporter reveal a binuclear metal center in the transport pathway. *Sci. Adv.* 3:e1700344. 10.1126/sciadv.1700344 28875161 PMC5573306

[B137] Zhao H. Eide D. (1996a). The yeast ZRT1 gene encodes the zinc transporter protein of a high-affinity uptake system induced by zinc limitation. *Proc. Natl. Acad. Sci. U.S.A.* 93 2454–2458. 10.1073/pnas.93.6.2454 8637895 PMC39818

[B138] Zhao H. Eide D. (1996b). The ZRT2 gene encodes the low affinity zinc transporter in *Saccharomyces cerevisiae*. *J. Biol. Chem.* 271 23203–23210. 10.1074/jbc.271.38.23203 8798516

[B139] Zimin A. V. Puiu D. Hall R. Kingan S. Clavijo B. J. Salzberg S. L. (2017). The first near-complete assembly of the hexaploid bread wheat genome, *Triticum aestivum*. *GigaScience* 6 1–7. 10.1093/gigascience/gix097 29069494 PMC5691383

