# Supplementary material for: Structure, Function, Regulation and Phylogenetic Relationship of ZIP Family Transporters of Plants
Source: Front Plant Sci. 2020 May 27;11:662. doi: 10.3389/fpls.2020.00662 (PMC7267038; doi:10.3389/fpls.2020.00662)
Supplement: Supplementary file 1 [file Data_Sheet_1.docx]

Supplementary Material

# Figure S1. Multiple sequence alignment ZIP transporters of *Bordetella bronchiseptica* (BbZIP), *Arabidopsis* (AtZIP), rice (OsZIP) and maize (ZmZIP) transporters. The protein sequences were aligned by ClustalW alignment using molecular evolutionary genetics analysis, V 6.0 (MEGA6) tool (Tamura et al., 2013).

**
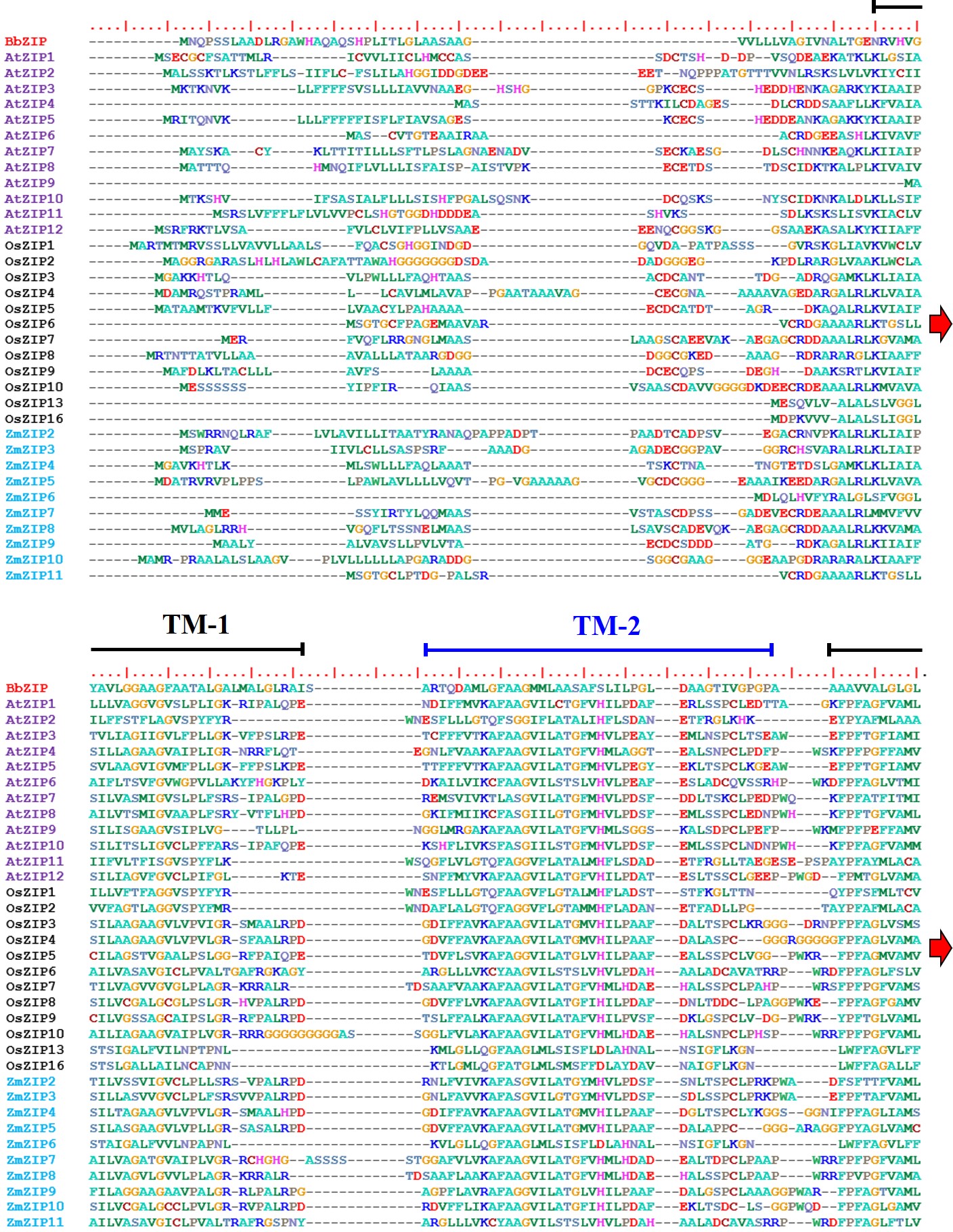
**

#
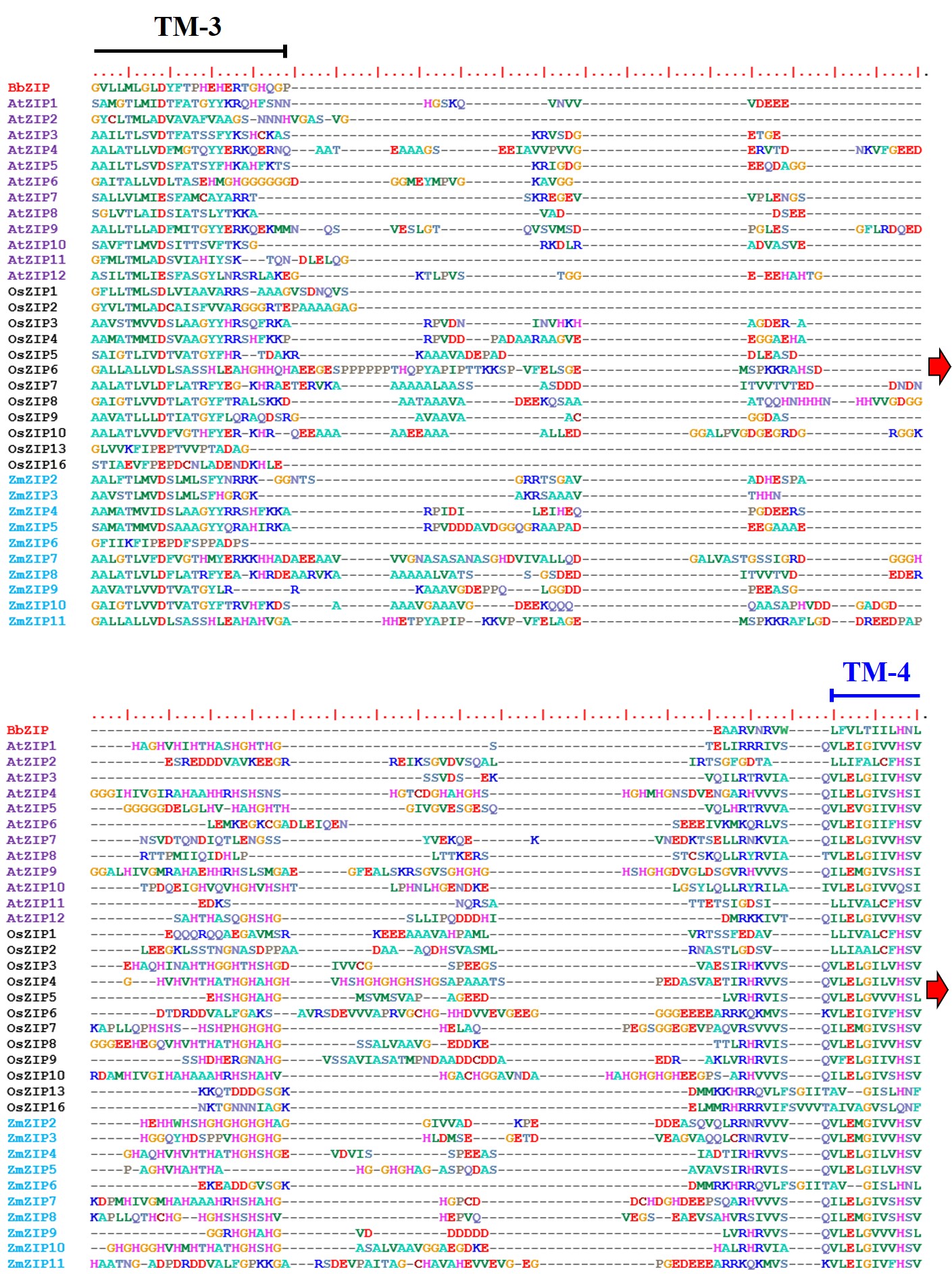


#
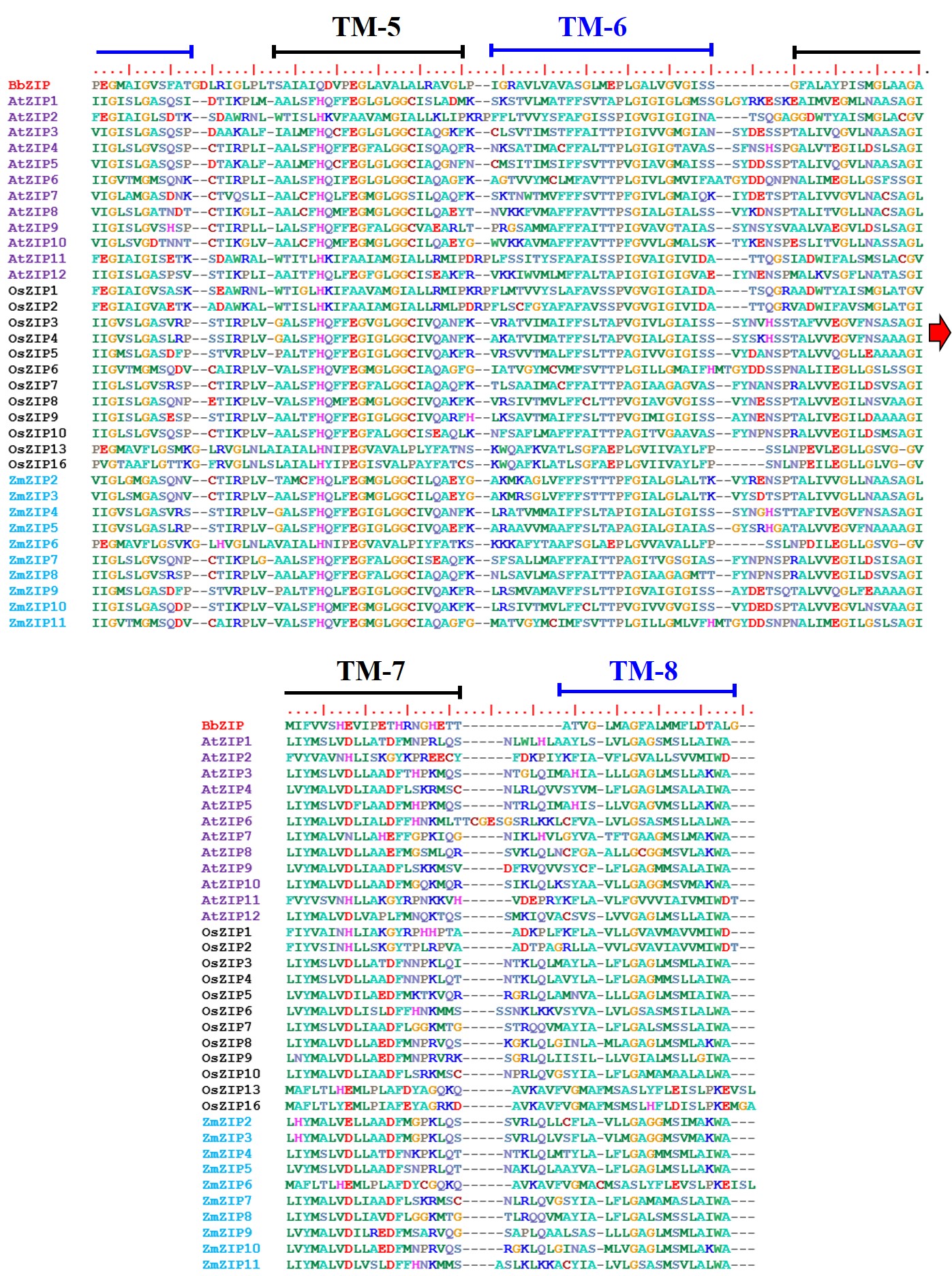


**Table S1.** Information on ZIP transporter genes, chromosome location, protein length, number of TM domains, localization and site of expression reported in various plants.

| **Plant Name** | **Gene** | **Chromosome**  **location** | **Protein length** | **TM domains** | **Localization** | **Expression under Zn deficiency** | **Reference** |
| --- | --- | --- | --- | --- | --- | --- | --- |
| *Poncirus trifoliate* | *PtZIP1* | - | 351 | 6 | Plasma membrane | Leaf and root | Fu et al., 2017 |
|  | *PtZIP2* | - | 334 | 9 | Plasma membrane | Leaf and root |  |
|  | *PtZIP3* | - | 335 | 9 | Plasma membrane | Leaf and root |  |
|  | *PtZIP5* | - | 359 | 8 | Plasma membrane | Leaf and root |  |
|  | *PtZIP6* | - | 350 | 8 | Plasma membrane | Leaf and root |  |
|  | *PtZIP7* | - | 347 | 7 | Plasma membrane | Leaf and root |  |
|  | *PtZIP9* | - | 419 | 6 | Plasma membrane | Leaf and root |  |
|  | *PtZIP11* | - | 347 | 9 | Plasma membrane | Leaf and root |  |
|  | *PtZIP12* | - | 357 | 7 | Plasma membrane | Leaf and root |  |
|  | *PtZIP13* | - | 361 | 7 | Plasma membrane | Leaf and root |  |
|  | *PtZIP14* | - | 358 | 9 | Plasma membrane | Leaf and root |  |
|  |  |  |  |  |  |  |  |
| *Setaria italica* | *SiZIP1* | Chr3 | 377 | 6 | - | Root, leaf, stem and spica tissue | Alagarasan et al., 2017 |
|  | *SiZIP2* | Chr3 | 389 | 8 | - | Root, leaf, stem and spica tissue |  |
|  | *SiZIP3* | Chr6 | 277 | 6 | - | Root, leaf, stem and spica tissue |  |
|  | *SiZIP4* | Chr7 | 406 | 7 | - | Root, leaf, stem and spica tissue |  |
|  | *SiZIP5* | Chr7 | 390 | 8 | - | Root, leaf, stem and spica tissue |  |
|  | *SiZIP6* | Chr9 | 354 | 8 | - | Root, leaf, stem and spica tissue |  |
|  | *SiZIP7* | Chr9 | 367 | 8 | - | Root, leaf, stem and spica tissue |  |
|  |  |  |  |  |  |  |  |
| *Glycine max* | *GmZIP1* | - | 359 | - | Peribacteroid membrane | Nodules | Moreau et al., 2002 |
|  | *GmZIP4* | - | 364 | - | - | - |  |
|  | *GmZIP6* | - | 324 | - | - | - |  |
|  | *GmZIP10* | - | 356 | - | - | - |  |
|  | *GmZIP11* | - | 349 | - | - | - |  |
|  |  |  |  |  |  |  |  |
| *Oryza sativa* | *OsZIP1* | Chr1 | 352 | - | Vascular bundle | Shoot and root | Chen et al., 2008; Ishimaru et al., 2005; Yang et al., 2009; Ramesh et al., 2004; Lee et al., 2010a,b |
|  | *OsZIP2* | Chr3 | 358 | - | Vascular bundle | Shoot and root |  |
|  | *OsZIP3* | Chr4 | 364 | - | Vascular bundle & epidermis | Shoot, leaves and root |  |
|  | *OsZIP4* | Chr8 | 396 | - | Epidermal cell | Shoot and root |  |
|  | *OsZIP5* | Chr5 | 353 | - | - | Shoot and root |  |
|  | *OsZIP6* | Chr5 | 395 | - | - | Shoot and root |  |
|  | *OsZIP7* | Chr5 | 384 | 8 | Plasma membrane | Roots, culms, leaves spikelets, and shoot |  |
|  | *OsZIP8* | Chr2 | 390 | 8 | Plasma membrane | Roots, culms, leaves and spikelets |  |
|  | *OsZIP9* | Chr5 | 362 | - | - | - |  |
|  | *OsZIP10* | Chr5 | 404 | - | - | - |  |
|  | *OsZIP11* | Chr5 | 577 | - | - | - |  |
|  | *OsZIP12* | Chr6 | 276 | - | - | - |  |
|  | *OsZIP13* | Chr7 | 498 | - | - | - |  |
|  | *OsZIP14* | Chr8 | 289 | - | - | - |  |
|  | *OsZIP15* | Chr8 | 498 | - | - | - |  |
|  | *OsZIP16* | Chr8 | 282 | - | - | - |  |
|  |  |  |  |  |  |  |  |
| *Phaseolus vulgaris* | *PvZIP1* | Chr01 | - | - | - | - | Astudillo et al., 2013 |
|  | *PvZIP2* | Chr01 | - | - | - | - |  |
|  | *PvZIP3* | Chr01 | - | - | - | - |  |
|  | *PvZIP4* | Chr02 | - | - | - | - |  |
|  | *PvZIP5* | Chr05 | - | - | - | - |  |
|  | *PvZIP6* | Chr05 | - | - | - | - |  |
|  | *PvZIP7* | Chr05 | - | - | - | - |  |
|  | *PvZIP8* | Chr05 | - | - | - | - |  |
|  | *PvZIP9* | Chr06 | - | - | - | - |  |
|  | *PvZIP10* | Chr06 | - | - | - | - |  |
|  | *PvZIP11* | Chr06 | - | - | - | - |  |
|  | *PvZIP12* | Chr06 | - | - | - | Root, leaf and pod |  |
|  | *PvZIP13* | Chr06 | - | - | - | Root, leaf and pod |  |
|  | *PvZIP14* | Chr08 | - | - | - | - |  |
|  | *PvZIP15* | Chr08 | - | - | - | - |  |
|  | *PvZIP16* | Chr08 | - | - | - | Root, leaf and pod |  |
|  | *PvZIP17* | Chr10 | - | - | - | - |  |
|  | *PvZIP18* | Chr11 | - | - | - | - |  |
|  | *PvZIP19* | Chr02 | - | - | - | - |  |
|  |  |  |  |  |  |  |  |
| *Zea mays* | *ZmZIP1* | Chr1 | 490 | 7 | ER and Plasma membrane | Shoot and root | Li et al., 2013; Mondal et al., 2014 |
|  | *ZmZIP2* | Chr2 | 359 | 9 | ER and Plasma membrane | Shoot and root |  |
|  | *ZmZIP3* | Chr2 | 367 | 6 | ER and Plasma membrane | Shoot and root |  |
|  | *ZmZIP4* | Chr4 | 386 | 6 | ER and Plasma membrane | Shoot and root |  |
|  | *ZmZIP5* | Chr6 | 402 | 6 | ER and Plasma membrane | Shoot and root |  |
|  | *ZmZIP6* | Chr8 | 396 | 8 | ER and Plasma membrane | Shoot and root |  |
|  | *ZmZIP7* | Chr6 | 387 | 6 | ER and Plasma membrane | Shoot and root |  |
|  | *ZmZIP8* | Chr7 | 396 | 7 | ER and Plasma membrane | Shoot and root |  |
|  | *ZmZIP1* | Chr1 | 483 | 1 | Plasma membrane | Highly expressed in flag leaf |  |
|  | *ZmZIP2* | Chr1 | 381 | 1 | Plasma membrane | Highly expressed in flag leaf |  |
|  | *ZmZIP3* | Chr1 | 361 | 1 | Plasma membrane | Kernel |  |
|  | *ZmZIP4* | Chr2 | 367 | 2 | Plasma membrane | Highly expressed in flag leaf |  |
|  | *ZmZIP5* | Chr4 | 386 | 4 | Chloroplast & Plasma membrane | Highly expressed in flag leaf |  |
|  | *ZmZIP6* | Chr4 | 279 | 4 | Plasma membrane | Highly expressed in flag leaf |  |
|  | *ZmZIP7* | Chr6 | 402 | 6 | Chloroplast & Plasma membrane | Highly expressed in flag leaf |  |
|  | *ZmZIP8* | Chr6 | 387 | 6 | Plasma membrane | Highly expressed in flag leaf |  |
|  | *ZmZIP9* | Chr6 | 341 | 6 | Plasma membrane | Highly expressed in flag leaf |  |
|  | *ZmZIP10* | Chr7 | 397 | 7 | Plasma membrane | Highly expressed in flag leaf |  |
|  | *ZmZIP11* | Chr8 | 396 | 8 | Plasma membrane | Highly expressed in flag leaf |  |
|  | *ZmZIP12* | Chr10 | 573 | 10 | Plasma membrane | Kernel |  |
|  |  |  |  |  |  |  | Evens et al., 2017 |
| *Triticum aestivum* | *TaZIP1* |  | 355 | - | - | - |  |
|  | *TaZIP2* |  | 363 | - | - | - |  |
|  | *TaZIP3* |  | 360 | - | - | Shoot and root |  |
|  | *TaZIP5* |  | 349 | - | - | - |  |
|  | *TaZIP6* |  | 395 | - | - | Shoot and root |  |
|  | *TaZIP7* |  | 386 | - | - | Shoot and root |  |
|  | *TaZIP9* |  | - | - | - | Shoot and root |  |
|  | *TaZIP10* |  | 417 | - | - | - |  |
|  | *TaZIP11* |  | 577 | - | - | - |  |
|  | *TaZIP13* |  | 576 | - | - | Shoot and root |  |
|  | *TaZIP14* |  | 497 | - | - | - |  |
|  | *TaZIP16* |  | 273 | - | - | - |  |
|  |  |  |  |  |  |  |  |
| *Citrus sinensis* | *CsZIP1* | - | 352 | - | - | Leaf | Fei et al., 2016 |
|  | *CsZIP2* | Chr8 | 335 | - | - | Leaf |  |
|  | *CsZIP3* | - | 360 | - | - | Leaf |  |
|  | *CsZIP4* | Chr4 | 420 | - | - | Leaf |  |
|  |  |  |  |  |  |  |  |
| *Hordeum vulgare* | *HvZIP1* | - | 355 | - | - | - | Tiong et al., 2014; Tiong et al., 2015; Pedas et al., 2009 |
|  | *HvZIP2* | - | 359 | - | - | Shoot |  |
|  | *HvZIP3* | - | 362 | 8 | Plasma membrane | Shoot and root |  |
|  | *HvZIP5* | - | 350 | 8 | Plasma membrane, vacuolar membrane | Shoot and root |  |
|  | *HvZIP6* | - | 402 | - | - | - |  |
|  | *HvZIP7* | - | 386 | 8 | Plasma membrane | Shoot and root |  |
|  | *HvZIP8* | - | 359 | 8 | Plasma membrane | Shoot and root |  |
|  | *HvZIP10* | - | 414 | - | Plasma membrane | Shoot and root |  |
|  | *HvZIP11* | - | 577 | - | - | - |  |
|  | *HvZIP13* | - | 382 | - | Plasma membrane | Shoot and root |  |
|  | *HvZIP14* | - | 499 | - | - | - |  |
|  | *HvZIP16* | - | 276 | - | - | - |  |
|  |  |  |  |  |  |  |  |
| *Triticum durum* | *TdZIP1* | 3AL | - | - | Plasma membrane | Flag, non-flag leaves, stem and spike | Deshpande et al., 2018 |
|  | *TdZIP3* | 2AL & 2BL | - | - | Plasma membrane | Flag, non-flag leaves, stem and spike |  |
|  | *TdZIP7* | 1AS & 1BS | - | - | Plasma membrane | Flag, non-flag leaves, stem and spike |  |
|  | *TdZIP10* | 7AL & 7BL | - | - | Plasma membrane | Flag, non-flag leaves, stem and spike |  |
|  | *TdZIP15* | 3AS | - | - | Plasma membrane | Flag, non-flag leaves, stem and spike |  |
| *Triticum turgidum ssp. dicoccoides* | *TdZIP1* | - | - | 8 | ER | Root | Durmaz et al., 2011 |
|  |  |  |  |  |  |  |  |

**Supplementary file S1. Sequences of 113 ZIP proteins from 14 plant species used for phylogenetic tree construction.** These protein sequences were collected from phytozome website (<https://phytozome.jgi.doe.gov/pz/portal.html>).

**Arabidopsis**

**>AtZIP1**

MSECGCFSATTMLRICVVLIICLHMCCASSDCTSHDDPVSQDEAEKATKLKLGSIALLLVAGGVGVSLPLIGKRIPALQPENDIFFMVKAFAAGVILCTGFVHILPDAFERLSSPCLEDTTAGKFPFAGFVAMLSAMGTLMIDTFATGYYKRQHFSNNHGSKQVNVVVDEEEHAGHVHIHTHASHGHTHGSTELIRRRIVSQVLEIGIVVHSVIIGISLGASQSIDTIKPLMAALSFHQFFEGLGLGGCISLADMKSKSTVLMATFFSVTAPLGIGIGLGMSSGLGYRKESKEAIMVEGMLNAASAGILIYMSLVDLLATDFMNPRLQSNLWLHLAAYLSLVLGAGSMSLLAIWA

**>AtZIP2**

MALSSKTLKSTLFFLSIIFLCFSLILAHGGIDDGDEEEETNQPPPATGTTTVVNLRSKSLVLVKIYCIIILFFSTFLAGVSPYFYRWNESFLLLGTQFSGGIFLATALIHFLSDANETFRGLKHKEYPYAFMLAAAGYCLTMLADVAVAFVAAGSNNNHVGASVGESREDDDVAVKEEGRREIKSGVDVSQALIRTSGFGDTALLIFALCFHSIFEGIAIGLSDTKSDAWRNLWTISLHKVFAAVAMGIALLKLIPKRPFFLTVVYSFAFGISSPIGVGIGIGINATSQGAGGDWTYAISMGLACGVFVYVAVNHLISKGYKPREECYFDKPIYKFIAVFLGVALLSVVMIWD

**>AtZIP3**

MKTKNVKLLFFFFSVSLLLIAVVNAAEGHSHGGPKCECSHEDDHENKAGARKYKIAAIPTVLIAGIIGVLFPLLGKVFPSLRPETCFFFVTKAFAAGVILATGFMHVLPEAYEMLNSPCLTSEAWEFPFTGFIAMIAAILTLSVDTFATSSFYKSHCKASKRVSDGETGESSVDSEKVQILRTRVIAQVLELGIIVHSVVIGISLGASQSPDAAKALFIALMFHQCFEGLGLGGCIAQGKFKCLSVTIMSTFFAITTPIGIVVGMGIANSYDESSPTALIVQGVLNAASAGILIYMSLVDLLAADFTHPKMQSNTGLQIMAHIALLLGAGLMSLLAKWA

**>AtZIP4**

MASSTTKILCDAGESDLCRDDSAAFLLKFVAIASILLAGAAGVAIPLIGRNRRFLQTEGNLFVAAKAFAAGVILATGFVHMLAGGTEALSNPCLPDFPWSKFPFPGFFAMVAALATLLVDFMGTQYYERKQERNQAATEAAAGSEEIAVVPVVGERVTDNKVFGEEDGGGIHIVGIRAHAAHHRHSHSNSHGTCDGHAHGHSHGHMHGNSDVENGARHVVVSQILELGIVSHSIIIGLSLGVSQSPCTIRPLIAALSFHQFFEGFALGGCISQAQFRNKSATIMACFFALTTPLGIGIGTAVASSFNSHSPGALVTEGILDSLSAGILVYMALVDLIAADFLSKRMSCNLRLQVVSYVMLFLGAGLMSALAIWA

**>AtZIP5**

MRITQNVKLLLFFFFFISFLFIAVSAGESKCECSHEDDEANKAGAKKYKIAAIPSVLAAGVIGVMFPLLGKFFPSLKPETTFFFVTKAFAAGVILATGFMHVLPEGYEKLTSPCLKGEAWEFPFTGFIAMVAAILTLSVDSFATSYFHKAHFKTSKRIGDGEEQDAGGGGGGGDELGLHVHAHGHTHGIVGVESGESQVQLHRTRVVAQVLEVGIIVHSVVIGISLGASQSPDTAKALFAALMFHQCFEGLGLGGCIAQGNFNCMSITIMSIFFSVTTPVGIAVGMAISSSYDDSSPTALIVQGVLNAASAGILIYMSLVDFLAADFMHPKMQSNTRLQIMAHISLLVGAGVMSLLAKWA

**>AtZIP6**

MASCVTGTEAAIRAAACRDGEEASHLKIVAVFAIFLTSVFGVWGPVLLAKYFHGKPLYDKAILVIKCFAAGVILSTSLVHVLPEAFESLADCQVSSRHPWKDFPFAGLVTMIGAITALLVDLTASEHMGHGGGGGGDGGMEYMPVGKAVGGLEMKEGKCGADLEIQENSEEEIVKMKQRLVSQVLEIGIIFHSVIIGVTMGMSQNKCTIRPLIAALSFHQIFEGLGLGGCIAQAGFKAGTVVYMCLMFAVTTPLGIVLGMVIFAATGYDDQNPNALIMEGLLGSFSSGILIYMALVDLIALDFFHNKMLTTCGESGSRLKKLCFVALVLGSASMSLLALWA

**>AtZIP7**

MAYSKACYKLTTITILLLSFTLPSLAGNAENADVSECKAESGDLSCHNNKEAQKLKIIAIPSILVASMIGVSLPLFSRSIPALGPDREMSVIVKTLASGVILATGFMHVLPDSFDDLTSKCLPEDPWQKFPFATFITMISALLVLMIESFAMCAYARRTSKREGEVVPLENGSNSVDTQNDIQTLENGSSYVEKQEKVNEDKTSELLRNKVIAQILELGIVVHSVVIGLAMGASDNKCTVQSLIAALCFHQLFEGMGLGGSILQAQFKSKTNWTMVFFFSVTTPFGIVLGMAIQKIYDETSPTALIVVGVLNACSAGLLIYMALVNLLAHEFFGPKIQGNIKLHVLGYVATFTGAAGMSLMAKWA

**>AtZIP8**

MATTTQHMNQIFLVLLLISFAISPAISTVPKECETDSTDSCIDKTKALPLKIVAIVAILVTSMIGVAAPLFSRYVTFLHPDGKIFMIIKCFASGIILGTGFMHVLPDSFEMLSSPCLEDNPWHKFPFTGFVAMLSGLVTLAIDSIATSLYTKKAVADDSEERTTPMIIQIDHLPLTTKERSSTCSKQLLRYRVIATVLELGIIVHSVVIGLSLGATNDTCTIKGLIAALCFHQMFEGMGLGGCILQAEYTNVKKFVMAFFFAVTTPSGIALGIALSSVYKDNSPTALITVGLLNACSAGLLIYMALVDLLAAEFMGSMLQRSVKLQLNCFGAALLGCGGMSVLAKWA

**>AtZIP9**

MASILISGAAGVSIPLVGTLLPLNGGLMRGAKAFAAGVILATGFVHMLSGGSKALSDPCLPEFPWKMFPFPEFFAMVAALLTLLADFMITGYYERKQEKMMNQSVESLGTQVSVMSDPGLESGFLRDQEDGGALHIVGMRAHAEHHRHSLSMGAEGFEALSKRSGVSGHGHGHSHGHGDVGLDSGVRHVVVSQILEMGIVSHSIIIGISLGVSHSPCTIRPLLLALSFHQFFEGFALGGCVAEARLTPRGSAMMAFFFAITTPIGVAVGTAIASSYNSYSVAALVAEGVLDSLSAGILVYMALVDLIAADFLSKKMSVDFRVQVVSYCFLFLGAGMMSALAIWA

**>AtZIP10**

MTKSHVIFSASIALFLLLSISHFPGALSQSNKDCQSKSNYSCIDKNKALDLKLLSIFSILITSLIGVCLPFFARSIPAFQPEKSHFLIVKSFASGIILSTGFMHVLPDSFEMLSSPCLNDNPWHKFPFAGFVAMMSAVFTLMVDSITTSVFTKSGRKDLRADVASVETPDQEIGHVQVHGHVHSHTLPHNLHGENDKELGSYLQLLRYRILAIVLELGIVVQSIVIGLSVGDTNNTCTIKGLVAALCFHQMFEGMGLGGCILQAEYGWVKKAVMAFFFAVTTPFGVVLGMALSKTYKENSPESLITVGLLNASSAGLLIYMALVDLLAADFMGQKMQRSIKLQLKSYAAVLLGAGGMSVMAKWA

**>AtZIP11**

MSRSLVFFFLFLVLVVPCLSHGTGGDHDDDEASHVKSSDLKSKSLISVKIACLVIIFVLTFISGVSPYFLKWSQGFLVLGTQFAGGVFLATALMHFLSDADETFRGLLTAEGESEPSPAYPFAYMLACAGFMLTMLADSVIAHIYSKTQNDLELQGEDKSNQRSATTETSIGDSILLIVALCFHSVFEGIAIGISETKSDAWRALWTITLHKIFAAIAMGIALLRMIPDRPLFSSITYSFAFAISSPIGVAIGIVIDATTQGSIADWIFALSMSLACGVFVYVSVNHLLAKGYRPNKKVHVDEPRYKFLAVLFGVVVIAIVMIWDT

**>AtZIP12**

MSRFRKTLVSAFVLCLVIFPLLVSAAEEENQCGGSKGGSAAEKASALKYKIIAFFSILIAGVFGVCLPIFGLKTESNFFMYVKAFAAGVILATGFVHILPDATESLTSSCLGEEPPWGDFPMTGLVAMAASILTMLIESFASGYLNRSRLAKEGKTLPVSTGGEEEHAHTGSAHTHASQGHSHGSLLIPQDDDHIDMRKKIVTQILELGIVVHSVIIGISLGASPSVSTIKPLIAAITFHQLFEGFGLGGCISEAKFRVKKIWVMLMFFALTAPIGIGIGIGVAEIYNENSPMALKVSGFLNATASGILIYMALVDLVAPLFMNQKTQSSMKIQVACSVSLVVGAGLMSLLAIWA

**2. Rice**

**>OsZIP1**

MARTMTMRVSSLLVAVVLLAALSFQACSGHGGINDGDGQVDAPATPASSSGVRSKGLIAVKVWCLVILLVFTFAGGVSPYFYRWNESFLLLGTQFAAGVFLGTALMHFLADSTSTFKGLTTNQYPFSFMLTCVGFLLTMLSDLVIAAVARRSAAAGVSDNQVSEQQQRQQAEGAVMSRKEEEAAAVAHPAMLVRTSSFEDAVLLIVALCFHSVFEGIAIGVSASKSEAWRNLWTIGLHKIFAAVAMGIALLRMIPKRPFLMTVVYSLAFAVSSPVGVGIGIAIDATSQGRAADWTYAISMGLATGVFIYVAINHLIAKGYRPHHPTAADKPLFKFLAVLLGVAVMAVVMIWD

**>OsZIP2**

MAGGRGARASLHLHLAWLCAFATTAWAHGGGGGGGDSDADADGGGEGKPDLRARGLVAAKLWCLAVVFAGTLAGGVSPYFMRWNDAFLALGTQFAGGVFLGTAMMHFLADANETFADLLPGTAYPFAFMLACAGYVLTMLADCAISFVVARGGGRTEPAAAAGAGLEEGKLSSTNGNASDPPAADAAAQDHSVASMLRNASTLGDSVLLIAALCFHSVFEGIAIGVAETKADAWKALWTISLHKIFAAIAMGIALLRMLPDRPFLSCFGYAFAFAVSSPVGVGIGIVIDATTQGRVADWIFAVSMGLATGIFIYVSINHLLSKGYTPLRPVAADTPAGRLLAVVLGVAVIAVVMIWDT

**>OsZIP3**

MGAKKHTLQVLPWLLLFAQHTAASACDCANTTDGADRQGAMKLKLIAIASILAAGAAGVLVPVIGRSMAALRPDGDIFFAVKAFAAGVILATGMVHILPAAFDALTSPCLKRGGGDRNPFPFAGLVSMSAAVSTMVVDSLAAGYYHRSQFRKARPVDNINVHKHAGDERAEHAQHINAHTHGGHTHSHGDIVVCGSPEEGSVAESIRHKVVSQVLELGILVHSVIIGVSLGASVRPSTIRPLVGALSFHQFFEGVGLGGCIVQANFKVRATVIMAIFFSLTAPVGIVLGIAISSSYNVHSSTAFVVEGVFNSASAGILIYMSLVDLLATDFNNPKLQINTKLQLMAYLALFLGAGLMSMLAIWA

**>OsZIP4**

MDAMRQSTPRAMLLLCAVLMLAVAPPGAATAAAVAGCECGNAAAAAVAGEDARGALRLKLVAIASILAAGAAGVLVPVLGRSFAALRPDGDVFFAVKAFAAGVILATGMVHILPAAFDALASPCGGGRGGGGGFPFAGLVAMAAAMATMMIDSVAAGYYRRSHFKKPRPVDDPADAARAAGVEEGGAEHAGHVHVHTHATHGHAHGHVHSHGHGHGHSHGSAPAAATSPEDASVAETIRHRVVSQVLELGILVHSVIIGVSLGASLRPSSIRPLVGALSFHQFFEGIGLGGCIVQANFKAKATVIMATFFSLTAPVGIALGIAISSSYSKHSSTALVVEGVFNSAAAGILIYMSLVDLLAADFNNPKLQTNTKLQLAVYLALFLGAGMMSLLAIWA

**>OsZIP5**

MATAAMTKVFVLLFLVAACYLPAHAAAAECDCATDTAGRDKAQALRLKVIAIFCILAGSTVGAALPSLGGRFPAIQPETDVFLSVKAFAGGVILATGLVHILPAAFEALSSPCLVGGPWKRFPFAGMVAMVSAIGTLIVDTVATGYFHRTDAKRKAAAVADEPADDLEASDEHSHGHAHGMSVMSVAPAGEEDLVRHRVISQVLELGVVVHSLIIGMSLGASDFPSTVRPLVPALTFHQFFEGIGLGGCIVQAKFRVRSVVTMALFFSLTTPAGIVVGIGISSVYDANSPTALVVQGLLEAAAAGILVYMALVDILADFMKTKVQRRGRLQLAMNVALLLGAGLMSMIAIWA

**>OsZIP6**

MSGTGCFPAGEMAAVARVCRDGAAAARLKTGSLLAILVASAVGICLPVALTGAFRGKAGYARGLLLVKCYAAGVILSTSLVHVLPDAHAALADCAVATRRPWRDFPFAGLFSLVGALLALLVDLSASSHLEAHGHHQHAEEGESPPPPPPTHQPYAPIPTTKKSPVFELSGEMSPKKRAHSDDTDRDDVALFGAKSAVRSDEVVVAPRVGCHGHHDVVEVGEEGGGGEEEEARRKQKMVSKVLEIGIVFHSVIIGVTMGMSQDVCAIRPLVVALSFHQVFEGMGLGGCIAQAGFGIATVGYMCVMFSVTTPLGILLGMAIFHMTGYDDSSPNALIIEGLLGSLSSGILVYMALVDLISLDFFHNKMMSSSNKLKKVSYVALVLGSASMSILALWA

**>OsZIP7**

MERFVQFLRRGNGLMAASLAAGSCAEEVAKAEGAGCRDDAAALRLKGVAMATILVAGVVGVGLPLAGRKRRALRTDSAAFVAAKAFAAGVILATGFVHMLHDAEHALSSPCLPAHPWRSFPFPGFVAMSAALATLVLDFLATRFYEGKHRAETERVKAAAAAALAASSASDDDITVVTVTEDDNDNKAPLLQPHSHSHSHPHGHGHGHELAQPEGSGGEGEVPAQVRSVVVSQILEMGIVSHSVIIGLSLGVSRSPCTIRPLVAALSFHQFFEGFALGGCIAQAQFKTLSAAIMACFFAITTPAGIAAGAGVASFYNANSPRALVVEGILDSVSAGILIYMSLVDLIAADFLGGKMTGSTRQQVMAYIALFLGALSMSSLAIWA

**>OsZIP8**

MRTNTTATVLLAAAVALLLATAARGDGGDGGCGKEDAAAGRDRARARGLKIAAFFSILVCGALGCGLPSLGRHVPALRPDGDVFFLVKAFAAGVILATGFIHILPDAFDNLTDDCLPAGGPWKEFPFAGFGAMVGAIGTLVVDTLATGYFTRALSKKDAATAAAVADEEKQSAAATQQHNHHHNHHVVGDGGGGGEEHEGQVHVHTHATHGHAHGSSALVAAVGEDDKETTLRHRVISQVLELGIVVHSVIIGISLGASQNPETIKPLVVALSFHQMFEGMGLGGCIVQAKFKVRSIVTMVLFFCLTTPVGIAVGVGISSVYNESSPTALVVEGILNSVAAGILIYMALVDLLAEDFMNPRVQSKGKLQLGINLAMLAGAGLMSMLAKWA

**>OsZIP9**

MAFDLKLTACLLLAVFSLAAAADCECQPSDEGHDAAKSRTLKVIAIFCILVGSSAGCAIPSLGRRFPALRPDTSLFFALKAFAAGVILATAFVHILPVSFDKLGSPCLVDGPWRKYPFTGLVAMLAAVATLLLDTIATGYFLQRAQDSRGAVAAVAACGGDASSSHDHERGNAHGVSSAVIASATMPNDAADDCDDAEDRAKLVRHRVISQVFELGIIVHSIIIGISLGASESPSTIRPLVAALTFHQFFEGIGLGGCIVQARFHLKSAVTMAIFFSLTTPVGIMIGIGISSAYNENSPTALIVEGILDAAAAGILNYMALVDLLAEDFMNPRVRKSGRLQLIISILLLVGIALMSLLGIWA

**>OsZIP10**

MESSSSSSYIPFIRQIAASVSAASCDAVVGGGGDKDEECRDEAAALRLKMVAVAAILIAGAAGVAIPLVGRRRRGGGGGGGGGASSGGLFVLAKAFAAGVILATGFVHMLHDAEHALSNPCLPHSPWRRFPFPGFVAMLAALATLVVDFVGTHFYERKHRQEEAAAAAEEAAAALLEDGGALPVGDGEGRDGRGGKRDAMHIVGIHAHAAAHRHSHAHVHGACHGGAVNDAHAHGHGHGHEEGPSARHVVVSQILELGIVSHSVIIGLSLGVSQSPCTIKPLVAALSFHQFFEGFALGGCISEAQLKNFSAFLMAFFFAITTPAGITVGAAVASFYNPNSPRALVVEGILDSMSAGILIYMALVDLIAADFLSRKMSCNPRLQVGSYIALFLGAMAMAALALWA

**>OsZIP13**

MESQVLVALALSLVGGLSTSIGALFVILNPTPNLKMLGLLQGFAAGLMLSISFLDLAHNALNSIGFLKGNLWFFAGVLFFGLVVKFIPEPTVVPTADAGKKQTDDDGSGKDMMKKHRRQVLFSGIITAVGISLHNFPEGMAVFLGSMKGLRVGLNLAIAIALHNIPEGVAVALPLYFATNSKWQAFKVATLSGFAEPLGVIIVAYLFPSSLNPEVLEGLLGSVGGVMAFLTLHEMLPLAFDYAGQKQAVKAVFVGMAFMSASLYFLEISLPKEVSL

**>OsZIP16**

MDPKVVVALALSLIGGLSTSLGALLAILNCAPNNKTLGMLQGFATGLMLSMSFFDLAYDAVNAIGFLKGNLWFFAGALLFSTIAEVFPEPDCNLADENDKHLENKTGNNNIAGKELMMRHRRRVIFSVVVTAIVAGVSLQNFPVGTAAFLGTTKGFRVGLNLSLAIALHYIPEGISVALPAYFATCSKWQAFKLATLSGFAEPLGVIIVAYLFPSNLNPEILEGLLGLVGGVMAFLTLYEMLPIAFEYAGRKDAVKAVFVGMAFMSMSLHFLDISLPKEMGA

**3. Maize**

**>ZmZIP2**

MSWRRNQLRAFLVLAVILLITAATYRANAQPAPPADPTPAADTCADPSVEGACRNVPKALRLKLIAIPTILVSSVIGVCLPLLSRSVPALRPDRNLFVIVKAFASGVILATGYMHVLPDSFSNLTSPCLPRKPWADFSFTTFVAMLAALFTLMVDSLMLSFYNRRKGGNTSGRRTSGAVADHESPAHEHHWHSHGHGHGHGHAGGIVVADKPEDDEASQVQLRRNRVVVQVLEMGIVVHSVVIGLGMGASQNVCTIRPLVTAMCFHQLFEGMGLGGCILQAEYGAKMKAGLVFFFSTTTPFGIALGLALTKVYRENSPTALIVVGLLNAASAGLLHYMALVELLAADFMGPKLQSSVRLQLLCFLAVLLGAGGMSIMAKWA

**>ZmZIP3**

MSPRAVIIVLCLLSASPSRFAAADGAGADECGGPAVGGRCHSVARALRLKLIAIPSILLASVVGVCLPLFSRSVVPALRPDGNLFAVVKAFASGVILGTGYMHVLPDSFSDLSSPCLPRKPWAEFPFTAFVAMLAAVSTLMVDSLMLSFHGRGKAKRSAAAVTHHNHGGQYHDSPPVHGHGHGHLDMSEGETDVEAGVAQQLCRNRVIVQVLEMGIVVHSVVIGLSMGASQNVCTIRPLVAALSFHQLFEGMGLGGCILQAEYGAKMRSGLVFFFSTTTPFGIALGLALTKVYSDTSPTALIVVGLLNAASAGLLHYMALVDLLAADFMGPKLQSSVRLQLVSFLAVLMGAGGMSVMAKWA

**>ZmZIP4**

MGAVKHTLKMLSWLLLFAQLAAATTSKCTNATNGTETDSLGAMKLKLIAIASILTAGAAGVLVPVLGRSMAALHPDGDIFFAVKAFAAGVILATGMVHILPAAFDGLTSPCLYKGGSGGNIFPFAGLIAMSAAMATMVIDSLAAGYYRRSHFKKARPIDILEIHEQPGDEERSGHAQHVHVHTHATHGHSHGEVDVISSPEEASIADTIRHRVVSQVLELGILVHSVIIGVSLGASVRSSTIRPLVGALSFHQFFEGIGLGGCIVQANFKLRATVMMAIFFSLTAPIGIALGIGISSSYNGHSTTAFIVEGVFNSASAGILIYMSLVDLLATDFNKPKLQTNTKLQLMTYLALFLGAGMMSMLAIWA

**>ZmZIP5**

MDATRVRVPLPPSLPAWLAVLLLLVQVTPGVGAAAAAGVGCDCGGGEAAAIKEEDARGALRLKLVAVASILASGAAGVLVPLLGRSASALRPDGDVFFAVKAFAAGVILATGMVHILPAAFDALAPPCGGGARAGGFPYAGLVAMCSAMATMMVDSAAAGYYQRAHIRKARPVDDDAVDGGQGRAAPADEEGAAAEPAGHVHAHTHAHGGHGHAGASPQDASAVAVSIRHRVISQVLELGILVHSVIIGVSLGASLRPSTIRPLVGALSFHQFFEGIGLGGCIVQAEFKARAAVVMAAFFSLTAPAGIALGIAIASGYSRHGATALVVEGVFNAAAAGILVYMSLVDLLAADFSNPRLQTNAKLQLAAYVALFLGAGLMSLLAKWA

**>ZmZIP6**

MDLQLHVFYRALGLSFVGGLSTAIGALFVVLNPAPNLKVLGLLQGFAAGLMLSISFLDLAHNALNSIGFLKGNLWFFAGVLFFGFIIKFIPEPDFSPPADPSEKEADDGVSGKDMMRKHRRQVLFSGIITAVGISLHNLPEGMAVFLGSVKGLHVGLNLAVAIALHNIPEGVAVALPIYFATKSKKKAFYTAAFSGLAEPLGVVAVALLFPSSLNPDILEGLLGSVGGVMAFLTLHEMLPLAFDYCGQKQAVKAVFVGMACMSASLYFLEVSLPKEISL

**>ZmZIP7**

MMESSYIRTYLQQMAASVSTASCDPSSGADEVECRDEAAALRLMMVFVVAILVAGATGVAIPLVGRRCHGHGASSSSSTGGAFVLVKAFAAGVILATGFVHMLHDADEALTDPCLPAAPWRRFPFPGFVAMLAALGTLVFDFVGTHMYERKKHHADAEEAAVVVGNASASANASGHDVIVALLQDGALVASTGSSIGRDGGGHKDPMHIVGMHAHAAAHRHSHAHGHGPCDDCHDGHDEEPSQARHVVVSQILELGIVSHSVIIGLSLGVSQNPCTIKPLGAALSFHQFFEGFALGGCISEAQFKSFSALLMAFFFAITTPAGITVGSGIASFYNPNSPRALVVEGILDSISAGILIYMALVDLIAADFLSKRMSCNLRLQVGSYIALFLGAMAMASLAIWA

**>ZmZIP8**

MVLAGLRRHVGQFLTSSNELMAASLSAVSCADEVQKAEGAGCRDDAAALRLKKVAMAAILVAGVLGVVLPLAGRKRRALRTDSAAFLAAKAFAAGVILATGFVHMLHDAEHALSSPCLPAAPWRRFPVPGFVAMAAALATLVLDFLATRFYEAKHRDEAARVKAAAAAALVATSSGSDEDITVVTVDEDERKAPLLQTHCHGHGHSHSHSHVHEPVQVEGSEAEVSAHVRSIVVSQILEMGIVSHSVIIGLSLGVSRSPCTIRPLVAALAFHQFFEGFALGGCIAQAQFKNLSAVLMASFFAITTPAGIAAGAGMTTFYNPNSPRALVVEGILDSVSAGILIYMSLVDLIAVDFLGGKMTGTLRQQVMAYIALFLGALSMSSLAIWA

**>ZmZIP9**

MAALYALVAVSLLPVLVTAECDCSDDDATGRDKAGALRLKIIAIFFILAGGAAGAAVPALGRRLPALRPGAGPFLAVRAFAGGVILATGLVHILPAAFDALGSPCLAAAGGPWARFPFAGTVAMLAAVATLVVDTVATGYLRRKAAAVGDEPPQLGGDDPEEASGGGRHGHAHGVDDDDDDLVRHRVVSQVLELGVVVHSLIIGMSLGASDFPSTVRPLVPALTFHQLFEGIGLGGCIVQAKFRLRSMVAMAVFFSLTTPIGVAIGIGISSAYDETSQTALVVQGLFEAAAAGILVYMALVDILREDFMSARVQGSAPLQAALSASLLLGAGLMSMLAIWA

**>ZmZIP10**

MAMRPRAALALSLAAGVPLVLLLLLLAPGARADDGSGGCGAAGGGEAAPGDRARARALKIAAFFSILVCGALGCCLPVLGRRVPALRPDRDVFFLIKAFAAGVILATGFIHILPDAFEKLTSDCLSGGPWQDFPFAGLGAMVGAIGTLVVDTVATGYFTRVHFKDSAAAAVGAAAVGDEEKQQQQAASAPHVDDGADGDGHGHGGHVHMHTHATHGHSHGASALVAAVGGAEGDKEHALRHRVIAQVLELGIVVHSVIIGISLGASQDPSTIKPLVVALSFHQMFEGMGLGGCIVQAKFKLRSIVTMVLFFCLTTPVGIVVGVGISSVYDEDSPTALVVEGVLNSVAAGILVYMALVDLLAEDFMNPRVQSRGKLQLGINASMLVGAGLMSMLAKWA

**>ZmZIP11**

MSGTGCLPTDGPALSRVCRDGAAAARLKTGSLLAILVASAVGICLPVALTRAFRGSPNYARGLLLVKCYAAGVILSTSLVHVLPDAHAALADCAVASRRPWRDFPFAGLFTLVGALLALLVDLSASSHLEAHAHVGAHHETPYAPIPKKVPVFELAGEMSPKKRAFLGDDREEDPAPHAATNGADPDRDDVALFGPKKGARSDEVPAITAGCHAVAHEVVEVGEGPGEDEEEARRKQKMVSKVLEIGIVFHSVIIGVTMGMSQDVCAIRPLVVALSFHQVFEGMGLGGCIAQAGFGMATVGYMCIMFSVTTPLGILLGMLVFHMTGYDDSNPNALIMEGILGSLSAGILIYMALVDLVSLDFFHNKMMSASLKLKKACYIALVLGSASMSVLALWA

**4. Brachypodium**

**>BdZIP1**

MSGSGCLPPGELDALSRVCRDGAAAARLKTGSLLAILLASAIGICLPVALTRAFRGREGYARGLLLVKCYAAGVILSTSLVHVLPDAYAALADCAVASRRPWRDFPFAGLFCLIGSLLALLVDVSASSHLEAHGHQPPEQEHEQPYAPIPKKAPTVFELAGEMSPRKRAVLDDREEPELHVSKNISGDQDRDDVALFGAKKGARLVRSDEVVVSTGGCHGGGHEVVEVGDGEEDEAMKKQKMVSKVLEIGIVFHSVIIGVTLGMSQDVCAIRPLVVALSFHQVFEGMGLGGCIAQAGFGMATVGYMCIMFSVTTPLGILLGMAVFHMTGYDDSSPNALIIEGLLGSLSAGILVYMALVDLISLDFFHNKMMSSSLKLKKVSYIALVLGSASMSILALWA

**>BdZIP2**

MAPAATSSWAAFLLWFCVAATAAWAHGGGGDDGDADGGGGGDKADLRAPGLVATKLWCLAVVFLGTLAGGVSPYFMRWNEAFLALGTQFAGGVFLGTAMMHFLSDANETFGDLVKDSAYPFAFMLACAGYVVTMLAECVISSVVARGRTTPDGAAAGSTSAGVLEEGKLGTTNGNSSEPQAADAHGSSTDHSVPSMLGNASTLGDSILLIAALCFHSVFEGIAIGVAETKADAWKALWTISLHKIFAAIAMGIALLRMLPNRPLLSCFAYAFAFAISSPIGVGIGIVIDATTQGRVADWIFAISMGLATGIFIYVSINHLISKGYKPQRPVAADTPVGRWLTVVLGVGVIAVVMIWDT

**>BdZIP3**

MLDTKHTLQVLPWILLFAQQAAASGCDCTAATDGADKQGAMKLKLVAIASILTAGAAGVLVPVLGRSLAALRPDGDIFFAVKAFAAGVILATGMVHILPAAFDGLTSPCLHKGGGGRNGFPFAGLVAMSAAMATMVIDSLAAGYYRRSNFSKARPIENVDIPGQAGEEEGRTEHVHHATHGHSHGEAVVVSSPEEASIADTIRHRVVSQVLELGILVHSVIIGVSLGASVRPSTIRPLVGALSFHQFFEGIGLGGCIVQANFKVRATIIMATFFSLTAPVGIVLGIAISSSYNVHSSTAFIIEGVFNSASAGILIYMSLVDLLATDFNNPKLQTNTKLQLMTYLALFMGAGMMSMLAIWA

**>BdZIP5**

MGMASRELALLCLFLGLLATLPALAVADCDCESDAAATGRDKARALRLKVIAIVCILAGSAIGAGIPSLGRRFPALRPETDLFLAVKAFAGGVILATGLVHILPTAFEALGSPCLVGHGPWRRFPFAGMVAMLAAIGTLIVDTVATGYFRRTNAKRAAAVTDEPALGGGRAGDLEATSSSDGHHAHAHGMSVLAAPPDGEDELVRHRVISQVLELGVVVHSLIIGMSLGASDFPSTVRPLVPALTFHQLFEGIGLGGCIVQAKFRLKSVLAMGLLFSLTTPVGIGVGIAISSVYDETSPKALVVQGLLEAAAAGILVYMALVDILAEDFTKASVQSRARLQLALNVSLLLGAGLMSLLAVWA

**>BdZIP6**

MSGSGCLPPGELDALSRVCRDGAAAARLKTGSLLAILLASAIGICLPVALTRAFRGREGYARGLLLVKCYAAGVILSTSLVHVLPDAYAALADCAVASRRPWRDFPFAGLFCLIGSLLALLVDVSASSHLEAHGHQPPEQEHEQPYAPIPKKAPTVFELAGEMSPRKRAVLDDREEPELHVSKNISGDQDRDDVALFGAKKGARLVRSDEVVVSTGGCHGGGHEVVEVGDGEEDEAMKKQKMVSKVLEIGIVFHSVIIGVTLGMSQDVCAIRPLVVALSFHQVFEGMGLGGCIAQAGFGMATVGYMCIMFSVTTPLGILLGMAVFHMTGYDDSSPNALIIEGLLGSLSAGILVYMALVDLISLDFFHNKMMSSSLKLKKVSYIALVLGSASMSILALWA

**>BdZIP7**

MAVLGGFRRNIGLFLSKSNGFMAASLSAASCAEEVEKAEGAACRDDAGALRLKWIAMAAILVAGVLGVGLPLVGRKRRAVRTGSAVFVAAKAFAAGVILATGFVHMLHDAEHALSNPCLPAAPWRRFPFPGFVAMLAALATLVLDFVVTRFYERKHRAEVARVKADAAAALAASTSATVSDEDITVVTVTDDEHKAPLLQTHSHSHSHAHSHGHELVQADGREGDVSEHVRSVVVSQILEMGIVSHSVIIGLSLGVSRSPCTIRPLVAALSFHQFFEGFALGGCIAQAQFKNLSAAMMASFFAITTPMGIAAGAGLASFYNANSPRALVVEGILDSVSAGILIYMALVDLIAADFLGGKMTGTPRQQVMAYVALFLGALSMSSLAIWA

**>BdZIP8**

MKPSAAILLGAAIAALLLVSAVRGEGEEDECGSAESAAADRARVRPLKIAAFFSILVSGALGCSLPVLARRVPGLRPDGDVFFLVKAFAAGVILATGFIHILPDAFENLGSPCLPSDGPWKDFPFAGLGAMVGAIGTLVVDTLATGYFTRAHSKKGGGAVVDEEKQAAAAAGEEDVHVHTHATHGHAHGSAALVAAVGGAEDDKMDTIRYRVISQVLELGIVVHSVIIGISLGASQEPDTIKPLVVALSFHQMFEGMGLGGCIVQAKFKARSIVTMILFFCLTTPVGIAVGVGISRVYNENSPTALVVEGGLNSVAAGILVYMALVDLLAEDFMNPKVQSRGKLQLGINLSMLLGAGLMSMLAKWA

**>BdZIP9**

MAANLKLSTFFLLLLVASSLPLLALAGDCECEASSEADDGGDDKASALNLKIIAVFSILVAGAAGCAIPSLGRRFPALGPDTNLFFAVKAFAAGVILATAFVHILPEAFDRLGSPCLEGHGPWRKFPFAGLVAMLAAIATLVVDTVATGYFQRAHGAKKLAPAVDGDDVEGSGSAADHRSHVHGHGASSAAVIASSSSAASHSHVDGAELIRHRIISQVLELGIVVHSVIIGMSLGASQNADTIRPLVIALTFHQFFEGIGLGGCIVQAKFRLRSVLAMALFFSLTTPVGVVIGIGISSGYNETSPRALVVQGLLSAAAAGILNYMALVDLLAEDFMNPRVQNNGRLQVVVNISLLLGTALMSMLAIWA

**>BdZIP10**

MAFFEGMESYAPYLHQFAASVSGTSCDRAAADEECRDDAAALRLKMVAVASILVAGAAGVAIPLVARKRRGGSGSGAGGGGTFVLAKAFAAGVILATGFVHMMHDAEEKFADPCLPSTPWRRFPFPGFVAMLAALGTLVVDFVGTSFYERKHRRDEEDASAAAAAARDESEATRFLLDDGVPSSRIAAAAVSGGDEKQDAMHIIGIRAHAAAHRHSHAHGHGACDGGAVFDGHGHGHEHGHADEEGPSQSRHVVVSQILELGIISHSVIIGLSLGVSQSPCTIKPLVAALSFHQFFEGFALGGCISEAQFKNFSALLMAFFFAITTPAGITVGAGIASFYNPNSPRALVVEGILDSMSAGILIYMALVDLIAADFLSRKMSCNPRLQVCSYVALFLGAMAMSSLAIWA

**>BdZIP11**

MKPSAAILLGAAIAALLLVSAVRGEGEEDECGSAESAAADRARVRPLKIAAFFSILVSGALGCSLPVLARRVPGLRPDGDVFFLVKAFAAGVILATGFIHILPDAFENLGSPCLPSDGPWKDFPFAGLGAMVGAIGTLVVDTLATGYFTRAHSKKGGGAVVDEEKQAAAAAGEEDVHVHTHATHGHAHGSAALVAAVGGAEDDKMDTIRYRVISQVLELGIVVHSVIIGISLGASQEPDTIKPLVVALSFHQMFEGMGLGGCIVQAKFKARSIVTMILFFCLTTPVGIAVGVGISRVYNENSPTALVVEGGLNSVAAGILVYMALVDLLAEDFMNPKVQSRGKLQLGINLSMLLGAGLMSMLAKWA

**>BdZIP13**

MKPSAAILLGAAIAALLLVSAVRGEGEEDECGSAESAAADRARVRPLKIAAFFSILVSGALGCSLPVLARRVPGLRPDGDVFFLVKAFAAGVILATGFIHILPDAFENLGSPCLPSDGPWKDFPFAGLGAMVGAIGTLVVDTLATGYFTRAHSKKGGGAVVDEEKQAAAAAGEEDVHVHTHATHGHAHGSAALVAAVGGAEDDKMDTIRYRVISQVLELGIVVHSVIIGISLGASQEPDTIKPLVVALSFHQMFEGMGLGGCIVQAKFKARSIVTMILFFCLTTPVGIAVGVGISRVYNENSPTALVVEGGLNSVAAGILVYMALVDLLAEDFMNPKVQSRGKLQLGINLSMLLGAGLMSMLAKWA

**>BdZIP16**

MESHVWVALALSFVGGLSTSLGALLVILNPTPDLKRLGLLQGFAAGLMLSISFLDLAHNALNSIGFLKANLWFFAGVLFFGFIVKFIPEPTFVPTDDVIRKKQTDDDGSGKDMMKKHRRQVLFSGIITAVGISLHNFPEGIAVFLGSVKGLRVGINLAIAIALHNIPEGVAVALPLYFATKSKWQAFKYATLSGFAEPLGVVFVAVFFPSNLNPEILEGLLASVGGVMAFLTLHELLPLAFDYAGQKQAVKAVFVGMAIMSASLYFLEISLPEEIGL

**5. Foxtail millet**

**>SiZIP1**

MSSRTHLLAVLVLLAAAPFVADAQPPAADPAADACADPSVDGACHNVPKALRLKLIAIPTILVASVIGVCLPLFSRSIPALRPDRNLFVIVKAFASGVILATGYMHVLPDSFNNLSSPCLPRKPWAEFPFTAFVAMLAALFTLMVDSLMLTFYNRKRSGGGNTSGRRAGAAVADHESPAHGHWHGHGHGHGHGHGDIVVAESGAVAKPDDDEARKVQLSRNRVVVQVLEMGIIVHSVVIGLGMGASQSVCTIRPLVAAMCFHQLFEGMGLGGCILQAEYGLKMKSGLVFFFSTTTPFGIALGLALTRVYRENSPTALIVVGILNAASAGLLHYMALVELLAADFMGPKLQGSVRLQLVSFAAVLLGAGGMSVMAKWA

**>SiZIP2**

MALAGLRRHAGQFLSTSNELMAASLSTATCAEEMQKAEGGGCRDDAVALRLKEVAMAAILVAGVLGVGLPLAGRKRRALRTDSSAFRAAKAFAAGVILATGFVHMLHDAQHALSSPCLPAAPWRRFPFPGFVAMAAALATLVLDFLATRFYETKHRDEAARVKAAAAATLAAASSASDEDITVVTVAEDDRKAPLLQTHCHGHSHGHGHNHGHGHELVQVEGREGDMSDHVRSVVVSQILEMGIVSHSVIIGLSLGVSRSPCTIRPLVAALSFHQFFEGFALGGCIAQAQFKNLSAVLMASFFAITTPAGIAAGAGLATFYNPNSPRALVVEGILDSVSAGILIYMSLVDLIAADFLGEKMTGSLRQQLVAYIALFLGALSMSSLAIWA

**>SiZIP3**

MRWNEAFLALGTQFAGGVFLGTALMHFLSDANETFGDLLPGSAYPWAFMLACAGYVVTTLADVVVSHVVSRGRTAPGSSAGGAELEEGKVSATNGTSSEPQPAEAHGSDHSVASMLHNANTLGDSILLIAALCFHSVFEGIAIGVAETKADAWKALWTISLHKIFAAIAMGIALLRMLPNRPFLSCFAYAFAFAISSPIGVAIGIVIDATTQGRVADWIFAISMGLATGIFVYVSINHLLSKGYKPRRPVAVDTPVGRWLAVVLGVAVIAVVMIWDT

**>SiZIP4**

MSGTGCFPDGGPAGSRACRDGAAAARLKTGSLLAILVASAVGICLPVALTRAFRGGPNYARGLLLVKCYAAGVILSTSLVHVLPDAQAALADCAVATRRPWRDFPFAGLFTLVGALLALLVDLSASSHLEAHGHGGGGGDGHGHGHQETTYAPIPKKAPVFELTGEMSPKKRAFLDDDQGDPAPHVFRNGADTDRDDVALFGAKKGAALVRSDEVAVVGGGCHGGGHEVLEVVGEGAGEEEEARRKQKMVSKVLEIGIVFHSVIIGVTMGMSQDVCAIRPLVVALSFHQVFEGMGLGGCIAQAGFGMATVGYMCIMFSVTTPLGILLGMLIFHMTGYDDSNPNALIMEGILGSLSAGVLIYMALVDLISLDFFHNKMMSASNKLKKACYIALVLGSASMSILALWA

**>SiZIP5**

MAEEADLELESFGFDEHDHAHHHHHHHGHHHHHDGMETSPMGVWLSAMGCSLLVSMASLICLVLLPVIFFQGKPSKAMVDALAVFGAGAMLGDSFLHQLPHAFGGGHSHSHDHEGHDHAQEHAHAHSLKDLSVGLSILFGIVLFFIVEKIVRYVEDNSQNGAHSMGHGHHHHHKRHDSSDKAKLNHQKSDGDGSLHESEATIRKRSSSGSTKATDGEPANSENHPAPDKALSSDVSSTSNSNLVFGYLNLFSDGVHNFTDGMALGSAFLLQGSVGGWSRTLFLLAHELPQEVGDFGILVRSGFSVSKALFFNFLSALVALAGTALALSLGKDPGHSSLIEGFTAGGFIYIAVAGVLPQMNDQKTTLKSSVVQLISLAMGMLVALGISLVE

**>SiZIP6**

MAPDLKLSAVFCLLAVASLPLLAVADCECEASTDEDSDKARALTLKIVAIFCILVASSVGCAIPSLGRRFPALRPDTDLFIAVKAFAAGVILATAFVHILPDAFEKLGSPCLVDGPWQKFPFTGLVAMLAAIATLVVDTIATGYFQRAHSAKTAAAPVIGDVEASDHAHGGHGHAHGVVSVMASTSNADGGGAQLIRHRVIAQVLELGIIVHSVIIGMSVGASESPSTIRPLVAALTFHQFFEGIGLGGCIVQAKFRLKSMLTMALFFSLTTPVGVVIGIGISSTYNENSPRALIVEGVLNAAAAGILNYMALVDLLAEDFMNPRVQNNGRLQVIVSVSLLVGAALMSMLAIWA

**>SiZIP7**

MAAVKHTFKVLSWLLLFAQLAFASTSNCTNATDGTETDKLGAMKLKLIAIASILTAGAAGVLVPVLGRSMAALNPDGDIFFAVKAFAAGVILATGMVHILPAAFDGLTSPCLYKVGRDRNVFPFAGLIAMSAAMATMVIDSLAAGYYRRSHFKKARPIDNLEIHEQPGDEERTGHAQHVHVHTHQTQGHSHGEVDIIGSPEEAAIADTIRHRVVSQVLELGILVHSVIIGVSLGTSVRSSTIRPLVGALSFHQLFEGIGLGGCIVQANFKLRATVMMAIFFSLTAPIGIALGIGISSSYNGHSATAFVVEGVFNSASAGILIYMSLVDLLATDFNNPKLQTNTKLQLMTYLALFLGAGMMSMLAIWA

**6. Barrel clover**

**>MtZIP1**

MMSSLTTTLKLLFFYVIFILPILVSCDSCKCETEQTKENSEKNEALHYKLGSIASVLVCGALGVSLPLLSKRIPILSPKNDIFFMIKAFAAGVILATGFIHILPDAFESLNSPCLKEKPWGDFPLAGLVAMLSSIATLMVDSFASSYYQKRHFNPSKQVPADEEKGDEHVGHVHVHTHATHGHAHGSATSSQDSISPELIRQRIISQVLELGIVVHSVIIGISLGTAQSIDTIKPLLVALSFHQFFEGMGLGGCISQAKFESRSTAIMATFFSLTTPIGIAIGMGVSSVYKDNSPTSLIVEGVFNSASAGILIYMALVDLLAADFMSPRMQNNFKIQIGANISLLLGSGCMSLLAKWA

**>MtZIP2**

MASFKTLKSTFLILCLLASFFLNPIKAHGGHDDSDHDSDNINIRSRSLVLVKIWCLIILFVFTFIGGVSPYYFRWNEVFLLLGTQFAGGVFLGTSMMHFLSDSNETFEDLTKKTYPFAFMLACSGYLLTMFGDCVVVYVTSNNQREAKVEELEGGRTPQEEEGTTELAMDESNVAFMKTTNVGDTILLILALCFHSVFEGIAVGISGTKEEAWRNLWTISLHKIFAAIAMGIALLRMLPKRPLITTAGYSFAFAISSPIGVGIGIAIDATTEGKTADWMYAISMGIACGVFVYVAINHLISKGFKPQRKSRFDTPWFRFLAVLFGVAVIAVVMIWD

**>MtZIP3**

MGLKNNNKVFVFSILIFLIIPTLIAAECTCDEEDLDRDKPKALRYKIAALVSILVASGIGVCIPLLGKVIPALSPEKDIFFIIKAFAAGVILATGFIHVLPDAFENLTSPRLKKHPWGDFPFTGFVAMCTAMGTLMVDTYATAYFQNHYSKKAPAQVENEVSPDVEKDHEGHMDVHTHASHGHAHPHMSSVSSGPSTELLRHRVITQVLELGIIVHSVIIGISLGASESPKTIRPLVAALTFHQFFEGMGLGSCITQANFKSLSITIMGLFFALTTPVGIAIGIGISSGYDENSPTALIVEGIFNAASSGILIYMALVDLLAADFMNPRMQKNGILRLGCNISLLLLGSGLMSLIAKWA

**>MtZIP4**

MAGSILWYTHVTMKNMNIYQYPIEKLRVQPVGLIVAFAVMATLAAECTCDEEDEERDRSKALRYKIAALVSILVASAIGVCLPLLGKVIPALSPEKDIFFIIKAFAAGVILSTGFIHVLPDAFENLTSPCLNEHPWGDFPFTGFVAMCTAMGTLMVDTYATAYFQNHYSKRAPAQVESQTTPDVENEEHTHVHAHASHSHAHGHISFDQSSELLRHRVISQVLELGIIGHSVIIGISLGASESPKTIRPLVAALTFHQFFEGMGLGSCITQANFKSLSITIMGLFFALTTPVGIGIGLGISNVYDENSPTAFIFEGIFNAASAGILIYMALVDLLAADFMNPRMQKNGRLQLGSNISLLLGAGCMSLIAKWA

**>MtZIP5**

MSTLFDEFMTNSSCESGESDLCRDESAALILKFVAMASILVAGFSGIAVPLLGNRRGLLRSDGEILPAAKAFAAGVILATGFVHMLQDAWKALNHSCLKSYSHVWSEFPFTGFFAMMSALLTLLVDFVATQYYESQHQKTHDRHGRVVGNGEGLEEELLGSGIVEVQGETFGGGMHIVGMHAHASQHGHSHQNHGDGHGHGHSHSFGEHDGVDSSVRHVVVSQVLELGIVSHSLIIGLSLGVSQSPCTMRPLIAALSFHQFFEGFALGGCISEARFKTSSATIMACFFALTTPLGVAIGTLVASNFNPYSPGALITEGILDSLSAGILVYMALVDLIAADFLSKKMRCSLRLQIVSFCLLFLGAGSMSSLALWA

**>MtZIP6**

MEVGVSFKCTAYSVTIHKAIFIVFILITFLTSQALADCESESTNSCNNKEKAQPLKLIAIFSILATSVIGVCLPLATRSIPALSPEGDLFIIVKCFAAGIILGTGFMHVLPDSYEMLWSDCLDEKPWHEFPFSGLVAMFSAVVTMMVDSIATSYYSKKGKSGVVIPESHGGDDQEIGHSHGGHHHIHNGFKTEESDEPQLLRYRVVVMVLELGIVVHSVVIGLGMGASNNTCSIKGILSAALCFHQMFEGMGLGGCILQAKYKFLKNAMLVFFFSITTPLGIAIGLAMSTSYKENSPVALITVGLLNASSAGLLIYMALVDLLAADFMSKRMQSSIKLQLKSYVAVFLGAGGMSLMAKWA

**>MtZIP7**

MSFSLRTFFFLSLLLLLFFSSVSSHGGHDDDADIDADSDSEAPHNLRSKSLILTKVYCLIVIFFATFIAGVSPYVLRWNEGFLILGTQFAGGVFLGTALMHFLSDANETFGDLTDKEYPFAYMLACAGYLITMLADCVISSLLEKPNHGAGADVEGQGVDKGRSNGVNSQSQYQSSAGTNDADLAPSSSIGDTVYIFIYVYIIALCAHSVFEGLAIGVSVTKADAWKALWTICLHKIFAAIAMGIALLRMVPNRPLLSCAAYAFAFAISSPIGVAIGIVLDSTTQGHVADWIFAISMGLACGVFIYVSINHLFAKGYVPHKHSKADSAYMKFLAVSLGIGVIAVVMIWDT

**7. Glycine max**

**>GmZIP1**

MMNFQACSNTISNLFYVCFVLLPTMALGDCTCDTIEATKSDSIEVLHYKIGSIASVLVAGALGVSLPLLSKRIPTLNPKNDIFFMVKAFAAGVILATGFVHILPEAYESLTSPCLKENPWGKFPFTGFVAMLSSIGTLMVDSFATGFYHRQHFNPSKQVPADDEEMGDEHAGHIHVHTHATHGHAHGSAVSSEGSITSDVIRQRIISQVLEIGIVIHSVIIGISLGTAGSIDTIKPLLVALSFHQFFEGMGLGGCISQAKFESKSMAIMATFFSLTTPIGIAIGMGVSSVYKENSPTALTVEGIFNSASAGILIYMALVDLLAADFMSPRLQKNLKLQLGANISLLLGAGCMSLLAKWA

**>GmZIP4**

MFLFEDLWSLPRLFSESLNLQSLRESFTSSSCDRTESEQCRDESAAMVLKFVAVASILVAGFGGVSIPLVGKSRRFLRPDGDVFAAAKAFAAGVILATGFVHMLRDSWDALREPCLGTHSRAWAKFPFTGFFAMVSALFTLLVDFLATEYYERREARGRVERGKVVDYDEGCDEALLETGIVEVKDLGRGGRHSHSHDGDDVESSVRHVVVSQVLELGIVSHSMIIGLSLGVSQSPCTMKPLIVALSFHQFFEGFALGGCISQAQFKTLSATIMSCFFALTTPLGVAIGASVASIFNPYSPVALITEGILDALSAGILVYMALVDLIAADFLSKKMRCNFRFQIICYCLLFLGAGLMSSLAIWA

**>GmZIP6**

MASACVTNATRAAACRDGAAAAHLKMISIFVIFVTSVAGMSSPVALAGIFRGKPLYDKAIVVIKCFAAGVILSTSLVHVLPDAYAALADCHVASRHPWRDFPFAGLVTLVGALLALVVDLAASSHVEQHAHAQYAPVEKEAAVELGGSAGDGDGEKGEELAKLKQRLVSQVLEIGIIFHSVIIGVTMGMSQNVCTIRPLVAALAFHQIFEGMGLGGCVAQAGFSFGTITYMCFMFAVTTPIGIILGMALFSLTGYDDSSPNALIMEGLLGSISSGILIYMALVDLIAVDFFHNKLMNSNRLLKKASFVALTLGSAAMSILALWA

**>GmZIP10**

MATSLTLFKTIFVFLIIFTLLTPQATADCEAESRNSCNNKKKALPLKIIAIFTILASSIIGISLPLVTRSVPALSPENNLFIIVKCFAAGIILGTGFMHVLPDSFDMLWSDCLKEKPWHEFPFSGLAAMFSAIITMMVDSLSTSIYTKKYRTTEVVPGESNRAGGGDQLEMAAVNLGHFHGHHHAHETKIEGKEAQLLRYRVVAMVLELGIIVHSVVIGLGMGASNNTCAIRGLIAAMCFHQMFEGMGLGGCILQAEYKFLKKVIMVVFFSVTTPFGIALGIAMSTTYKENSPSALITVGLLNASSAGLLIYMALVDLLSADFMSPRLQGSIKLQLKSYVAVFLGAGGMSLMAKWA

**>GmZIP11**

MSPSFCTSLFLFTLSLLFFLFFSLSVSAHSGHHDDGDADSDATPDLRARPLILAKVWCLIVIFIATFVSGVSPYILKWNEGFLVLGTQFAGGVFLGTAMMHFLSDANETFGDLTQKEYPFAFMLACAGYLMTLLADAVISSLFNNMGRHAQDVQGQGADVNKLSSNGVTSQSQHRSHDANHHLASPALGYVHSVGDTVLLIVALCAHSVFEGLAIGVAETKADAWKALWTICLHKIFAAIAMGIALLRMIPDRPLVSCAVYAFAFAISSPIGVAIGIILDATTQGHVADWIFAISMGLACGVFIYVSVNHLLAKGYMPHRPTKVDSAYFKFLAVFLGVGVIAVVMIWDT

**8. Tomato**

**>SlZIP2**

MIMTSIKKSTTLLTLFLFLFLKFLIINGHGGADDDDEHDSNGDTNTNLRAKGLILVKIYCLIILFVTTFVGGVSPYFYRWNEGFLLLGTQFAGGVFLGTSLMHFLSDSATTFGVLTEKEYPFAFMLASAGYLLTMFSDCIIMFVTKGGVNESSESKVEVDEEGRSTNIDEGHGTNPFLKTTSLGDTILLILALCFHSIFEGIAVGVSASKGEAWRNLWTISLHKIFAAIAMGIALLRMIPKRPFLLTCAYSFAFAISSPIGVGIGIAIDATSEGRTADWTYAISMAIACGVFIYVAINHLIAKGFKPQNKCYFDTQFFKFVAVLLGVGVIAVVMIWD

**>SlZIP3**

MSPQLFLLLKITTLIYLSILVQPTLTFANCTCEPQDNKYNHQTKHKALSYKLIAISSILCSSALGVILPILLKNFKSLQKNDYSPLQFMIKAFAAGVILATGFIHILPDAFESLTSPCLSEDLWGSFPFAGFVAMMSAIFTLMMESFASGYHRRAELRKAQPVNIGDEQGQDEHIDHGPQILLERSDSSSLMRHRLISQVLELGILVHSVIIGISLGTTENPKTIKPLIIALSFHQFFEGMGLGGCISQAKYKVRTIIIMVLFFTVTTPSGIAIGMMISKGYNEQSSTALIVQGVLNSASAGILIYMALVDLLATDFMNPKLYTSFKLQIVANVSLILGACCMSLLAKWGGT

**>SlZIP4**

MSFIEDLTSLFVMDHIRQKTGSLSDTIMLKVSESISNTACGSAVDEMEGCRDNSAALTLKIVAISAILIAGVCGVGIPLVGKKHRFLRTDSNLFFTAKAFAAGVILATGFVHMLPGATSSLTNPCLPKSPWSKFPFAGFIAMMAALATLVVDFVGTQYYEKKQEKQNQKDQTDSADLVSESAIVPVEPKPRNEKLFGEEEGGAIHIVGMHAHAAHHRHSHSHEHGDVREHSHGHSHSHSFGGGDEEGGVRHVVVSQVLELGIVSHSIIIGVSLGVSESPCTIRPLLVALSFHQFFEGFALGGCISQAQFSSLRSTVMATFFAITTPLGIAIGIGAASSYDPHSPRALVVEGILNSISAGILVYMALVDLIAADFLSKRMSCNTRLQVVSYFALFLGAGLMSLLAIWA

**>SlZIP5**

MAQFKKTIFWYILLVLPAIVLGDLGDCTCDPEDEYRNKKEALKYKMAALASILVASSIGVIIPVLGKAIPALSPERNLFFIIKAFAAGVILSTGFIHVLPDAYGSLTSPCLAKHPWGDFPFSGFIAMVSALATLMVDTYANSYYSKKNLENGVAVAQSGDEGGVVHPHSHGSGSMMVDSKSELLRYRVISQVLELGIIVHSVIIGIALGASETPKNIKPLVAALTFHQFFEGLGLGGCIAQAKLKSRTIAIMTLLFSLTTPIGIGIGLGITNVYDENSPTALIVEGVFNSASAGILIYMALVDFLAADFMHPRMQSNGKLQLGANFSLLLGAGLMSMLAIWA

**>SlZIP8**

MNKLVVSSLLFFFFLLPALVSSECTCDEDADDRDKIEALKYKLVAVASILIAGAIGVSIPILGKVIPAFRPENNVFFLIKAFAAGVILGTGFVHILPDAFESLSSPCLPEKPWGDFPFAGFIAMISSIGTMMVDLLATSFYKNSNLTKQKPVNSDEEKEVHVHTHSTHGHAHGSVMLTSSEGNDELDLSRRRVISQVLELGILVHSVIIGVSLGASESPKTIKPLVAALTFHQLFEGMGLGGCIAEAKFKIKKAALMAIFFSLTTPIGIAIGFGISTVYSETSPTALMVEGIFNSAAAGILIYMALVDLLAADFMSSRMQDSPKLLMGANIFLLFGAGCMSLLAKWA

**9. Barley**

**>HvZIP3**

MWATKHTLQALLPWLLLFVQQAAASGGCECTTATDGADKQGAMKLKLVAIASILAAGAAGVLVPVLGRSMAALRPDGDIFFAVKAFAAGVILATGMVHILPAAFDGLTSPCIHKGGGDRNGFPFAGLVAMSAAMATMVIDSLAAGYYRRSHFSKARPLDNIDIPGHTGDEEGRADHPHVHTHGHSHGEAIAVSSPEEAAIADTIRHRVVSQVLELGILVHSVIIGVSLGASVRPSTIKPLVGALSFHQFFEGIGLGGCIVQANFKVRATIIMATFFSLTAPVGIVLGIAVSSSYNVHSSTAFIIEGVFNSASAGILIYMSLVDLLATDFNNPKLQTNTKLQLMTYLALFLGAGMMSMLAIWA

**>HvZIP5**

MAANLKLAALCLLLVVSSLPLLARAECECEAGEEEEHDKAGALRLKIIAIFCILVASAAGCAIPTLGRKFPALSPEKDLFFAIKAFAAGVILATAFVHILPEAFERLGSPCLVDGPWQKFPFAGLVTMLGAIATLVVDTIATGYFQREHAKNSSAAIGNLDPADSEQAHGGHSHGVSAIIASSSCDDGAKLIRHRVISQVLELGIIVHSVIIGMSLGASENAGTIRPLVVALTFHQFFEGIGLGGCIVQARFRHKSFLMMTFFFSLTLPIGVVIGIGIASTYDENSPRALIAEGLLSAAAAGILIYMALVDLLAEDFMNPRVQNNGRLQVIINISLLVGIALMSMLAVWA

**>HvZIP6**

MSGRGCLPAGELAALSRVCRDGAAAARLKTGSLLAILIASAVGVCLPVALTRAFRGRDGYARGLLLVKCYAAGVILSTSLVHVLPDAYAALADCAVASRRPWRDFPFAGLLCLVGALLALLVDLSASSHLEAHGLHQQPQQQEGQPYAPIPTTKKAPVFELTGEMSPRKRAFLDESDRDDPAPRDDKNGGDPDRDDVALFGPKKGGRLPRSDEPLAPIVGCHGAGHEVVEVGEGEEEEARKKQKMVSKVLEIGIVFHSVIIGVTLGMSQDVCAIRPLVVALSFHQVFEGMGLGGCIAQAGFGMATVGYMCIMFSVTTPLGILLGMAVFHMTGYDDSSPNALIIEGLPGSLSAGILVYMALVDLISLDFFHNKMMSSSLKLKKASYIALVLGSASMSILALWA

**>HvZIP7**

MMIGVAGFRRHAGQLLSKSNGFIAASLSAASCADEVEKAEGAGCRDDAAALRLKWIAMAAILVSGVMGVGLPLAGRKRRTVETGSAVFVAAKAFAAGVILATGFVHMLHDAEHALSNPCLPAAPWRRFPFPGFVAMLAALATLVLDVLVTRFYETKHRAEVARVKADAAAALAAASTSASDEDITVVTVVQSEHKAPLLQAHSHSHAQSHGHELVQPQGREGEVSEHVRSVVVSQILEMGIVSHSVIIGLSLGVSRSPCAIRPLVAALSFHQFFEGFALGGCIAQAQFKNLSAVMMASFFAITTPTGIAAGAGLSSFYDANSPRALVVEGILDSVSAGILIYMALVDLIAADFLGGKMTGSARQQVMAYVALFLGALSMSSLAVWA

**>HvZIP8**

MAAPSLKLAALCLLLVVTLSLLPLLARAECECEAGGEEEQDKAGSLRLRIIAIFCILVASAAGCAIPSLGRRFPALSPDRDLFFGVKAFAAGVILATSFVHILPEAFERLGSPCLVDGPWQKFPFAGLVAMLAAIATLVVDTIATGYFQRAAHAKKAAAVVGADDVEATPAHHGLVGHSHGVSAVVASSAAAADDGGAQLIRQRVISQVLELGIIVHSVIIGMSLGASQSASTIRPLVVALTFHQFFEGIGLGGCIVQAKFRLKSVLLMALFFSLTTPVGVVIGIGISSVYNENSPNTLITQGILSAAAAGILNYMALVDLLAEDFMNPRVQSNGRLQVIVNLSLLLGTALMSMLAVWA

**>HvZIP10**

MAFIEEMESYYVPYLRAHLHQFAASVSTASCEEGAGDDEECRDEAAALRLKMVAVAAILIAGAVGVAIPLVGRRRRRGSGGEGASSGGGTFVLAKAFAAGVILATGFVHMMHDAEEKFADPCLPATPWRRFPFPGFIAMLAALGTLVMEFVGTRFYERRHGEEAAAAAATADDTTALLEDGTLAGIAAAAVSGDDEKQDAMHIVGMRAHAAAHQHSHAHGHDACDGGAVYDAHAHAHAHGHGHDHGHGSEERPSQAHHVVVSQILEMGIVSHSVIIGLSLGVSQSPCTIKPLVAALSFHQFFEGFALGGCISEAQFKSFSALLMAFFFAITTPVGITVGAGIASFYNANSPRALVVEGILDSVSSGILIYMALVDLIAADFLSRKMSCNPRLQVCSYVALFVGAIAMSSLAIWA

**>HvZIP13**

MKPSVAALLVSFVALLLVAAVRGDDDGCGPPESAGQDRARANHLKIAAFFSILVCGALGCSLPVLGRRVPALRPEGDVFFLVKAFAAGVILATGFIHILPDAFEKLTSPCLLPSDGPWHDFPFAGLGAMVGAIGTLVVDTVATGYFTRAQLNKDGAHGHGAITSSAAVVDEEKQAAAAASEEARRHEGGEQEVHVHTHATHGHAHGSAALVAAVGGAEDEKDTIRHRVISQVLELGIVVHSVIIGISLGASQNPDTIKPLVVALSFHQMFEGMGLGGCIVQAKFRARSIVTMILFFCLTTPVGIAVGFGISRVYNEYSPTALVVEGSLNSVAAGILIYMALVDLLAEDFMNPKVQSRGKLQLGINISMLVGAGLMSMLAKWA

**10. Mustard**

**>BrZIP1**

MSEYSFCFSTNMLRVYVVLMICMHVCCASSDCTSHDEHDVVSQEEAERATTLKLGSIALLLVAGGVGVSLPLIGKKIPALQPENDIFFLVKAFAAGVILCTGFVHILPDAFERLSSPCLETTAAGKFPFAGFVAMLSAMGTLMIDTFATAYYKRQHSMGNKQVSVVEDEEHAGHVHVHTHASHGHSHGSTELIRRRIVSQVLEIGIVVHSVIIGISLGASQSIDTIKPLMAALSFHQFFEGLGLGGCISLAELQSKSTVIMAAFFSVTAPLGIGIGMGMSSGLGYGRESKEAIMVEGMLNAASAGILIYMSLVDLLAPDFVNPRLQSNLWLHLAAFLSLLLGAASMSLLAIWA

**>BrZIP2**

MSFSSKTLRSTLFFLSILFLCFSLILAHGGSDHDEEEEAAGANQPPPAAGTTVVDLRSKSLVRVKIYCLIILFFSTFLAGISPYFYRWNESFLLLGTQFSGGIFLATALIHFLSDANETFRGLKHKEYPYAFMLAAGGYCLTMLADVAVAFVAAGSSNNNHNGASGAGESRVDDAVEVKEEGRRETGSSVDVNQTILRTSGFGDTALLIVALCFHSVFEGIAIGVSDTKSDAWRNLWTISLHKIFAAVAMGIALLKLIPKRPFFLTAVYSFAFGISSPLGVGIGIAINATSQGAAGDWTYAISMSIACGVFMYVSIHHLIAKGYKPREGRYFDKPIYKFLAVFLGVILLSIVMIWD

**>BrZIP3**

MVKNVKLLLFFISISLLLIAVAEAQDGHEGHSHSRNPKCECSHDHDQENKAGAQKYKIAAVPSVLIAGVIGVLFPLLGKVFPSLRPETPFFFVTKAFAAGVILSTGFMHVLPEAYEMLNSPCLTSEAWDFPFTGFIAMVAAILTLSVDTFATSSFNKSHCNASKTIPDGESGELVVDSAKVQMRRTRVIAQVLELGIIVHSVVIGISLGASQSPEAAKALFIALMFHQCFEGLGLGGCIAQGKFKCLSVTIMSTFFAITTPLGIVVGMEIANTYDESSPT

ALIVQGVLNAASAGILIYMSLVDLLAADFMHPKMQSNTGLQIMAHIALLLGAGLMSILAKWA

**>BrZIP4**

MIIVDILWKSFPLYLFSGSGTDPLSESILQMMASPKILCDAGESDLCRDDSAAFVLKFVAIASIFLSGAAGVAIPLVGKNRRFLQTEGNLFVSAKAFAAGVILATGFVHMLAGGTEALTNACLPEFPWSKFPFPGFFAMVAALITLLVDFMGTQYYERRQERSDNAAAPSEASAVVGVESGREEASVVVPVVVERVSDGNNKVFGEEEGGGMHIVGIRAHAAHHRHSHSNGLGTCDGHAHGHGHAHGHQDVGNGARHVVVSQILELGIVSHSIIIGLSLGVSQSPCTIRPLIAALSFHQFFEGFALGGCISQAQFKNKSATVMACFFALTTPVGIGIGTAVASSFNSHSPGALITEGILDSLSAGILVYMALVDLIAADFLSKRMSCNMRLQVVSYVMLFLGAGLMSALAIWA

**>BrZIP5**

MRITHNVKLLLFFFLISLLLISVSAGESKCECSHEEDEGNKAGARKYKIAAIPSVLVAGVIGVLFPLLGNFFPSLRPETNFFFVTKAFAAGVILATGFMHVLPEGYEKLTSPCLKGEAWEFPFTGFIAMVAAILTLSVDSFATSYFHRLHNKTSKKIGDGEEQIGGGGGGDVLGLHVHAHGHAHGIVGVDSGESEVQLHRTRVVAQVLEVGIIVHSVVIGISLGASQSPDTAKALFAALMFHQCFEGLGLGGCIAQGKFNCMSITIMSIFFSVTTPIGIAVGMGIASSYNESSQTALIVQGVLNAASAGILIYMSLVDFLAADFMHPKMQSNTGLRIMAHISLLIGAGIMSLLAKWA

**>BrZIP6**

MASCATGTEAAIRAAACRDGDAAAHLKLVSVLVIFLTSVFGISGPVLLARYFQGKPLYDKAVLVIKCFAAGVILSTSLVHVLPEAFESLADCQVSSRHPWKDFPFAGLVTLIGVITALLVDLTASEHMGHGGGGMEYIPVGGEAVGGLEMKEGKFGADLEIQERNEEEIVKMKQRLVSQVLEIGIIFHSVIIGVTMGMSQNQCTIRPLIAALSFHQIFEGLGLGGCIAQAGFKAGTVVYMCLMFAVTTPLGIVLGMMIFAATGYDDQNPNALIMEGLLGSLSSGILIYMALVDLIALDFFHNKMLTTADESGSTLKKLCFVALVLGSASMSLLALWA

**>BrZIP8**

MTTTTQHMNLIVVVLLIISFAIAPAISTVPKECETDSTNSCIDKTKALPLKIIAIVAILVTSMIGVTAPLFSRYVPFLSPDGKIFMVIKCFASGIILGTSFMHVLPDSFEMLSSPCLEDDPWHKFPFAGFVAMLSCLVTLAIDSIATSIYTRKDVCDGSEDTTNPLIIHIDHLQITTRETSSTCSKQLLRYRVIAMVLELGIIVHSVVIGLSLGATNDTCTIKGLIAALCFHQMFEGMGLGGCILQAEYTNVKKLVMAFFFAVTTPFGIALGIALSSVYKDNSPTALITVGLLNACSAGLLIYMALVDLLAAEFMGSMLQGSVKLQLICFGAALLGCGGMSVLAKWA

**>BrZIP9**

MSLLQDFWQFLQPLSSRFTESFTTSCDTGESDPCRDDAAALTLKYAAMASILIAGAAGVSLPLVGGTFLPSNGGLMRGAKAFAAGVILATGFVHMLSGGSEALSDPCLPEFPWRKFPFPEFFAMVAALLTLLADFMITGYYERKQEQLMNQSAQSLGPAEFGSGLSSGFLRDQEDGGKLHIVGMRAHADHHRHSLSMGPEGFEALANRSGGAGHGHDHGDVGLDSGVRHVVVSQILEMGIVSHSIIIGISLGVSHSPCTIRPLLLALSFHQFFEGFALGGCVAEAKLTPRGSAMMAFFFTITTPIGVAVGTAIASSYNSYSVVALVAEGVLDSLSAGILVYMALVDLIAADFLSKKMSVDFKLQIVSYCFLFLGAGMMSALAIWA

**>BrZIP10**

MMPKANVIFSSTITIFLLLSISHFPGAFSQSDKECTPEYDNTCTDKNKAFNLKLVAVFTILITSLIGVCLPLFARSVSAFQPERSLFLIVKSFASGIILATGFIHVLPDSFEMLSSHCLNDNPWHKFPFTGFVALISAVFTLMVDSITTSLFSKSGRRDPCADVASAGSPDEEMGHVSHYGHGLHHSNGKELGSNLQLLRYRVIAIVLELGIVVHSIVIGLSVGATNNTCTIKGLIAALCFHQMFEGMGLGGCILQAEYGWAKKAVMAFFFSVTTPFGVVLGMALSKTYKENGPDSLITVGLLNASSSGLLIYMALVDLLAADFMGQKMQRSIKLQLKSYAAVLLGAGGMAVLAKWT

**>BrZIP11**

MSRPLVFFFLFLVLVVPCLSHGGGGDQDDDEAPPAKSSDLKSKSLITVKIACLVIIFVLTFISGVSPYFLKWSQAFLVLGTQFAGGVFLATALMHFLSDADETFTDLLTAEGESEPSPAYPFAYMLACAGYMLTMLADSVIAHVYSKTPSKDVELQGAEKPNQGLGIGDSILLIVALCFHSVFEGIAIGISETKADAWRALWTITLHKVFAAIAMGIALLRMIPDRPLFSSIMYSFAFAISSPIGVAIGIVIDATTQGRVADWIFAVSMSLACGVFVYVSVNHLLAKGYLPNKKVNVDEPRFKFLAVLSGVVVIAIVMIWDT

**>BrZIP12**

MGCFSKTLILAFVFLLVTLPLLASATEVENECGGSNGRASAAEKATVLKYKIGAFFSILVAGVFGVCLPIFGLKSESNFFMFVKAFAAGVILATGFVHILPDATESLTSPCLGEEPPWGDFPMTGLVAMAGAILTMLIESFASGCLNRSRLEKKTLPVSTGGDKEEHSHIGSAHTHASQGHSHGSFLVPQDDLRKRIVTQILELGIVVHSVIIGISLGVSPSVSTIKPLLSAITFHQLFEGFGLGGCISEAKFGVKKIWIMVLFFALTAPAGIGIGIGVAEIYNENSPMALKVSGFLNAAAAGILIYMALVDLVAPLFMNHKAQSSMKIQLACSFALVLGAGLMSLLAVWA

**11. Cucumber**

**>CsZIP1**

MSKFFFLFLTLFLPALVSGVGASCSCDKSDGGGAVATKEAMKYKVGSIGSVLVAGAAGVSLPLVGKKIRCLRPENDIFFMIKAFAAGVILSTGFIHILPDAFQDLTSPCLGQNPWGDFPFAGFIAMAASIATLMVDTFATSFYQRRHFSKTKQVIADQETGNDHAGHVHVHTHATHGHAHGSAPTPTGELSLADLIRYRIISQVLELGIVVHSVIIGISLGASVSPATIKPLLVALSFHQFFEGMGLGGCISQAQFRWRSAAAMATFFSLTAPVGIAVGIGISGAYRDNSRTALIVEGSMNSASAGILIYMALVDLLAADFMNPRMQNNLGLQLGANISLLLGAACMSVLAKWA

**>CsZIP6**

MAAACPTDFTHDSACRDGRAAAHLKLISIFLIFITSVIGVSSPVLLTRFFHGKPLYDKAILTVKCFAAGVILSTSLVHVLPDAFDALSDCRIASQHPWRDFPFSGLVTMVGALVALFVDVTATSHVGHDQYNPVEEKGGEESGGEIGLLVAGERKSEETGGGGIIGEEESVKMKQKLVSKVLEIGIIFHSVIIGVTMGMSQNQCTIKPLVAALAFHQIFEGMGLGGCIAQAGFSFTTTAYMCFMFSVTTPMGIILGMILFSLTGYDDSNPKALIMEGLLGSFSSGILIYMALVDLIALDFFHNKLMTSNHCLKNICFIALLLGSTSMSILALWA

**>CsZIP7**

MARNLIFLLLLLSFISLSFSDSIPSPDSECEAQLQQDCHDRAESLKLKLISIATILVASMIGISLPLFSRAIPVLHPDGQTFAIVKAFASGVILATGYMHVLPDSYDFLTSPCLPENPWRKFPFPTFIAMLSAIMTLMLDSFSLSHFNKQSMQDQLSEEEEEINNEDRKEMSENLGKEEGTGEKLGSQLLRHRVIAQILEAGIVVHSVVIGLSLGASENPCTIRPLIAALCFHQLFEGMGLGGCILQAQYRIKMKAIMVFFFSVTTPFGIGLGIVLSNVYSENSPTALIVVGILNALSAGLLNYMALVNLLAHDFKGPKLQANLKLHIWAYVAVLMGVGGMSLLATWA

**>CsZIP11**

MLRFLFLLLLLLSSAAAHSGHSDDDDSAAGDSLSPSPNLRSKPLILVKITCLILIFFGTFIPGISPCFFKWNDGFLLLGTQFAGGVFFGTAMMHFLSDANETFRDLTDNAYPFAFMLACLGFLMTMAADCVISYLYRKPTADSSTDVELRGAATSPSKFQVQNGSNGHHTHPHQALTTMGSFGDSILLIVALCFHSVFEGIAIGVAETKADAWKALWTISLHKVFAAIAMGIALLRMIPNRPLLSSAAYSFAFAISSPIGIAIGIIIDATTQGAVADWIFAISMGLACGVFIYVSINHLLSKGYTPRDSVLVDNPNYKFLAVLLGIGVIAIVMIWDT

**12. Common wheat**

**>TaZIP1**

MAKMARSTRRTSNLNSTLLLLSLLLLTCFFQQASGHGGVDHGDGDEDDEGHHGDVGVRGGGLRSRGLIAVKVWCLVILLVFTFLGGVSPYFYRWNEAFLLLGTQFAAGIFLGTALMHFLAGSTSTFNALTHSPYPFSFMLACAGFLLTMLSDVAIVAVANRQRVNQAAPIQKDAEEEGESTSAGPVAAHAHPMLMTATSSFEDAILLIFALCFHSIFEGIAIGVSATKGEAWRNLWTIGLHKIFAAVAMGIALLRMIPKRPFLMTVLYSLAFAVSSPVGVGIGIAIDATAEGPAADWTYAISMGIATGIFVYVAINHLMARGYRPQQPNYFDKPIFKFLGVLTGVAVMAVVMIWD

**>TaZIP3**

MGATNHTLQALLPWLLLFVHQAAAASGGFECTTATDGADKQGATKLKLVAIASILTAGAAGVLVPVLGRSMAALRPDGDIFFAVKAFAAGVILATGMVHILPAAFDGLTSPCIYKGGGDRNGFPFAGLVAMSAAMATMVIDSLAAGYYRRSHFSKARPLDNIDIPGDEEGRADHPHVHAHGHSHGDAIVVSSPEEAAIADTIRHRVVSQVLELGILVHSVIIGVSLGASVRPSTIKPLVGALSFHQFFEGIGLGGCIVQANFKVRATIIMATFFSLTAPVGIVLGIAISSSYNVHSSTAFIIEGVFNSASAGILIYMSLVDLLAKDFNNPKLQTNTKLQLMTYLALFLGAGMMSMLAIWA

**>TaZIP5**

MKPSAAVLAAIVALLLVSAVRGDDACRSPESAAQDRARANPLKIAAFFSILVCGAMGCSLPVLGRRVPALRPEGDVFFLVKAFAAGVILATGFIHILPDAFDNLTSDCLPSDGPWKDFQFPFAGLGAMVGAIGTLVVDTVATGYFTRAHLNKDGANAAISSNAAGVDEEKQAAAEEARHHDGEEHDVHVHTHATHGHAHGSAALVAAVGGADDEKDTIRHRVISQVLELGIVVHSVIIGISLGASQNPETIKSLVAALSFHQMFEGMGLGGCIVQAKFKARSIVIMILFFCLTTPVGILIGFGISRVYNKNSPTALVVEGSLNSVAAGILIYMALVDLLAADFMNPKVQSRGKLQLGINVSMLVGAGLMSMLAKWA

**>TaZIP7**

MMIGVAGFSRHIGQLLSKSNGFIAASLSAASCADEAEKAEGAGCRDDAAALRLKWIAMAAILVSGVMGVGLPLAGRKRRTVQTGSAVFVAAKAFAAGVILATGFVHMLHDVEHALSNPCLPAGPWRRFPFPGFVAMLAALATLVLDVLVTRFYETKHRAEVARVKADAAAALAAASTSASDEDITVVTVVESEHKVPLLQAHSHSHAQSHGHELMQPQGREGEVSDHVRSVVVSQILEMGIVSHSVIIGLSLGVSRSPCTIRPLVAALSFHQFFEGFALGGCIAQAQFKNLSAVMMASFFAITTPTGIAAGAGLSSFYNANSPRALVVEGILDSVSAGILIYMALVDLIVADFLGGKMTGSPRQQVMAYVALFLGALSMSSLAVWA

**13. Sorghum**

**>SbZIP1**

MMMVRMRRRASTLLCTLLLLSLPCFHLASAHGGIDDGDNDGHATTPPADADPSSVHQHLRSKGLIAVKVWCLVILLIFTFLGGVSPYFYRWNEAFLLLGTQFAAGVFLGTALMHFLADSTSTFHGLTKNQYPFSYMLACVGFLLTMLADCVVAAVTKRSAAGGGGGQRVVNEAAREEGDARHQTQTKQEDAAHARHPMLVTVRTASFEDAVLLIFALCFHSIFEGIAIGVSATKSDAWRNLWTIGLHKVFAAVAMGIALLRMIPKRPFLMTVAYSLAFAVSSPVGVGIGIGIDATAEGRAADWTFAISMGFATGVFLYVAINHLIAKGYRPQEPTRVDSPSFKFLGVLLGVAVMAVVMIWG

**>SbZIP2**

MARATNAHYHLHLLLLLCLSLAAAAWAHGGGGGDGDGDSDDADADAPRPDLRARGLVEAKLWCLAVVFAGTLLGGVSPYFLRWNEAFLALGTQFAGGVFLGTALMHFLSDADETFGDLLPDSGYPWAFMLACAGYVVTMFADVAISYVVSRSRTAGSSSGSAAAGLEEGKMGATNGTISDPTPPETHGSDHSAASMLRNASTLGDSVLLIVALCFHSVFEGIAIGVAETKADAWKALWTISLHKIFAAIAMGIALLRMLPNRPLLSCFAYAFAFAISSPIGVGIGIVIDATTQGRVADWIFAVSMGLATGVFVYVSISHLLSKGYKPRRPVAVDTPVGRWLAVVLGVAVIAVVMIWDT

**>SbZIP3**

MGAVKHTLKVFPWLLLFAQLAVATTSKCTNATNGTETDSLGAMKLKLIAIASILTAGAAGVLVPVLGRSMAALHPDGDIFFAVKAFAAGVILATGMVHILPAAFDGLTSPCLYKGGSGGNIFPFAGLIAMSAAMATMVIDSLAAGYYRRSHFKKARPIDNLEIHEQPGDEERTGHAQHVHVHTHATHGHSHGEADGINSPEEASIADTIRHRVVSQVLELGILVHSVIIGVSLGASVRPNTIRPLVGALSFHQFFEGIGLGGCIVQANFKLRATVMMAIFFSLTAPIGIALGIAISSSYNGHSTTAFIVEGVFNSASAGILIYMSLVDLLATDFNKPKLQTNTKLQLMTY

LALFLGAGMMSMLAIWA

**>SbZIP4**

MDAARARAPLPSSLAWLAVLLLLVVATTTPGRVGAASSAATSASEAAAGCDCGGAAEAEAAIIKEEDARGALRLKLIAVASILASGAAGVLVPLLGRSASALRPDGDVFFAVKAFAAGVILATGMVHILPAAFDALGGGGGFPFAGLVAMCSAMVTMMVDSVAAGYYQRSHFRKALPVDDATDGAARAVPGDEEGAAAAGHAGHVHVHTHATHGHAHGQAHDHGGHGHAGPPSPQDASSVAVSIRHRVISQVLELGILVHSVIIGVSLGASLRPSTIRPLVGALSFHQFFEGIGLGGCIVQAKFKARATVIMATFFSLTAPMGIALGIAITSSYSKHSDTALVVEGVFNAAAAGILIYMSLVDLLAADFSNPRLQTNMKLQLATYIALFLGAGLMSLLAKWA

**>SbZIP5**

MAKLAVVAAALYAILVLAVSLPVLVTAAECDCGSDDAAAAGRRDKAGALRLKVVAIFCILAGGAVGAAVPSLGHGRLPALRPDADLFLAVKALAGGVILATGLVHILPAAFDALGSPCLAAGPWNRFPFAGMVAMLAAVATLVVDTVATGYFRRRTVARRKAAAAVGDEPSSSELGRCDGGDLEAEASDDSGAHHGHVHGMSALAPAPTTTTVDDELVRHRVISQVLELGVVVHSLIIGMSLGASDFPSTVRPLVPALTFHQLFEGIGLGGCIVQAKFRLRSMLAMAVFFSLTTPIGVAIGIGISSVYDETSPTALVVQGFLEAAAAGILVYMALVDILAEDFMSARVQSSARLQVALNTSLLLGAGLMSMLAIWA

**>SbZIP6**

MSGTGCLPADGAAALTRVCRDGAAASRLKTGSLLAILVASAVGICLPVALTRAFRGSPNYARGLLLVKCYAAGVILSTSLVHVLPDAHAALADCAVASRRPWRDFPFAGLFTLVGALLALLVDLSASSHLEAHAHVGADADAHHGHGHQETPYTPIPKKVPVFELAGEMSPKKRAFLDDDREEDPAPHAATNGGADPDRDDVALFGPKKGARSDEVPVVVAAGCHGVAHEVVEVGEGPGEDEEETRRKQKMVSKVLEIGIVFHSVIIGVTMGMSQDVCAIRPLVVALSFHQVFEGMGLGGCIAQAGFGMATVGYMCIMFSVTTPLGILLGMLVFHMTGYDDSNPNALIMEGILGSLSAGILIYMALVDLISLDFFHNKMMSASLKLKKACYIALVLGSASMSVLALWA

**>SbZIP7**

MALGGLVGQFLASSNELMVASLSAVSCADEVQEEGAEGAGCRDDAAALRLKEVAMAAILVAGVLGVGLPLVGRKRRAMRTDSAAFLAAKAFAAGVILATGFVHMLHDAGTALSSPCLPAVPWRRFPVPGFVAMAAALATLVLDFLATRFYEAKHRDEAARVKAAAAAALVATTSSASDEDITVLTVDAEDERKAPLLQTHCHGHGHGHSHSHGHELVQVVGSEAEVSAHVRSIVVSQILEMGIVSHSVIIGLSLGVSRSPCTIRPLVAALAFHQFFEGFALGGCIAQAQFKNLSAILMASFFAITTPAGIAAGAGLTTFYNPNSPRALVVEGILDSVSAGILIYMSLVDLIAADFLGGKMTGSLRQQVVAYIALFLGALSMSSLAIWA

**>SbZIP8**

MRSSRAVLVFALLLLLVARAAAADGGDACGAGGEAVQAGDRARAKALKIAAFFSILVCGALGCCLPVLGRRVPALRADGDVFFLVKAFAAGVILATGFIHILPDAFEKLTSDCLPKSGPWQDFPFAGFGAMVGAIGTLVVDTVATGYFTRVHFKNGAAAAEAAAVGDEEKQQAAAAAAAPHGDDDHDHDGHVHMHTHATHGHAHGSSALVAAVGGTEGDKEHALRHRVIAQVLELGIVVHSVIIGISLGASEGPSTIKPLVVALSFHQMFEGMGLGGCIVQAKFKVRSIVTMVLFFCLTTPVGILVGIGISSVYNEDSPTALIVEGILNSVAAGILVYMALVDLLAEDFMNPKVQSRGKLQLAINVSMLVGAGLMSMLAKWA

**>SbZIP9**

MAAHPKLAALCCLLAVASLPLLAVADCECEASTGEEDDKSRALTLKIVAIFCILVASSVGCAIPSLGRRFPALRPDTDLFFAVKAFAAGVILATAFVHILPDAFEKLGSPCLVDGPWQKFPFTGLIAMLAAIATLVVDTIATGYFQRAQAAKTAAVVVVGDVETSGGHAHGGHGHGHGHGHTHGMSSVVAAAATTSNGDDSTQLIRHRVISQVLELGIIVHSVIIGMSVGASESPSTIRPLVAALTFHQFFEGLGLGGCIVQAKFRLKQVLMMTLFFSFTTPIGIVIGIGISSAYDENSPNALIIEGVLDAAAAGILNYMALVDLLAQDFMNPRVQNNGRLQVIINISLLVGTALMSMLAVWA

**>SbZIP10**

MPFFEEMMEGSSYIRTHLQQIAAASVSTASCGGASNTDADDAECRDEAAALRLKMVAVAAILVAGATGVAIPLVGRRCRGRGGGASSSSGSFSSSPSAGGAFVLVKAFAAGVILATGFVHMLHDADEALTDPCLPAAPWRRFPFPGFVAMLAALATLVFDFVGTHMYESKQHSADAAEAAAAAGNASVNASGHDVTAALLEDGALAGSVASGIGYGALMGSVGSSIGYSALMGSVGSSIGGGHMDPMHIVGMRAHAAAHRHSYSHGIGPCDDGHNGNDEEPSQARHVVVSQILELGIVSHSVIIGLSLGVSQNPCTIKPLVAALSFHQFFEGFALGGCISEAQFKGFSTLLMAFFFAITTPTGITVGAGIASFYNPNSPRALVVEGILDSISAGILIYMALVDLIAADFLSKRMSCNLRLQVCSYIALFLGAMSMSSLAIWA

**14. Panicgrass**

**>PhZIP1**

MRRASSALLCTLLLLLSSLLLQASAHGGIDDGDGGEVDTPTPRPDSSTPIKQGRLIAVKVWCLVILFVFTFLAGVSPYFYRWSEAFLLLGTQFAAGVFLGTALMHFLADSASTFHGLTRNHYPFSYMLACVGFLLTMLADCVIAGVTKRSRGDRERAVNDAEEAEQQVEDGDAQGQDKEHQHPTMLLVRTSSLEDAILLILALCFHSIFEGIAIGVSATRSDAWRNLWTIGLHKIFAAVAMGVALLRIIPKRPLVTTVAYSMAFAVSSPVGVGIGIGIDATAEGRAADWSYAIAMGLATGVFVYVAINHLIGKGYRPQEPTGADRPLLKFLAVLLGAGVMAVVMIWD

**>PhZIP2**

MARGTNARHHHLHLHLLLCLSLAATTAWAHGGGADADADADGAGAGSPEPDLRARGLVAAKLWCLAVVFAGTLLGGVSPYFMRWNEAFLALGTQFAGGVFLGTALMHFLSDADETFGDLLPDSGYPWAFMLACAGYVVTTLADVVISHVVSRGRTAPGSRGGGGGGDGAGLEEGKVSTTNGTSSEPQPAVAHGSDHSVASMLRNASTLGDSVLLIAALCFHSVFEGIAIGVAETKADAWKALWTISLHKIFAAIAMGIALLRMLPNRPLLSCFAYAFAFAISSPIGVGIGIVIDATTQGRVADWIFAVSMGLATGIFVYVSINHLLSKGYKPRRPVAVDTPVGRWLAVVLGVAVIAVVMIWDT

**>PhZIP5**

MAKLQGALWCLLLALAVSLLPAPAAAECDCSEDAAGRDKARALRLKVVAIFCILAGGAAGAAVPALGRRVPALRPGTGLFRAVKAFAGGVILATGLVHILPAAFDALGSPCLADTGPWRRFPFAGMVAMLAAIATLVVDTIATGYFRRTVARKAAAVVDEPPAEPGRCGDGDLEGAADGHHGHAHGMSVLAPAPPAAGDDLVRHRVVSHVLELGVVVHSLIIGMSLGASDFPSTVRPLVPALTFHQLFEGIGLGGCIVQAKFRLKSVVAMALFFSVTTPAGVAIGIAISSVYDETSPTALVVQGLLEAAAAGILVYMALVDILAEEFMSARVQSRARLQLALNASLLLGAGLMSMLAIWA

**>PhZIP6**

MSGTGCFPAGGPDGSRACRDGAAAARLKTGSLLAILVASAIGICLPVALTRAFRGSPNYARGLLLVKCYAAGVILSTSLVHVLPDAHAALADCAVATRRPWRDFPFAGLFTLVGALLALLVDLSASSHLEAHGHGGGGHGHQETPYAPIPKKAPVFELTGEMSPKKRAFLDDDQEDLAPRAARNGAETDRDDVALFGAKNGAAVVRSDEVAAVGGGCHGVGHEVVEVGDGAGEEDEARRKQKMVSKVLEIGIVFHSVIIGVTMGMSQDVCAIRPLVVALSFHQVFEGMGLGGCIAQAGFGMATVGYMCIMFSVTTPLGILLGMFVFHMTGYDDSNPNALIMEGILGSLSAGVLIYMALVDLISLDFFHDKMMSASIKLKKACYIALVLGSASMSILALWA

**>PhZIP7**

MALAGVRRHAGQFLSTSNELMAASLSTASCAKEMQKAEGGGCRDDAAALRLKEVAMAAILVAGVLGVGLPLAGRKRRAMRTDSAAFMAAKAFAAGVILATGFVHMLHDAQHALSSPCLPAVPWRRFPFPGFVAMAAALATLVLDFLATRFYETKHRDEAARVKAAAAAALAAASSASASDEDITVVTVVEDERKAPLLETHCHGHSHGHRHSHSHGHELVQAEGREGDVSEHVRSVVVSQILEMGIVSHSVIIGLSLGVSRSPCTIRPLVAALSFHQFFEGFALGGCIAQAQFKNLSAVLMASFFAITTPAGIAAGAGLSTFYNPNSPRALVIEGILDSVSAGILIYMSLVDLIAADFLGSKMTGSLRQQVMAYIALFLGAISMSSLAIWA

**>PhZIP8**

MRPPCAALVLAAVAALLLLLLAPAARADDGGCGADGAAAARGGDLARARALKIAAFFSILVCGALGCCLPVLGRRVPALRADGDVFFLVKAFAAGVILATGIIHILPDAFEKLTSECLPAGGPWKDFPFAGFGAMVGAIGTLVVDTVATGYFTRLHFKDSAAAAATAAVSAAVVGDDVEKQQQQATAAAPHAGGSDDHEGHVHMHTHATHGHAHGSTALVAAVGGGEGDKEHALRHRVIAQVLELGIVVHSVIIGISLGASKDPSTIKPLVVALSFHQMFEGMGLGGCIVQAKFKLRSIVTMVLFFCLTTPVGILVGIGISSVYNEDSSTALVVEGILNSVAAGILIYMALVDLLAEDFMNPKVQSRGKLQLGINVSMLVGAGLMSMLAKWA

**>PhZIP9**

MAAADLKLAACLCLLAVASLPMLAIAECECTASGDEDSDKGRALTLKIIAIFSILVASSVGCAIPSLGRRFPALRPESDLFIAVKAFAAGVILATAFVHILPDAFEKLGSPCLVDGPWQKFPFAGLVAMLAAIATLVVDTIATGYFQRAHAKSTAAVGDLETPDHAHHGHGHSHGMPALIASSSSTSNTDEGAKLIIRHRVISQVLELGIIVHSVIIGMSLGASESPSTIRPLLAALTFHQFFEGIGLGGCIVQARFRLRSVVTMALFFSLTTPVGVAIGIGISSAYNENSPSALITEGVLTAAAAGILNYMALVDLLAEDFMNPRVQNNWKLQVILSVALLLGTALMSMLAIWA
